# Strategies to Assure Optimal Trade-Offs Among Competing Objectives for the Genetic Improvement of Soybean

**DOI:** 10.3389/fgene.2021.675500

**Published:** 2021-09-24

**Authors:** Vishnu Ramasubramanian, William D. Beavis

**Affiliations:** ^1^George F. Sprague Population Genetics Group, Department of Agronomy, Ames, IA, United States; ^2^Bioinformatics and Computational Biology Graduate Program, Iowa State University, Ames, IA, United States

**Keywords:** island model selection, recurrent selection, tradeoffs, optimization, genetic algorithms, genetic response, genomic selection, recurrence models

## Abstract

Plant breeding is a decision-making discipline based on understanding project objectives. Genetic improvement projects can have two competing objectives: maximize the rate of genetic improvement and minimize the loss of useful genetic variance. For commercial plant breeders, competition in the marketplace forces greater emphasis on maximizing immediate genetic improvements. In contrast, public plant breeders have an opportunity, perhaps an obligation, to place greater emphasis on minimizing the loss of useful genetic variance while realizing genetic improvements. Considerable research indicates that short-term genetic gains from genomic selection are much greater than phenotypic selection, while phenotypic selection provides better long-term genetic gains because it retains useful genetic diversity during the early cycles of selection. With limited resources, must a soybean breeder choose between the two extreme responses provided by genomic selection or phenotypic selection? Or is it possible to develop novel breeding strategies that will provide a desirable compromise between the competing objectives? To address these questions, we decomposed breeding strategies into decisions about selection methods, mating designs, and whether the breeding population should be organized as family islands. For breeding populations organized into islands, decisions about possible migration rules among family islands were included. From among 60 possible strategies, genetic improvement is maximized for the first five to 10 cycles using genomic selection and a hub network mating design, where the hub parents with the largest selection metric make large parental contributions. It also requires that the breeding populations be organized as fully connected family islands, where every island is connected to every other island, and migration rules allow the exchange of two lines among islands every other cycle of selection. If the objectives are to maximize both short-term and long-term gains, then the best compromise strategy is similar except that the mating design could be hub network, chain rule, or a multi-objective optimization method-based mating design. Weighted genomic selection applied to centralized populations also resulted in the realization of the greatest proportion of the genetic potential of the founders but required more cycles than the best compromise strategy.

## Background

Responses to the selection of commodity crops have been enabled by decreasing the number of years per cycle of recurrent selection, by increasing the number of replicable genotypes (selection intensity), and by increasing the number of replicated field trials (heritability on an entry mean basis). In other words, genotypic improvements from responses to selection in commodity crops over the last 50 years (Specht et al., 2014) required monetary investments that became part of increased seed costs during the same time (Byrum et al., 2017; USDA-ERS, 2020). Since the emergence and adoption of Genomic Selection (GS), it has been possible to increase the numbers of genotypes that are evaluated, i.e., selection intensity, without significant increases in numbers of field plots (Bernardo and Yu, 2007; Bernardo, 2008; Asoro et al., 2011; Heslot et al., 2012; Nakaya and Isobe, 2012; Combs and Bernado, 2013; Crossa et al., 2014; Beyene et al., 2015; Bassi et al., 2016; Marulanda et al., 2016; Jonas and de Koning, 2016; Hickey et al., 2017; Goiffon et al., 2017).

While the initial interest in GS has been to increase genetic gains, plant breeders are aware that increased selection intensities are associated with faster losses of genetic potential in the founder populations (Robertson, 1960; Hill and Robertson, 2008; Bulmer, 1971).

**FIGURE 1:**
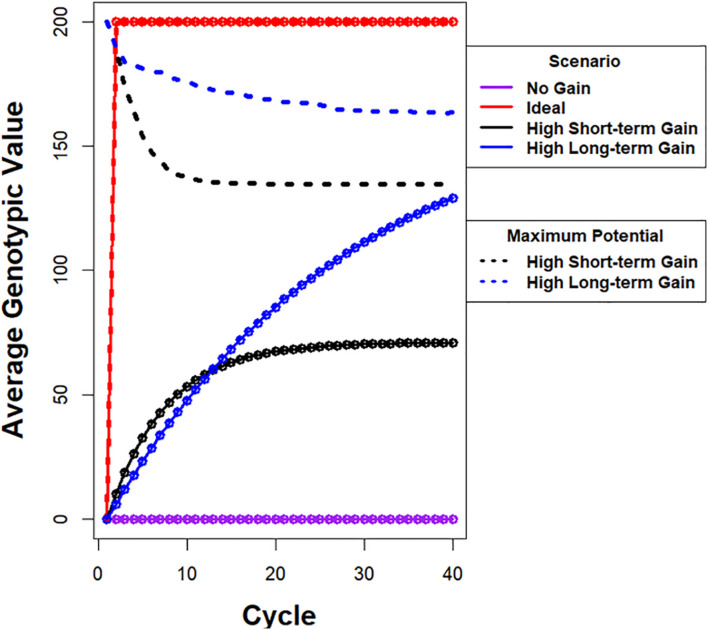
Illustration of possible responses to recurrent selection. Average genotypic value is plotted on *y*-axis and cycle number is plotted on *x*-axis. (i) The purple line depicts a response pattern with no genetic gain, which is possible when there is no selection in a population with a large effective population. (ii) The red line depicts a hypothetical ideal response curve when the maximum genotypic potential among founders is realized in one cycle of selection. The ideal is not feasible using selection if the alleles responsible for the maximum are distributed among more than two founders. (iii) The black line depicts a response pattern with high rates of gain in the early cycles, but the maximum average genotypic value approaches a limit that is less than 40% of the genotypic potential among the founders. The dashed black line represents the corresponding rapid drop in genotypic potential among the founders. (iv) The blue line depicts a response pattern with slightly slower rates of gain in early cycles relative to the black line, but achieves greater genotypic values before approaching a limit. This is due to conservation of genetic variability as represented by the dashed blue line with slower rate of decrease in genotypic potential of the population. Note that there are potentially an infinite number of unique response curves that fall between no response and an ideal response.

Between the two limiting cases of no response to selection and the infeasible ideal response of realizing maximum genotypic potential among founders in a single cycle of selection, there are many possible recurrent selection response curves, two of which are illustrated in Figure 1. One of the curves depicts high rates of gain in the early cycles, which is favored for immediate short-term gains. However, the maximum average genotypic value approaches a limit that is less than 40% of the genotypic potential of the founders. The other curve depicts a response with slower rates than the previous one in early cycles, but with greater genotypic values before approaching a limit due to loss of genetic potential from selection. This response pattern is desirable for maximizing gains while preserving genetic variability. For a fixed set of evaluation resources, the differences between the two response curves could be due to differences in selection intensities or selection methods or both. For example, simulation studies of recurrent GS methods indicate that GS provides faster genetic gains than phenotypic selection (PS) for five to 10 cycles of recurrent selection; PS provides continued genetic gains after response to GS becomes limited (Goddard, 2009; Jannink, 2010; Liu et al., 2015). A question for the breeder is which possible curve most accurately represents the relative importance of short-term gains versus retention of valuable alleles for future generations of plant breeders. For commercial plant breeders, competition in the marketplace forces greater emphasis on maximizing immediate genetic gains. In contrast public plant breeders have an opportunity, perhaps an obligation, to place greater emphasis on minimizing the loss of useful genetic alleles while realizing genetic gains that are close to the maximum.

In spite of these general statements about the relative importance for commercial and public genetic improvement projects, each genetic improvement project has unique objectives and constraints. Previously, we (Ramasubramanian and Beavis, 2020) reported responses for combinations of selection intensity, GS methods, and training sets applied recurrently to populations composed of 2000 F_5_-derived lines from contemporary soybean germplasm belonging to maturity groups II and III. The combinatorial set of factors consisted of Phenotypic Selection (PS) and four commonly used GS methods, training sets, selection intensity, number of QTL (nQTL), and broad sense heritability (H) on an entry mean basis. While interactions among all factors affected all response metrics, only the impacts of GS methods, selection intensity, and training sets are factors that plant breeders can control.

All GS methods provided greater responses than PS for at least five cycles, but PS provided better responses to selection as response from GS methods reached a limit. These results are consistent with reports by Goddard (2009); Jannink (2010), and Liu et al. (2015) that demonstrated that the full genotypic potential of the founders is eliminated more quickly with GS than PS. In terms of factors that a soybean breeder can control, a selection intensity of 1.75 and Ridge Regression Genomic Prediction (RRGP) models provided rapid response in the early cycles of selection. It also allowed the retention of genetic diversity for continued response to selection in later cycles, when the models are updated with training data from previous cycles. We also suggested that further improvements might be made if the populations were organized into families or islands and mating designs that optimize parental contributions to retain greater genetic potential in the populations are used (Ramasubramanian and Beavis, 2020). Herein, we investigate strategies that soybean breeders can employ to find optimal trade-offs between maximizing genetic gain from selection and retaining useful genetic diversity.

Given that there are constraints on the size of the breeding program, including the number of lines to evaluate and the number of field plots, it is important to reveal as many response curves as possible for possible breeding strategies. While breeders can observe these curves and identify one that most closely reflects the relative importance of the two objectives, we conjectured that it should be possible to design additional breeding strategies that are better, in the sense of minimizing the trade-offs, than those we previously investigated. One of the approaches is to use a trade-offs table to identify the best strategy for a given set of relative weights for the short-term and long-term objectives of the program.

The challenge of realizing genetic gains from selection and retaining useful genetic diversity in closed populations has been of interest since it was demonstrated that there are theoretical limits for response to selection in closed populations (Hill and Robertson, 2008; Bulmer, 1971). Trade-offs among objectives don’t prohibit finding optima as long as optimality is defined as a compromise among competing objective functions (Deb, 2003; Konak et al., 2006; Shoval et al., 2012; Sheftel et al., 2013; Saeki et al., 2014).

Before the development of GS, quantitative geneticists working on domestic animal systems utilized mathematical programming modeling and operations research approaches to find near-optimal solutions to the challenge of assuring genetic gain and minimizing inbreeding per cycle of selection (Wray and Goddard, 1994). The first publication using operations research approaches to address multiple objectives in plant breeding was applied to the selection of multiple traits (Johnson et al., 1988). Generally, operations research approaches involve three activities: (1) define objectives using measurable metrics; (2) translate the objectives into a model consisting of objective functions, decision variables, and constraints; and (3) find an algorithm that will provide values for the decision variables resulting in optimal solutions to the model (Rardin, 2017).

If a genetic improvement project wants to assure genetic gain and retain useful genetic diversity, then there are two competing objectives for which a trade-off needs to be optimized. This represents an example of a multi-objective optimization problem (Deb, 2003, 2011; Rardin, 2017). After translating each of the objectives into an objective function, there are several strategies for finding the optimal solution (Deb, 2003). The two most commonly used strategies are known as ε-constraint and the weighted sum. The ε-constraint method consists of identifying one of the objectives, e.g., maximize genetic gain, and translate other objectives, such as minimize inbreeding, into decision variables that can be constrained in a linear, integer, or quadratic mathematical programming model (Haimes et al., 1971); in other words, translate the multi-objective optimization mathematical model into a single objective optimization model for which there exist computational algorithms capable of finding the optimum solution (Frank and Wolfe, 1956; McCarl et al., 1977; Lazimy, 1982). Framing the ε-constraint method requires definition of metrics for genetic diversity or inbreeding. In animal breeding, this method became known as Optimum Contribution Selection (Wray and Goddard, 1994; Brisbane and Gibson, 1995; Meuwissen, 1997; Grundy et al., 1998; Meuwissen et al., 2001). Subsequent to the development of GS, Optimum Contribution Selection was modified to maximize Genomic Estimated Breeding Values (GEBVs), and the realized relationship matrix was used to constrain inbreeding in what became known as Genomic Optimum Contribution Selection (Sonesson et al., 2010; Schierenbeck et al., 2011; Woolliams et al., 2015).

The second well-established approach to a challenge is known as the weighted sum method. The weighted sum method assigns weights, ω_*i*_∈ [0, 1] and ∑ω_*i*_ = 1, to each of the “*i*” objective functions, and an algorithm is employed to find the values for the decision variables that minimize all objective functions simultaneously (Zadeh, 1963). The weighted sum method is equivalent to the concept of selection index familiar to breeders. In this case, the selection index is composed of weighted parameters for genetic gain and inbreeding or, equivalently, genetic diversity. If genomic information is available, GEBVs can be used to maximize genetic gain and the realized relationship matrix can be used to minimize inbreeding resulting in a genomic selection index that can be calculated for all genotypes (Carvalheiro et al., 2010; Clark et al., 2013).

Both ε-constraint and weighted sum methods are referred to as preference methods (Deb, 2003) where the constraints or relative weights have been predetermined. For defined preferences, there exist exact optimization algorithms if Karush-Kuhn-Tucker (KKT) conditions are met (Karush, 1939; Kuhn and Tucker, 1951). An exact optimization solution guarantees that no other feasible solution will be a better solution for the specified set of constraints or weights. Unfortunately, it is difficult to predetermine these values because they require forecasting the relative economic values of genetic gains and retention of useful genetic diversity in terms of immediate returns and future benefits. For commercial plant breeding projects, competition in the marketplace will force much greater emphasis on maximizing genetic gains than retaining genetic diversity to maximize immediate return on investment. In contrast, public soybean breeders have an opportunity, perhaps an obligation, to retain useful genetic diversity while realizing genetic gains for quantitative traits of agronomic importance. Since each plant breeding project has unique relative trade-offs, evolutionary algorithms have been adopted to provide multiple solutions on an efficient (Pareto) frontier of solutions to competing objectives (Deb, 2003, 2011; Konak et al., 2006). Decision makers then decide which of the solutions have the appropriate relative emphasis on the competing objectives.

Genetic improvement can be viewed as single or multiple connected search strategies in genotypic space (Podlich and Cooper, 1999; Cooper et al., 2002, 2014). The single search strategy, a.k.a. global, corresponds to the selection of lines in centralized populations, where genotypes from all the sub-populations are treated as one population (Technow et al., 2021). The multiple connected search strategy, a.k.a. local, occasional and corresponds to selection of lines in multiple domains with infrequent exchange of lines. Search strategies in genotypic space inspired the development of a class of evolutionary algorithms known as genetic algorithms (GAs). GAs are based on recurrent selection of breeding populations and are often used to find computational solutions to large combinatorial problems (Goldberg, 1989; Luque, 2011).

In a canonical GA, selected solutions are pooled together into a set of solutions. Subsequently the individual solutions are randomly sampled for pairwise “matings” to create a new set of solutions for evaluation and selection. Computational analogs of mutation or recombination referred to as mutation and recombination operators, are utilized to move the population of solutions into new domains in the solution space towards global optima. The algorithm is iterated until there are no improvements in the sets of solutions. Inspired by Wright’s shifting balance theory of evolution, researchers developed a subclass of GAs, known as parallel Gas, that maintain structure among subsets of individual solutions and enable the subsets to independently find different solutions for different domains (Wright, 1967; Wright, 1988; Cantú-Paz, 2000; Luque, 2011; Yabe et al., 2016). The parallel GA is analogous to the concept of island model selection in genetic subpopulations. The term island refers to distinct sub-populations, where genotypes from any of the sub-populations cannot randomly mate with lines from other sub-populations due to restrictive rules for mating. However, Island Model/Parallel GAs allow for an exchange of solutions among subpopulations that are evolving in parallel. Island model GAs are also distinct from canonical GAs in terms of properties because evolution happens locally, within island, as well as globally, among islands. Island model parameters consist of number of islands, island size, selection pressure within each of the islands, numbers of migrants, migration frequency, connectedness or topology of islands, and emigration and immigration policies among islands (Whitley et al., 1999; Skolicki, 2007; Skolicki and Jong, 2007).

Rather than investigate the trade-off between objective functions, Jannink (2010) demonstrated that it is possible to retain useful genetic diversity in GS by weighting low-frequency alleles with favorable estimated genetic effects. Simulations with Weighted Genomic Selection (WGS) resulted in greater responses across 24 selection cycles of recurrent selection than unweighted GS, using RRBLUP estimated breeding values, for both low and high heritability traits. However, the initial rates of response using WGS were less than responses from the application of PS and less than GS. The response using WGS was better than the response from PS after 20 cycles of selection, but the responses relative to GS depended on the number of simulated QTL and heritability. Decay of linkage disequilibrium (LD) between marker and QTL is one of the factors that can slow responses using GS relative to PS (Hickey et al., 2014; Xavier et al., 2016), although decay of LD did not contribute to responses in the initial cycles using WGS. The rate of inbreeding per cycle is also greater with GS than with PS, whereas it is similar to PS when WGS is applied. The rate of fixation of favorable alleles is lower for WGS than GS resulting in larger numbers of cycles of genetic improvement before response to selection reaches a limit (Jannink, 2010). Efforts to balance the response in early cycles and later cycles have included addition of parameters to WGS (Sun and VanRaden, 2014) and dynamic weighting of rare alleles depending on the time horizon for the breeding program (Liu et al., 2015). Low-frequency favorable alleles are given greater weights, drawn from a beta distribution, in initial cycles, and the weights tend toward unity as the number of cycles of selection approaches a predefined time horizon. This shifts the balance towards retaining greater genetic variance in earlier cycles.

Investigations of GS, WGS, genomic optimum contribution selection, and genomic selection index assume that selected individuals will be randomly mated. Typically, plant breeders do not randomly mate selected genotypes, rather, most use selected genotypes that exhibit the most desirable selection metrics, e.g., GEBVs, to serve as “hub” parents in networked crossing designs (Guo et al., 2013, 2014). Because the metaphor of hubs with spokes represents the preference for crossing most selected lines to a few “hub” lines, we refer to this mating design as a Hub Network (HN), and this is the mating design used in our previous investigation (Ramasubramanian and Beavis, 2020). A Hub Network (HN) mating design applies greater weights to genetic contributions from hub genotypes resulting in amplified loss of genetic diversity relative to Random Mating (RM) by reducing the effective population size.

As soybean breeders have become aware of the potential impacts due to loss of genetic diversity from use of GS, they have used various *ad hoc* methods to avoid crosses between related genotypes (Diers, Graef, Lorenz, Cianzio, Singh, Byrum, Xu personal communications). After quantitative geneticists working on animal breeding systems demonstrated that it is possible to use the genomic selection index strategy with an evolutionary algorithm to identify optimal pairs of mates (Kinghorn, 2011; Pryce et al., 2012; Woolliams et al., 2015), plant quantitative geneticists developed and investigated various versions of genomic selection index and genomic optimum contribution selection for plant breeding (Akdemir and Sánchez, 2016; De Beukelaer et al., 2017; Cowling et al., 2017; Lin et al., 2017; Gorjanc et al., 2018; Allier et al., 2019a, b). Notice that the computational demand to find the optimum on the non-decreasing efficiency frontier created by all possible constraint values or relative weights in all N choose 2 (NC2) mating pairs is particularly well suited for application of GAs. Also, it should be noted that Akdemir and Sánchez (2016) referred to their implementation of genomic optimum contribution selection as efficient GS. In addition to evaluating traditional PS, GS, and genomic optimum contribution selection, Akdemir and Sánchez (2016) proposed and evaluated a novel mathematical programming model, referred to as genomic mating (GM). They formulated the problem as minimizing a linear function of inbreeding plus a negative risk function for the realized relationship matrix of N_*p*_ possible parents. Inbreeding is a function of the expected genetic diversity among N_*c*_ progeny from the N_*p*_ parents and is weighted by a parameter that controls allelic diversity among all N_*p*_ parents. Risk is determined for each cross as the sum of the expected breeding values of the progeny plus the expected standard deviations of marker loci weighted by a parameter that controls the allelic heterozygosity of the relative contributions of the marker loci to the GEBVs. Thus, risk is similar to the usefulness criterion, defined by Schnell (1983) (as cited in Melchinger et al., 1988), of a selected proportion of the population and the weighting parameter reflects the breeders’ emphasis of its importance. They demonstrated that their GM formulation is equivalent to an optimization problem of minimizing inbreeding subject to defined level of risk, denoted ρ. The solution needs to calculate risk and inbreeding for the range of acceptable ρ values for N_*c*_ progeny from N_*p*_ parents, i.e. (Akdemir and Sánchez, 2016) developed a Tabu-search GA to determine the efficiency frontier between inbreeding and risk. In an updated version, Akdemir et al. (2018) used a GA to find the complete set of non-dominated solutions (Deb, 2003, 2011) that comprise the efficiency frontier for the three criteria of Gain (G), Inbreeding (I), and Usefulness (U) values in the objective function. This allows the selection of a subset of solutions for evaluation, obviating the need for conducting a grid search across all possible values. More details on the GM method are provided in Supplementary File 1.

Akdemir and Sánchez (2016) demonstrated the utility of their genomic mating approach using simulations of recurrent selection beginning with two founders for a trait composed of simple additive genetic architecture. The QTL were evenly distributed across a simulated genome consisting of three diploid linkage groups. Their results indicated that the efficiency frontier produced responses across 20 cycles that were better than PS and as good as GS and genomic optimum contribution selection for the first five to seven cycles and better than PS, GS, and genomic optimum contribution selection thereafter (Akdemir and Sánchez, 2016). They did not include WGS for comparison in their study.

Recognizing that Island Model/Parallel GAs are very efficient at finding global optima, Yabe et al. (2016) suggested that computational island models could be used to create efficient and effective breeding plans for plant breeders. Even though computational parallel GAs allow the software developer to change mutation and recombination rates, which are not under the control of plant breeders, structures of breeding populations based on island models could offset the loss of useful genetic variability through regulation of exchange of genotypes among sub-populations. It is not unusual for plant breeders of crops that are easily self-pollinated to routinely evaluate, select, and recurrently cross lines derived from one or two specific bi-parental crosses. In the vernacular of soybean and maize breeders, this is known as “working a population.” Yabe et al. (2016) demonstrated that GS on populations organized as islands provided greater response to selection than GS on a single population comprising all the islands, after the 12th of 20 cycles of recurrent GS. Their founder population consisted of lines derived from *in silico* crosses of six homozygous rice lines with an elite rice variety, i.e., a hub network. They isolated the six families of Recombinant Inbred Lines (RILs) for recurrent selection using GS with no or occasional exchange of selected lines among the family islands. While their results appeared to be similar to WGS, they did not compare their results with WGS. They also suggested that the trade-off between genetic gain and retention of useful genetic variance could be improved by adjusting the number and frequencies of migrants among sub-populations. We hypothesize that a breeding strategy consisting of breeding populations organized as family islands and in which crossing decisions are based on genomic mating will provide small soybean genetic improvement projects with the ability to minimize the trade-offs between maximizing genetic gain and minimizing the loss of useful genetic variability.

Within the populations organized as islands, we evaluated four migration policies, three selection methods, and four mating designs. Given that each of the factors show characteristic average response patterns with widely different rates and limits of responses, we also hypothesize that the combinations of all these factors will further increase the number of possible response curves due to the interaction among these factors. To evaluate the potential of these combinations of methods to realize genetic gains while retaining useful genetic diversity, we compare outcomes from simulated recurrent selection applied to contemporary soybean germplasm adapted to Maturity Group (MG) II and III using a set of metrics (Ramasubramanian and Beavis, 2020), which includes the standardized genotypic value (Rs), the most positive genotypic value (Mgv) among F_5_-derived lines selected in cycle c, the standardized genotypic variance (Sgv), the average expected heterozygosity (Hs), and the lost genetic potential of populations based on the number of favorable alleles that are lost.

## Methods

### Simulations

Initial sets of soybean lines were generated by simulating crosses of 20 contemporary homozygous lines representing the diversity of soybean germplasm adapted to MGs II and III with IA3023, a former widely grown variety adapted to MG III, to generate *in silico* F_1_ progeny (Ramasubramanian and Beavis, 2020). Individual F_1_s from each of the 20 crosses were self-pollinated *in silico* for four generations to generate 100 lines per family forming 2000 lines organized into 20 families with genotypic information at 4289 genetic loci (Song et al., 2017). Thus, the genetic structure of the initial simulated populations is similar to that used in the experimental SoyNAM investigation (Guo et al., 2010; Takuno et al., 2012; Song et al., 2015, 2017; Xavier et al., 2017; Diers et al., 2018).

As reported previously (Ramasubramanian and Beavis, 2020), there were 3818 polymorphic loci in the combined population consisting of 20 families with an average of 773 polymorphic loci within each of the families for the initial founding sets of lines. The variance of the number of polymorphic loci among families was ∼34, which indicates that the number of polymorphic loci is roughly similar among all families. Across the 20 families of Cycle 0 (C0) lines, average expected heterozygosity was 0.09 with an estimated variance of 4.4^∗^10^–7^ among families. The average estimated G_*st*_ value across the genome for the initial founding set of F_5_-derived lines was 0.32, as determined by the “diff_stats” function in the mmod R package (Jombart, 2008; Ryman and Leimar, 2009; Jombart and Ahmed, 2011; Ramasubramanian and Beavis, 2020). Average pairwise “Fst” estimated using “pairwise.fst” in “hierfstat” R package (Goudet, 2005) among the 20 families in simulated data is 0.20. Pairwise “Fst” is a measure of population differentiation among pairs of populations based on Nei’s genetic distance, which is estimated as the ratio of difference between the weighted average of the expected heterozygosity of pairs of families and total expected heterozygosity of the pooled populations to total expected heterozygosity of the pooled populations. For two populations “A” and “B” of size n_*A*_ and n_*B*_, expected heterozygosity (averaged over loci) is denoted as H_*s (A)*_ and H_*s (B)*,_ respectively. Let H_*t*_ denote the expected heterozygosity of a pooled population of “A” and “B.” Then, the pairwise F_*st*_ between “A” and “B” is computed as: $\mathrm{Fst}\left(A,B\right)=\frac{\text{Ht}-\left(\frac{\text{nAH}s\left(A\right)+n\text{BH}s\left(B\right)}{\left(nA+nB\right)}\right)}{\text{Ht}}\;$ (Goudet, 2005). For comparison, the average F_*st*_ using genotypic data from the SoyNAM project among 40 families is 0.09 with a maximum pairwise Fst of 0.15 and a minimum Fst of 0.007 (Ramasubramanian and Beavis, 2020), whereas the average Fst among the clusters in the USDA soybean germplasm collection is 0.23 (Song et al., 2015; Xavier et al., 2018).

### Combinations of Factors

We evaluated 60 combinations of factors (Table 1) that could influence responses to recurrently selected populations derived from a set of founder genomes representing the diversity of contemporary soybean germplasm adapted to MG II and III in North America (Mikel et al., 2010; Diers et al., 2018). The factors included structure of breeding populations, selection method, and mating design. The structure of the breeding populations, which refers to the presence of distinct sub-populations, included retaining the structure of the original 20 founder families through restrictive breeding rules, referred to as family islands, and dissolving family structures after the initial founder population was created, referred to as centralized populations. For comparison with previous studies, the centralized population structure corresponds to the bulked population in Yabe et al. (2016) and the centralized policy in Technow et al. (2021). The family island structure with migration of lines among islands is also called as distributed policy in Technow et al. (2021), whereas islands that are not connected to each other are called as isolated in Technow et al. (2021) and is the policy termed as discrete selection in Yabe et al. (2016).

**TABLE 1:** Treatment design representing the factors that impact responses and limits of responses that were investigated.

Factors	Levels	Values for levels
**Population type**	2	Centralized and Island populations

**Island model selection**		

**Migration frequency**	1	Migration frequency of 2 corresponds to migration of lines every other cycle of selection
**Migration size**	1	Migration of 2 lines per migration event (20%)
**Migration policy**	4	(i) Isolated selection (IS) (For IS, migration frequency, size and direction are set to “0”) (ii) Best island (iii) Random best (iv) Fully connected
**Migration direction**	1	(i) Bi-directional

**Factors common to Non-island and Island populations**		

**Selection method**	3	(i) Phenotypic selection, (ii) Genomic selection, (iii) Weighted genomic selection
**Mating design**	4	(i) Hub network (ii) Chain rule (iii) Random mating (iv) Genomic mating
**Genetic model parameters**	1	400 QTL and 0.7 H
**Total number of combinations of treatment factors**	60	
**Total number of simulations**	**5 (replicates/combination of factors)**	**300**

Previously, we demonstrated that the development of homozygous lines for phenotypic evaluation would limit the numbers of segregating linkage blocks with effective QTL effects. Our evaluations of responses with 40, 400, and 4289 QTL showed that responses for 400 QTL followed a pattern that facilitated the study of the impact of factors on short-term and long-term responses, as the responses realized limits around 15–20 cycles, whereas for 40 and 4289 QTL, the responses reached limits within 10 cycles and around 30 cycles, respectively (Ramasubramanian and Beavis, 2020). Consequently, we chose to designate only 400 polymorphic marker loci as simulated QTL. The QTL were distributed uniformly among the SNP loci and each contributed equal additive effects of ± 0.5 units to the total genotypic value of a line. Thus, cycle C0 lines derived from the founders had an average genotypic value of zero and the potential to create genotypic values ranging from −200 to +200. Phenotypic values were simulated by adding non-genetic values sampled from N (0, σ_*e*_^2^) distribution to the simulated genotypic values, where σ_*e*_^2^ that corresponds to non-genotypic variance was determined by the heritability (σ_*e*_^2^ = ((1−H)/H)^∗^ σ_*g*_^2^), where σ_*g*_^2^ corresponds to genotypic variance and H corresponds to broad sense heritability. Herein we report only simulated broad sense heritability values on an entry mean basis of 0.7. The non-genetic variance was held constant across subsequent cycles of selection. Thus, heritability is expected to decline with every cycle of selection due to loss of additive genetic variance relative to a constant non-genetic variance.

**FIGURE 2:**
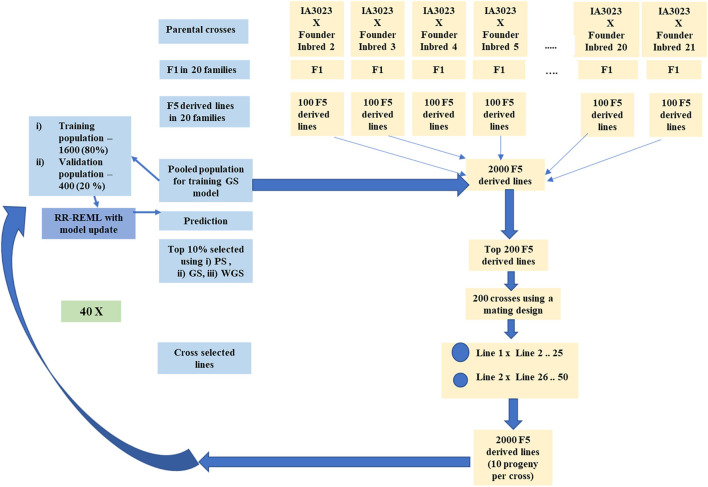
Schematic representing simulated recurrent selection in centralized populations comprising 20 families. The schematic depicts the *in silico* steps used to generate the base population of 2000 F_5_ derived lines derived from 20 founder lines crossed to IA3023. The depiction includes the model training step and the recurrent steps of prediction, sorting, truncation selection, crossing, and generation of 2000 F_5_-derived lines for each cycle as well as the decision steps to check if the training set should be updated and if the recurrent process should be continued for another cycle.

**FIGURE 3:**
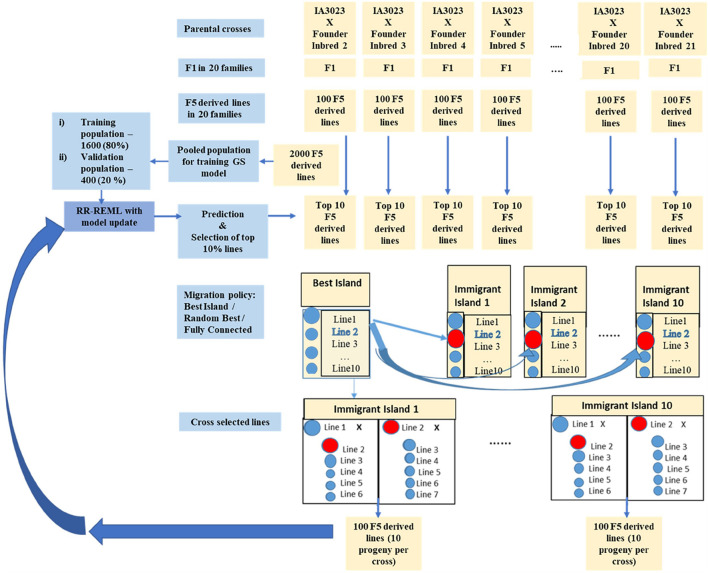
Schematic representing simulated recurrent selection of family island populations where each of the 20 families from the founders is considered an island population. The schematic depicts the *in silico* steps used to generate the base population of 2000 F_5_ derived lines derived from 20 founder lines crossed to IA3023; 100 F_5_-derived lines generated from each of the crosses form a distinct island. The depiction includes the model training step and the recurrent steps of prediction, sorting, truncation selection within islands, migration, crossing, and generation of 100 F_5_-derived lines per island for each cycle as well as the decision steps to check if the training set should be updated and if the recurrent process should be continued for another cycle. The blue shaded circles represent lines that are descendants of the founder populations in the islands and red shaded circles represent lines that are replaced by immigrants from the island with the largest genotypic value for the “Best Island” policy.

Phenotypic selection (PS), genomic selection (GS), and weighted genomic selection (WGS) were applied recurrently to both population structures. Recurrent selection applied to the centralized populations consisted of ranking all lines in a given cycle (Figure 2) according to the selection metric and retaining 10% for crossing to create the next cycle of lines. In terms of standardized selection differential, this corresponds to selection intensity, *ι* = 1.75. For selection of lines organized into family islands, 10% of the lines are selected within islands (Figure 3). Subsequently, 20% of lines might be migrants from other family islands depending on migration rules (Table 1). Metrics used for selection include simulated phenotypic values for PS, genome estimated breeding values (GEBVs) for GS, and weighted genome estimated breeding values for WGS. We used the weighting function used by Jannink (2010) for estimating weighted genome estimated breeding values (Supplementary Table 1). A previous study indicated that among GS methods, Ridge Regression (RR) provided the best compromise between short-term and long-term responses (Ramasubramanian and Beavis, 2020); thus, we only used RR to train GP models for GS. RR was implemented with a method that employs Expectation Maximization to obtain Restricted Maximum Likelihood Estimates of marker effects (Xavier, 2019).

For both GS and WGS, the training models were updated every cycle of selection with data sets from all prior cycles. Since average within-family prediction accuracies are less than prediction accuracies from combined training sets comprising F_5_-derived lines from across all the families (Ramasubramanian and Beavis, 2020), we used a training set comprising F_5_-derived lines from all the families. Training sets for each cycle were obtained by randomly sampling 1600 lines from the set of 2000 lines in each cycle. The most accurate predictions and maximum genetic responses were obtained with training data that are cumulatively added every cycle. For purposes of this manuscript, model updating refers to retraining the model with data from the current cycle as well as all prior cycles that were cumulatively added.

Subsequent to selection, four mating designs were applied to create the next cycle of lines (Table 1). To simulate theoretical truncation selection, selected lines were randomly mated (RM). The chain rule (CR), a.k.a., a single round-robin mating design (Yabe et al., 2016), is an alternative to RM that assures all selected lines contribute to the subsequent cycle of evaluation and selection. In contrast to the attempt to assure equal representation of selected lines through RM and CR, most soybean breeders use a mating design that assures most progeny will be derived from crosses of a few lines that exhibit the most desirable performance (Guo et al., 2013, 2014). In the hub network (HN) mating design, the hub parents with the largest selection metrics make the largest parental contributions (Ramasubramanian and Beavis, 2020). The fourth mating design, genomic mating (GM), uses mathematical objective functions to assure that defined breeding objectives are used to identify pairs of crosses from among the selected lines. GM method was implemented using the “Genomic Mating” R package (Akdemir et al., 2018). As originally described, GM combines selection and mating in a single step, but we decomposed the steps to provide comparable outcomes from all other combinations of selection methods, mating designs, and organized populations.

#### Genomic Mating in Centralized Families

In a selected set of 200 lines, there are 200C2 (19900) combinations of parental pairs. To solve the objective function w.r.t., an initial population of parental pairs, 250 initial populations of 200 combinations of parental pairs, is sampled from 19900 combinations (19900C200) for the genetic algorithm to solve.

#### Genomic Mating in Populations Organized as Family Islands

In island selection, ten lines are selected from each of the 20 family islands. Within each island, 45 (10C2) combinations of parental pairs are possible (Supplementary Figure 1). To solve the objective function w.r.t., an initial population of parental pairs, 250 initial populations of 10 combinations of parental pairs, is sampled with replacement to keep the population size equal to the centralized populations for the GA. For each of the 20 families, the GA is applied to the initial subset of 250 out of all possible combinations (45C10). The other parameters for the GA are the same for both centralized and family island populations. The genetic algorithm selects non-dominated elite solutions (Deb, 2003, 2011) and crosses non-dominated elite solutions for 50 iterations with a mutation probability of 0.8 (Supplementary Figure 1). Examples of pseudocode are provided in Akdemir and Sánchez (2016) and the “Genomic Mating”. It is important to note that the parameter values in the genetic algorithm can be optimized, and the set of solutions in the pareto-front can be explored for better solutions using other methods such as NSGA-II, NSGA-III, SPEA-1, SPEA-2 and other recent improved versions of GAs for better convergence rate and quality of solutions, determined by the proximity to global optimum (Deb, 2011; Seada and Deb, 2018; Supplementary Figure 1).

### Migration Rules Among Family Islands

In addition to applying selection methods and mating designs to both population structures, there are many possible rules that affect migration among islands. Migration rules that were implemented in a preliminary investigation included: (1) frequency of migration—never, once every two cycles, and every cycle of recurrent selection; (2) the proportion (10% and 20%) of immigrants that will be included in crosses responsible for creating the next cycle of lines; (3) migration can be either in one direction or it can be reciprocal among family islands. Based on the preliminary investigation (results available on request), we decided to set the migration rule as bi-directional migration between both immigrant and emigrant islands of two lines once every other cycle of selection.

#### Migration Policies Among Family Islands

Migration policy (MP) refers to the nature of island topology specifying connections between emigrant and immigrant islands. The four levels for migration policy included “Isolated” (IS), “Best Island” (BI), “Random Best” (RB), and “Fully Connected” (FC). For the BI policy, emigrant lines are selected from the island with most desirable average genotypic value in the islands, and selected lines can migrate to no more than 10 islands. Given a bi-directional migration rule, the emigrant island also receives two immigrants from the islands that received the emigrants. For an RB policy, an emigrant island is selected randomly from a set of 10 islands with high average genotypic values, while the migration pattern itself is similar to BI policy. For the FC policy, every island is connected to every other island, and lines migrate from emigrant islands with high values to randomly selected immigrant islands (Supplementary Figure 2).

Note that migration factors are irrelevant for populations that did not maintain the structure of family islands, and they are irrelevant for isolated family islands where there is no migration. Thus, the treatment design is not a complete factorial, rather, the complete set is comprised of responses for 60 combinations of factors with five independent replicates per combination of factors. The parameter values for levels of island selection specific factors were selected based on limits of responses from a larger set of simulations (2664 combinations of factors with 10 replicates per combination of factor) performed for a preliminary study. The migration rules investigated included migration of one or two lines every cycle, or every other cycle or once in three cycles in one or both directions. Mating designs included (HN, CR, and RM) for 40, 400 and 4289 QTL with 0.7 and 0.3 H (response patterns for this set are available on request).

### Modeled Response to Recurrent Selection

The averaged genotypic value for each cycle, c, of recurrent selection was modeled with a linear first-order recurrence equation:


(1) $$f_{0}\left(c\right)y_{\left(c+1\right)}+f_{1}\left(c\right)y_{\left(c\right)}=g\left(c\right)$$

where c is a sequence of integers from 0 to 39 representing each cycle of recurrent selection from cycle 1 to 40 and *f*_0_, *f*_1_, and *g* are constant functions of c. By rearranging the equation, we note that the response in cycle c+1 can be represented as


(2) $$y_{\left(c+1\right)}=-\frac{f_{1}\left(c\right)}{f_{0}\left(c\right)}y_{\left(c\right)}+\frac{g\left(c\right)}{f_{0}\left(c\right)}$$

Since the ratios *f*_1_(c)/*f*_0_(c) and *g*(c)/*f*_0_(c) are constants, we can represent the response in cycle c+1 as


(3) $$y_{\left(c+1\right)}=\alpha y_{\left(c\right)}+\beta$$

If *y*_0_ specifies the average genotypic value of the first cycle of F_5_ lines derived from crosses involving IA3023 and the other founders, then (3) has a unique solution (Goldberg, 1958; Ramasubramanian and Beavis, 2020):


(4) $$\begin{array}{c} y_{c}=\alpha^{c}y_{0}+\beta \frac{1-\alpha^{c}}{1-\alpha }\text{if}\alpha \neq 1\\ {\text{y}}_{c}=\alpha^{c}{\text{y}}_{0}+\beta c\;\text{if}\alpha =1 \end{array}$$

An alternative representation of (eqn 4) for the situation of α ≠ 1 is


(5) $$\begin{array}{c} y_{c}=\alpha^{c}\left(y_{0}-y^{\prime }\right)+y^{\prime }\\ \mathrm{with}y^{\prime }=\frac{\beta }{1-\alpha } \end{array}$$

where α is less than 1 for genotypic response to recurrent selection and *y*′ represents the asymptotic limit to selection (Goldberg, 1958; Ramasubramanian and Beavis, 2020). An illustration of the values of the sequence of c = 0–39 for a range of α and β values can be found in our previous study (Ramasubramanian and Beavis, 2020). The model-derived curves can be interpreted as response to selection as a function of the frequencies of alleles with additive selective advantage, selection intensity, time, and effective population size (Robertson, 1960). The parameters, α, and β were estimated with a non-linear mixed effects method implemented in the “nlme” and “nlshelper” packages (Pinheiro and Bates, 2000; Baty et al., 2015; Pinheiro et al., 2021).

Since the limits of responses are approached asymptotically, the number of cycles required to reach half of the limits before there is no longer response to selection is referred to as the half-life of the recurrent selection process (Robertson, 1960; Dempfle, 1974; Cockerham and Burrows, 1980; Kang and Namkoong, 1980; Kang, 1983; Kang and Nienstaedt, 1987). From the first-order recurrence equation (5), the half-life is estimated as


(6) $$t_{1/2}=\ln\left(0.5\right)/\ln\left(\alpha \right)$$

when y_0_ is “0” and the asymptotic limit is estimated as y′ (Ramasubramanian and Beavis, 2020).

### Analyses of Variance (ANOVA) of Modeled Response to Recurrent Selection

Analyses of variance is used to evaluate the impact of factors and their interactions on the modeled responses to global and island recurrent selection. The analyses of variance used single-level nlme models with modeled (Eqn 5) responses grouped by combinations of treatment factors. We analyzed the variance among modeled responses using AIC, BIC, and Likelihood metrics that were grouped based on combinations of treatment variables consisting of population type, selection method, mating design, and migration policy for migration frequency, migration size, and migration direction for one genetic model consisting of 400 simulated QTL responsible for 0.7 H with equal additive effects (Table 1). For a discussion of the analyses of variance using non-linear mixed effects models refer to (Pinheiro et al., 2000; Zuur et al., 2009; Baty et al., 2015; Pinheiro et al., 2021; Oddi et al., 2019; Ramasubramanian and Beavis, 2020).

In the first phase of model fitting, we fit a random intercept model for estimating both α and β in the recurrence equation using the “nlme” R package. Estimates of modeled parameters from nlsList models were retained as starting values for fixed effects. Multiple ANOVA of “nlme” objects representing the models were used to identify combinations of factors with significant impacts on the non-linear response. The model with the lowest AIC score was selected as the best model. The best random intercept model in the first phase of model fitting process M15 and models with combinations of three factors (M11-M14) showed evidence of auto-correlation among residuals. Since auto-correlation violates the independence assumption, the correlation among residuals was modeled using AR-1 correlation structure. Since the genotypic values across cycles in recurrent selection are correlated, fitting AR-1 correlation does not remove the correlation unless cycles are used as co-variates. However, using cycles as a co-variate makes the model fitting process very time-consuming and often has larger AIC scores than models without cycles as covariates. The Model M15 with AR-1 correlation structure was further refined by modeling variance components using “varIdent” structure in “nlme.” The process for fitting, selecting, and refining mixed-effects models is similar to our previous study (Ramasubramanian and Beavis, 2020) and is described in Supplementary File 2.

### Evaluations of Responses to Recurrent Selection

**FIGURE 4:**
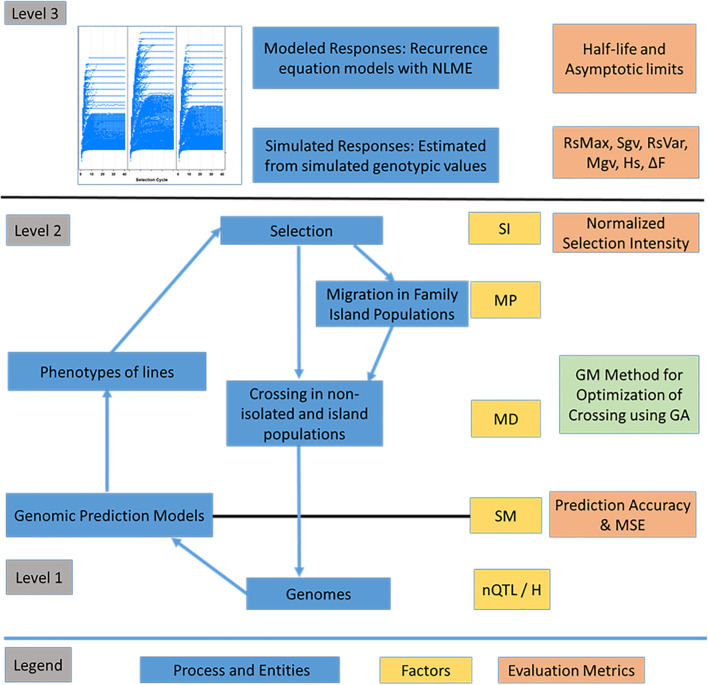
Overview of the recurrent selection process. Representation of entities such as genomes, associated F_5_-derived lines and processes such as the estimation of marker effects, selection, migration and crossing. Levels correspond to layers of information with level 1 comprising genomic information, level 2 comprising phenotypes of lines within and across family islands, and level 3 comprising higher-level information including responses across cycles of selection. The factors include nQTL and H at the genome level, selection method (SM) including phenotypic selection (PS), genomic selection (GS), and weighted genomic selection (WGS). The factors at level 2 include selection intensity (SI - top 10% selected fraction); Mating Design (MD), which includes Hub Network, Chain Rule, Random Mating, and Genomic Mating; Migration Policy (MP), which includes: “Isolated Selection,” “Best Island,” “Random Best,” and “Fully Connected” policies. Among the MD levels, GM method involves application of evolutionary multi-objective optimization to minimize inbreeding and maximize gain and usefulness. Level 3 is characterized using evaluation metrics such as half-life and asymptotic limits derived from recurrence equation models and metrics such as Standardized Responses (Rs), Standardized genotypic variance (Sgv), Maximal genotypic values (Mgv), and efficiency of converting loss in genetic variance into gain (RsVar) derived from simulated outcomes. Other metrics include prediction accuracy and MSE for GP models (RR-REML) and expected heterozygosity (Hs).

Evaluations of responses to recurrent selection were conducted on both modeled and genotypic values using a set of metrics described in Ramasubramanian and Beavis (2020) and defined below. The estimated population half-life and asymptotic limits used the estimated parameters α and β of the first-order recurrence model. The average genotypic values were used to estimate the standardized genotypic value (Rs) and maximal genotypic value (Mgv). Maximum possible genotypic potential of the founders provided a reference for number of favorable alleles retained in the population. The loss of genotypic potential is characterized by reduction in the standardized variance of genotypic values (Sgv) and estimated heterozygosity (Hs). In addition, efficiency of conversion of loss in genotypic variance into genetic gain (Rs_var) provides a way to assess gain in genotypic value and loss of genetic variance simultaneously. For selection using island models, the different impacts of selection strategies on the genotypic variance at individual island or global levels are assessed using intra-island Sgv, inter-island, and global variance of genotypic values. A schematic diagram of the processes, factors, and evaluation metrics used to characterize the responses to recurrent selection is provided in Figure 4.

#### Evaluation Metrics

The standardized genotypic value, R_*s*_ was estimated in every cycle of selection as the proportion of maximum genotypic potential (200 units) relative to the average genotypic value of 2000 lines in C0 (Eqn 7). Values range from 0 to 1 with the value of 1 corresponding to the maximum possible genotypic value with the genetic model and 0 corresponding to the average genotypic value of C_0_ (Meuwissen et al., 2001; Liu et al., 2015; Ramasubramanian and Beavis, 2020).


(7) Rs=Rc(R-mR)0

R_*s*_ - Standardized genotypic value

R_0_ - Average genotypic value of F5 derived lines produced by founders

R_*c*_ - Average genotypic value in cycle ‘c’ – R_0_

R_*m*_ - Maximum possible genotypic value (200)

Since we previously evaluated the genetic improvement of soybean using PS and the HN mating design in centralized populations, we used PS with a selection intensity of 1.75 for the centralized population and HN mating design (designated as CE-PS-HN) as a reference for comparing novel combinations of selection and mating designs proposed in the study. A standardized relative genotypic response, ΔRs_*c*_, is calculated in equation (8) as the percentage of the difference in standardized genotypic values, Rs_*c*_, in each cycle c.


(8) $$\mathrm{Percent}\mathrm{Gain}\mathrm{in}{\mathrm{Rs}}_{\text{c}\left(\mathrm{Design}-x\right)}=\frac{{\text{Rs}}_{\text{c}\left(\mathrm{Design}-x\right)}-{\mathrm{Rs}}_{\text{c}\left(\mathrm{CE}-\mathrm{PS}-\mathrm{HN}\right)}}{{\text{Rs}}_{\text{c}\left(\mathrm{CE}-\mathrm{PS}-\mathrm{HN}\right)}}*100$$

Rs_c(Design–X)_ - standardized response for Design-x in cycle ‘c’

Rs_c(CE–PS–HN)_ - standardized ersponse for CE-PS-HN design in cycle ‘c’

The standardized genotypic variance (*Sgv*), defined as the change in estimated genotypic variance from the estimated genotypic variance of the initial population of lines from C0, was used to evaluate the changes in estimated genotypic variance across cycles of recurrent selection. Note that values for *Sgv* range from zero to one as it is standardized to the maximum genotypic variance among founders.

Efficiency of genetic improvement is a metric used to evaluate the proportion of genetic improvement that was obtained through loss of genetic diversity from recurrent selection (Gorjanc et al., 2018). Efficiency is estimated as the slope in linear regression in linear regions of response curves. However, responses to recurrent selection in the absence of mutation are inherently non-linear (Robertson, 1960; Bulmer, 1971; Hill and Robertson, 2008; Ramasubramanian and Beavis, 2020). For purposes of evaluating the relative contribution of lost genetic variance to genetic response in both linear and non-linear segments of the response curve, we introduce the standardized genotypic variance of the response, Rs_Var, calculated with Equation (9).


(9) $$\mathrm{Rs}\_\mathrm{var}=\frac{{\text{G}}_{\text{c}}-G_{0}}{{\text{SdG}}_{0}-{\mathrm{SdG}}_{\text{c}}}$$

G_c_ -average genotypic value of the set of F5 derived lines evaluated in cycle ‘c’

G_0_ -average genotypic value of the founding set of F5 derived lines

SdG_0_ - estimated standard deviation of genotypic values of founding set of F5 derived lines

SdG_c_ - estimated standard deviation of genotypic values of F5 derived lines in cycle ‘c’ The numerator term represents the difference in average genotypic values of a population in cycle “c” from cycle “0” normalized to standard deviation of genotypic values in cycle “0.” The denominator represents the difference of standard deviation of genotypic values between cycles “0” and “c” normalized to the standard deviation of genotypic values in cycle “0” (Ramasubramanian and Beavis, 2020). For the centralized populations, Rs_Var was estimated by calculating the variance of simulated genotypic values. Standardizing the estimated genotypic variance with respect to the maximum genotypic values in the initial population results in values that range from 0 to 1. For the family island populations, the genotypic variances can be split into within- and between-island genotypic variance. The three measures we used to estimate the global diversity of populations, inter-island diversity, and within-island diversity are provided in the documentation of the R package.

## Results

### Analysis of Variance of Modeled Genotypic Values

There is strong evidence from the analyses of variance (Supplementary File 4) that the modeled genotypic values across cycles of selection depend on interactions among selection method, mating design, and migration policy. The most parsimonious model included all combinations of factors indicating that interactions among all factors have statistically significant influences on recurrent responses to selection and requires unique estimates of α, and β in (3) for each of the combinations of factors (M15 in Supplementary File 4). For all combinations of factors, we report only migration involving bi-directional migration of two migrants every other cycle. Among the factors that affect only family island populations with migration, migration frequency had significant effects on rate and the asymptotic limits for response to selection, whereas migration direction and size had relatively small effects on rates and no significant effect on the asymptotic limits for response to selection. Rates and genotypic values at the limits of response for a given selection method and mating design also depend on genetic architecture and heritability (data available on request). Rather than belabor the specific outcomes from all possible combinations of factors that affected the modeled responses, the remainder of the reported results are restricted to results from simulations with 400 QTL responsible for 70% of phenotypic variability.

### Rates and Limits of Responses to Recurrent Selection

Factors common to centralized and family island populations such as mating design and selection method as well as factors specific to isolated and island model selection had significant effect on estimated population half-lives and asymptotic limits. Half-lives for selection methods on centralized populations ranged from 3.83 to 16.10 cycles with a mean of 9.62 cycles, and asymptotic limits ranged from 71.64 to 160.76 with a mean of 115.97 (58% of the maximum possible potential in the founders). Compared to centralized populations, half-lives for selection on isolated family islands were very low ranging from 1.97 to 2.89 cycles with a mean of 2.43 cycles, and asymptotic limits ranged from 28.42 to 38.30 and a mean of 33.12 (16.5% of the maximum possible potential in the founders) (Supplementary File 5; Supplementary Figure 3). Estimated half-lives for island model selection methods were on the average greater than selection methods applied to centralized populations ranging from 4.24 to 32.04 cycles with a mean of 13.45 cycles. Asymptotic limits ranged from 47.54 to 198.82 with a mean of 116.8 (58.5% of the maximum possible potential in the founders) (Supplementary File 5; Supplementary Figure 3).

### Responses to Recurrent Selection of Non-island Lines

**FIGURE 5:**
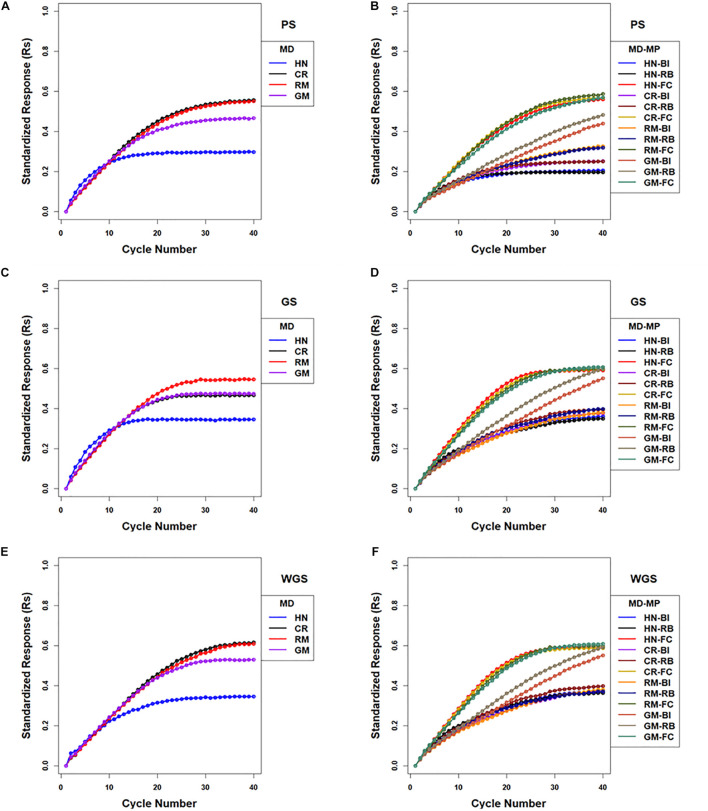
Standardized genotypic responses (Rs) across 40 cycles of recurrent selection on centralized **(A,C,E)** and family island **(B,D,F)** populations, using Phenotypic Selection (PS) **(A,B)**, Genomic Selection (GS) **(C,D)** and Weighted Genomic Selection (WGS) for the four mating designs (MD): Hub Network (HN), Chain Rule (CR), Random Mating (RM), and Genomic Mating (GM). Standardized genotypic responses are represented from a simulated genetic architecture consisting of 400 additive QTL uniformly distributed throughout the genome and responsible for 70% of phenotypic variability. Ten percent of lines are selected to be used in crosses in HN, CR, RM, and GM designs. Migration policies (MP) included the Best Island (BI), Random Best (RB), and Fully Connected (FC) with bi-directional migrations of two migrants every other cycle. GP models are updated every cycle in GS and WGS using training sets with data from all prior cycles of selection.

There were 12 combinations of selection methods and mating designs that were applied to lines in centralized populations. The greatest genotypic values (Rs) were attained with WGS (Figure 5 and Supplementary Table 2). Genomic selection using RRBLUP estimated phenotypic values resulted in greater responses than PS in early cycles while WGS produced greater responses than PS in later cycles (Figure 5; Supplementary Table 2). Weighted genomic selection followed by the CR mating design resulted in the greatest realization of genetic potential before reaching a limit. Genomic selection using RRBLUP estimated phenotypic values followed by an HN mating design resulted in the greatest rates of response in the first ten cycles and, if followed by RM, provided the greatest responses in the first 20 cycles. When the GM design is applied to selected lines to obtain specified crosses according to optimization criteria, the responses in the first 15 cycles were larger than obtained with RM, whereas responses after the 20th cycle were less than responses for other mating designs (Figure 5 and Supplementary Table 2).

The responses measured as maximum genotypic values (Mgvs) produced response patterns similar to Rs. Use of WGS followed by the CR mating design resulted in an average Mgv of 125 (62.5% of the maximum potential in the founders) followed by PS and GS using RRBLUP estimated phenotypic values in the 40th cycle. Genomic selection followed by the HN mating design (CE-GS-HN) realized greater Mgvs relative to other combinations of factors only in the early cycles (Supplementary Figure 4).

**FIGURE 6:**
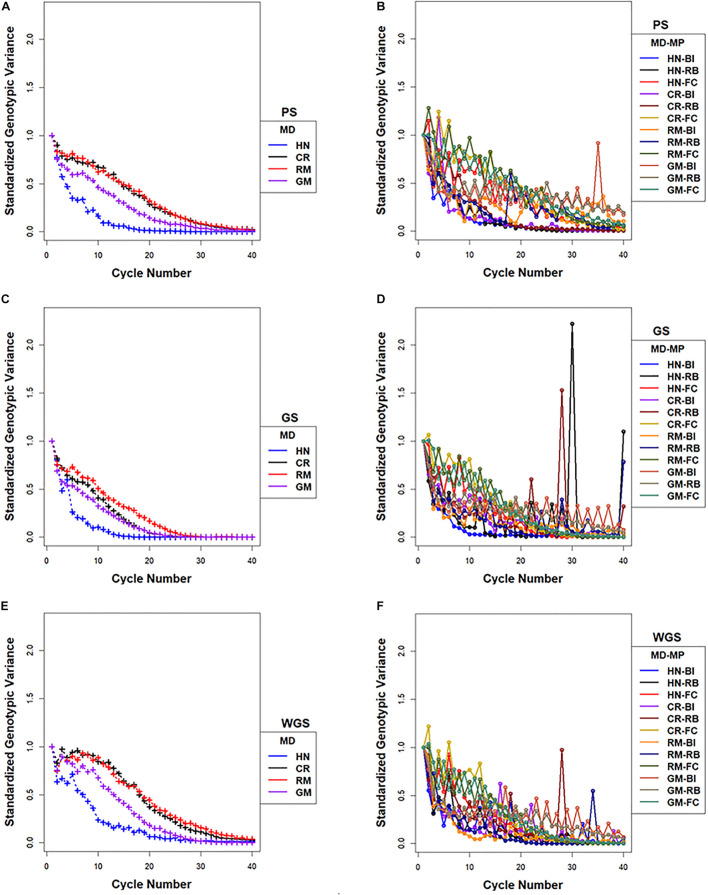
Standardized genotypic variance across 40 cycles of recurrent selection on centralized **(A,C,E)** and family island **(B,D,F)** populations, using Phenotypic Selection (PS) **(A,B)**, Genomic Selection (GS) **(C,D)**, and Weighted Genomic Selection (WGS) **(E,F)** for the four mating designs (MD): Hub Network (HN), Chain Rule (CR), Random Mating (RM), and Genomic Mating (GM). Ten percent of lines are selected for crossing. The genetic architecture in the initial simulated founder lines consisted of 400 additive QTL uniformly distributed throughout the genome and expressed broad sense heritability on an entry mean basis of 0.7. Genetic variance is standardized to the average genotypic variance in founder populations in cycle “0.” Average island genetic variance refers to genetic variance within families averaged across 20 families. Migration policy in the island models included “Best Island” (BI), “Random Best” (RB), and “Fully Connected” (FC) with bidirectional exchange of two immigrants and emigrants every other cycle of selection. GP models are updated every cycle in GS and WGS using training sets with data from all prior cycles of selection.

The rates at which standardized genotypic variance (Sgv) and expected heterozygosity (Hs) decreased depended on the mating designs (Figure 6 and Supplementary Figure 5). The application of RM and CR mating designs after selection helped maintain genotypic variance and heterozygosity for use in later cycles of recurrent selection. The HN mating design resulted in the fastest loss of Sgv and Hs (heterozygosity), while the GM design demonstrated losses of Sgv and heterozygosity that were intermediate between HN and RM/CR designs.

**FIGURE 7:**
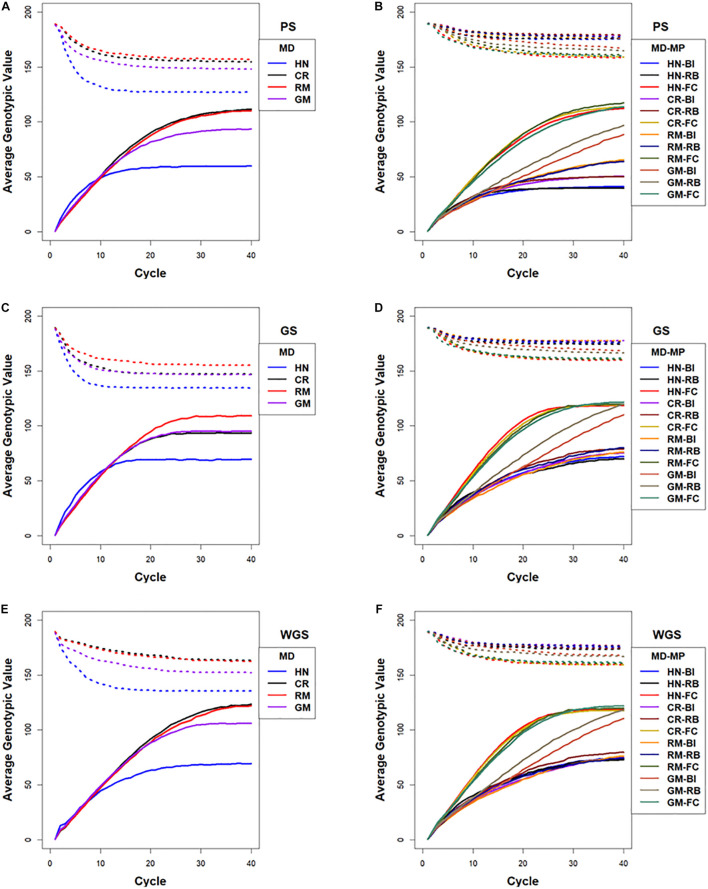
Lost genotypic potential and average genotypic values across 40 cycles of recurrent selection on centralized **(A,C,E)** and family island **(B,D,F)** populations, using Phenotypic Selection (PS) **(A,B)**, Genomic Selection (GS) **(C,D)** and Weighted Genomic Selection (WGS) **(E,F)** and four mating designs (MD): Hub Network (HN), Chain Rule (CR), Random Mating (RM), and Genomic Mating (GM). Ten percent of lines are selected for crosses using HN, CR, RM, and GM mating designs. The genetic architecture in the initial simulated founder lines consisted of 400 additive QTL uniformly distributed throughout the genome and expressed broad sense heritability on an entry mean basis of 0.7. The dotted lines represent maximum genetic potential estimated from favorable alleles that are lost from the population, and solid lines represent increase in average genotypic value of populations due to recurrent selection. Migration policies (MP) in the island models included “Best Island” (BI), “Random Best” (RB), and “Fully Connected” (FC) with bidirectional exchange of two immigrants and emigrants every other cycle of selection. GP models are updated every cycle in GS and WGS using training sets with data from all prior cycles of selection.

The rate at which maximum genotypic potential decreased across cycles of selection was reflected in the estimated number of lost favorable alleles. Among the selection methods, GS using RRBLUP-estimated phenotypic values lost genetic potential faster than PS and WGS (Figure 7). Among the mating designs, HN resulted in the fastest loss of genetic potential while RM lost genetic potential slower than any of the other mating designs. With the GM method, genetic potential was lost at a rate that was intermediate between RM and HN mating designs. The CR design lost favorable alleles at rates that were similar to GM with GS, whereas after applying CR with PS and WGS, the loss of alleles was similar to RM (Figure 7).

Rates of inbreeding are larger for GS compared to PS and WGS in the first 10–15 cycles. The RM and CR mating designs demonstrated the slowest rates of inbreeding, whereas rates of inbreeding with the GM and HN mating had high rates of inbreeding before responses to selection became limited (Supplementary Figures 6, 7). The estimates of genotypic responses, standardized to genotypic variance (Rs_Var), were the greatest in the first 20–30 cycles with CR, RM, and GM mating designs, while the HN mating design lost the greatest amount of Rs_Var with GS, PS, and WGS (Supplementary Figures 8, 9).

### Responses to Recurrent Selection of Lines Organized as Family Islands

The genotypic values when the isolated family island populations reached the limits were as much as 67% less than the values when limits were reached in the centralized counterpart populations (Supplementary Figure 10). Among the isolated selection methods, GS and WGS with GM design (designated IS-GS-GM and IS-WGS-GM) provided the greatest genotypic values at the response limits. Between 10% and 15% of the maximum potential in the founder populations was realized within the first 10 to 15 cycles, when there was no migration among islands (Supplementary Figure 10). Maximal genotypic values (Mgvs) followed a pattern similar to Rs, and Sgvs mirrored the response pattern in IS (Supplementary Figure 10).

In contrast to selection on isolated family islands, genotypic values at the limits were larger using BI, RB, and FC migration policies among islands, where there is exchange of lines. Among the selection methods applied to the family island populations, GS and WGS realized the greatest genetic potential before reaching limits of responses (Figures 5, 7). The impacts of mating designs on the responses to selection applied to family island populations are distinct from those on centralized populations. In the centralized populations, RM and CR mating designs provided the greatest genotypic values before response to selection became limited, whereas in the family island populations, GM provided the greatest genotypic values when coupled with BI and RB migration policies. The FC migration policy with the largest migration rates produced responses that were similar among the HN, CR, RM, and GM designs (Figures 5, 7).

As noted above, the best responses to selection in the first 10 to 20 cycles on centralized populations were obtained using GS followed with an HN or GM mating design (respectively designated CE-GS-HN and CE-GS-GM in Figure 5). The greatest short-term responses to selection in family island populations were obtained using either GS or WGS followed by the HN mating design coupled to a FC migration policy (IM-GS-HN-FC and IM-WGS-HN-FC in Figure 5 and Supplementary Table 2). The gains in the first 10–20 cycles that were obtained using GS and WGS followed by the GM design coupled to a FC migration policy were comparable and showed little difference.

Given the FC migration policy, the largest standardized genotypic responses at the limits to response (0.59–0.61) were obtained using GS or WGS with HN, CR, RM, and GM designs, whereas with RB migration policy, GS and WGS followed by the GM design produced the greatest realization of genetic potential before the 40th cycle (0.59–0.6) compared to (0.3–0.4) with HN, CR, and RM designs (Figure 5 and Supplementary Table 2). The BI policy showed a pattern similar to that of RB, but at a slower rate of response (Figure 5 and Supplementary Table 2).

Maximum genotypic values followed a pattern similar to Rs for most of the island selection methods. In contrast to selection in centralized populations where PS and WGS resulted in the greatest Mgvs in 20–40 cycles, GS in family island populations resulted in larger Mgvs (124.6) than island PS (119.9) by the 40th cycle.

Rates of decrease in maximum available potential are influenced by factors such as selection intensity, selection method, and mating design. Relative to centralized populations, island selection retains allelic diversity in the combined population as selection depletes variance only within islands and not across islands (Figure 7). Such loss in maximum potential is not always reflected in rates of responses. Relaxed selection intensity will result in retention of genetic variance with no significant increase in response as it is observed with BI and RB migration policies when combined with RM designs for PS, GS, and WGS.

Island selection with GM design and FC migration policy showed the least rate of decrease of Hs values for PS, GS, and WGS reflecting a greater potential retained in the population followed by island selection with GM design and RB migration policy (IM-GM-RB) as well as island selection with GM design and BI migration policy (IM-GM-BI). Island selection with HN design and BI policy (IM-HN-BI) as well as RB policy (IM-HN-RB) showed the most rapid decrease in Hs across 40 cycles of selection, whereas CR and RM designs with the same RB and BI migration policies showed intermediate rates of decrease in Hs. There is an oscillatory pattern in the decrease of Hs, where Hs increased with every migration event in early cycles. In late cycles, the magnitude of increase in Hs due to a migration event decreased and the oscillatory pattern dampened to a continuous decrease as the populations approached the limits of responses (Supplementary Figure 5).

Island PS demonstrated lesser rates of inbreeding compared to island GS and WGS. RM design showed the least rates of inbreeding among the four mating designs for BI, RB, and FC migration policies (Supplementary Figures 6, 7). CR design followed a pattern similar to HN or GM depending on the selection method. Among migration policies, the FC policy demonstrated lesser rates of inbreeding compared to BI and RB policies, whereas the BI policy demonstrated the largest rates of inbreeding. The GM design demonstrated rates of inbreeding that were intermediate between RM and HN/CR designs (Supplementary Figure 7).

Rs_Var for island selection with FC migration policies was larger than that observed with centralized populations, demonstrating larger efficiency of converting loss of genetic variance into gain. However, with FC policy, all mating designs showed a similar pattern (Supplementary Figure 8), whereas Rs_Var for island selection with BI and RB policies was comparable to that of centralized PS and GS, except for GM design, which showed larger Rs_Var after 10–20 cycles of selection (Supplementary Figure 9).

#### Diversity Within and Among Islands

The average within-island genotypic variance decreased towards zero through 40 cycles of selection, whereas global and inter-island genotypic variance increased before becoming limited. The rates of decrease in average within-island genotypic variance were influenced by the selection method, mating design, and migration policy. Both GS and WGS demonstrated similar patterns of loss of genotypic variance within islands, and rates of loss with both the selection methods were faster than PS (Figure 6). The HN mating design demonstrated the fastest loss of within-island genotypic variance followed by RM, CR, and GM designs. The FC migration policy provided the slowest loss of within-island genotypic variance followed by RB and BI migration policies (Figure 6). Notice, however, an oscillatory pattern in which within-island genotypic variance increased with every migration event and decreased because of selection in cycles when there were no migrants. For both the within-island genotypic variance and the expected heterozygosity, the magnitudes of oscillations dampened towards zero after 20–30 cycles of selection except for the GM mating designs coupled with BI and RB migration policies (designated IM-GM-BI and IM-GM-RB, respectively, in Figure 6). The amplitude of increased genetic variance due to migration was greater for RB and BI migration policies with large spikes after 25–30 cycles of selection, while the amplitudes were smaller with the FC migration policies (Figure 6).

**FIGURE 8:**
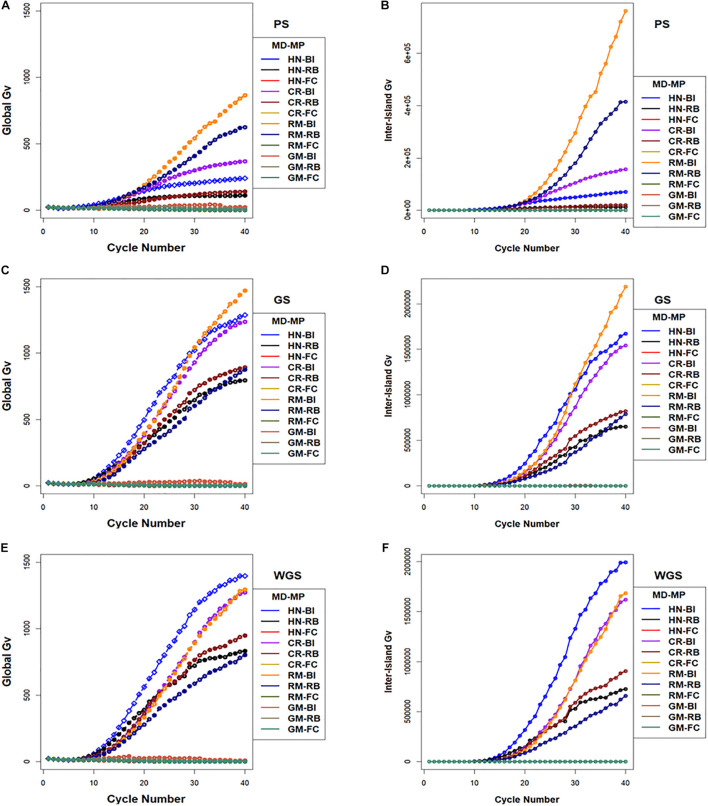
Global and inter-island genotypic variance in island selection. (i) Global genotypic variance **(A,C,E)** and (ii) Inter-island genetic variance **(B,D,F)** for Phenotypic Selection (PS) **(A,B)**, Genomic Selection (GS) **(C,D)**, and Weighted Genomic Selection (WGS) **(E,F)** for the four mating designs including Hub Network (HN), Chain Rule (CR), Random Mating (RM), and Genomic Mating (GM) methods. Migration policy included “Best Island” (BI), “Random Best” (RB), and “Fully Connected” (FC) for 400 simulated QTL and 0.7 H. Migration rules included bidirectional exchange of two immigrants and emigrants every other cycle of selection. Genotypic variance is standardized to the average genotypic variance in founder populations in cycle “0.” GP models are updated every cycle in GS and WGS using training sets with data from all prior cycles of selection.

The largest values for inter-island genotypic variance were obtained with the RM design combined with BI and RB migration policies followed by CR and HN designs with BI and RB migration policies (Figure 8). Whereas, the FC migration policies demonstrated the smallest increases in inter-island genotypic variance through 40 cycles of selection (Figure 8). Recall that the FC migration policies provide the greatest migration rates among islands. Global genotypic variance in family island populations increased due to increase in inter-island genotypic variance. The BI migration policies demonstrated the largest global genetic variance for RM, HN, and CR mating designs followed by the RB migration policies. The GM design with BI and RB migration policies provided intermediate rates of increase in global genotypic variance while the FC migration policy showed the least increase in global genotypic variance when coupled with the HN, CR, RM, and GM mating designs (Figure 8).

Within the classes of migration policies, the migration frequency had significant influence on rates and limits of responses across most combinations of selection methods, mating designs, and migration policies, while numbers of migrants significantly affected responses only for a few combinations of factors. Both rates and limits of response decreased with fewer migrants for the HN mating design. For the RM design, exchange of migrants among family islands once in every three cycles provided the greatest genotypic values at the limits compared to responses with more frequent exchange. Migration size and migration direction had no significant effect on limits of selection responses (data available on request).

### Trade-Offs Between Short-Term and Long-Term Gains From Recurrent Selection

There were 12 combinations of selection methods and mating designs applied to centralized populations and 48 combinations of selection methods, mating designs, and migration policies applied to family island populations. From among the 60 methods, GS using a ridge regression model followed by a hub network mating design in centralized populations and WGS followed by crosses using the CR in the centralized populations respectively (designated CE-GS-HN and CE-WGS-CR in Table 2 and Figure 4) demonstrated the greatest responses in the first 20 and last 20 cycles, respectively. However, if the objective for genetic improvement is to maximize gain in the first 5, 10, 30, or 40 cycles, other combinations of the factors are needed to achieve the objective. If the breeding objective is to maximize rates of genetic improvement in five to 10 cycles of recurrent selection then there are two best options: 1. Genomic selection using RRBLUP estimated phenotypic values followed by an HN mating design in family island populations with FC migration policies, or 2. Genomic selection using RRBLUP estimated phenotypic values followed by a GM design in family island populations with FC migration policies (respectively designated as IM-GS-HN-FC and IM-GS-GM-FC in Table 2). If the objectives are to maximize both short-term and long-term gains then the best solution was obtained by selecting with RRBLUP estimated phenotypic values followed by an HN/CR/GM in family island populations and applying an FC migration policy (designated IM-GS-HN-FC/IM-GS-CR-FC/IM-GS-GM-FC in Table 2). Among the combinations applied on centralized populations, WGS followed by the CR mating design or RM resulted in largest long-term gains, while selection using RRBLUP estimated phenotypic values followed by an HN mating design provided the greatest short-term gains. It is important to note that the relative ranking of methods will change with the weights for short-term and long-term objectives.

**TABLE 2:** Trade-off table for strategies.

Objectives	Objectives weighted equally for gain across 40 cycles	Method				
		IM-FC-GS-HN	IM-FC-GS-CR	IM-FC-WGS-HN	IM-FC-GS-GM	IM-FC-WGS-GM
**Rs in 5 cycles (Rank)**	**0.2**	0.14 **(5)**	0.13 **(13)**	0.13 **(13)**	0.13 **(13)**	0.13 **(13)**
Rs in 10 cycles **(Rank)**	**0.2**	0.29 **(3)**	0.28 **(7)**	0.29 **(3)**	0.27 **(11)**	0.26 **(12)**
Rs in 20 cycles **(Rank)**	**0.2**	0.53 **(1)**	0.51 **(4)**	0.51 **(4)**	0.48 **(8)**	0.49 **(7)**
Rs in 30 cycles **(Rank)**	**0.2**	0.59 **(7)**	0.59 **(7)**	0.59 **(7)**	0.59 **(7)**	0.59 **(7)**
Rs in 40 cycles **(Rank)**	**0.2**	0.59 **(13)**	0.6 **(8)**	0.59 **(13)**	0.61 **(4)**	0.61 **(4)**
**Weighted rank**		**1**	**2**	**3**	**4**	**4**

**Objectives**	**Objectives weighted highly for gain in the first 20 cycles**	**Method**				
		**IM-FC-GS-HN**	**IM-FC-WGS-HN**	**IM-FC-GS-CR**	**CE-GS-CR**	**CE-GS-GM**

Rs in 5 cycles **(Rank)**	**0.5**	0.14 **(5)**	0.13 **(13)**	0.13 **(13)**	0.14 **(5)**	0.14 **(5)**
Rs in 10 cycles **(Rank)**	**0.2**	0.29 **(3)**	0.29 **(3)**	0.28 **(7)**	0.28 **(7)**	0.28 **(7)**
Rs in 20 cycles **(Rank)**	**0.1**	0.53 **(1)**	0.51 **(4)**	0.51 **(4)**	0.44 **(18)**	0.44 **(18)**
Rs in 30 cycles **(Rank)**	**0.1**	0.59 **(7)**	0.59 **(7)**	0.59 **(7)**	0.47 **(22)**	0.47 **(22)**
Rs in 40 cycles **(Rank)**	**0.1**	0.59 **(13)**	0.59 **(13)**	0.60 **(8)**	0.47 **(26)**	0.47 **(26)**
**Weighted rank**		**1**	**2**	**3**	**4**	**4**

*Trade-off table to support the decision for selecting the best strategy to achieve objectives including maximum gain in 5, 10, 20, 30, and 40 cycles of recurrent selection. The methods are ranked for each of the objectives based on standardized genetic responses. The absolute genotypic response values for each of the methods are provided along with the ranking of the method for the specific objective in bold numeric in parenthesis. Two sets of objective weights are provided to define the relative importance of the objectives: (i) the weighted rank of methods are estimated with more emphasis on the first 20 cycles (top), (ii) the weighted rank of methods are estimated with equal emphasis on the first and last 20 cycles (bottom). The best five methods among the 60 methods for each of the weighted objectives are presented. The simulations are provided for 400 simulated QTL responsible for 70% of phenotypic variability. Migration policies include “Isolated Selection,” “Best Island,” “Random Best,” and “Fully Connected.” Other migration factors are set to constant values: migration frequency - 2, migration direction - 2 (bi-directional), and migration size - 2. Selection methods include PS, Phenotypic Selection; GS, Genomic Selection; and WGS, Weighted Genomic Selection. Mating designs include HN (Hub Network), CR (Chain rule), RM, Random Mating; and GM, Genomic Mating method.*

## Discussion

### Significance

The challenge of finding optimal trade-offs among competing genetic improvement objectives has usually been approached by combining selection and crossing in a single step without consideration of population structure (Akdemir and Sánchez, 2016; De Beukelaer et al., 2017; Akdemir et al., 2019; Allier et al., 2019a, b, 2020; Ramasubramanian and Beavis, 2020). Akdemir and Sánchez (2016) combined selection and mating in their GM method. De Beukelaer et al. (2017) used weighted selection indices to maximize gain while retaining a threshold level of diversity. Among the three diversity measures they tested, indices that incorporate diversity measures to minimize loss of rare favorable alleles and minimize heterozygosity resulted in responses that were greater than WGS with truncation selection. Including diversity measures in a set offered advantage over truncation selection, as selected mate pairs retained rare favorable alleles better than WGS coupled with RM design. Allier et al., 2019a, b included the impact of within-family selection to maximize genetic gain while minimizing loss of genetic variance, but they did not consider migration among families.

Ramasubramanian and Beavis (2020) investigated GS methods for the genetic improvement of soybean, but only considered the HN mating design applied among F_5_-derived lines regardless of their family affiliation. Herein, we approached the challenge by disentangling breeding decisions into four distinct groups: (1) organization of the breeding population, (2) selection methods, (3) mating designs, and (4) migration policies. Each of these were divided into possible alternatives within each group and treated as independent factors in orthogonal treatment combinations.

As with our previous investigation, we found that the fastest rates of genetic improvement resulted when GS followed by the HN mating design is applied to the centralized populations (Ramasubramanian and Beavis, 2020). When combined, these three decisions have reinforcing effects on responses to selection. At the other extreme, when WGS is applied to populations organized as family islands followed by either CR or RM, the tendency of all three to retain genetic diversity reinforce each other resulting in the largest genotypic values, but only after many cycles of selection. Because the slopes of the curves resulting from WGS and PS at 40 cycles are still positive, it is possible that both selection methods could continue to produce greater genetic potential with more cycles of selection. In previous comparative studies, WGS produced long-term responses that are similar to methods such as Optimal Contribution Selection and Expected Maximum – Haploid Value (Daetwyler et al., 2015; Müller et al., 2018). Herein when we applied WGS to centralized lines followed by the GM design, the genotypic values at the limits to response were greater than the genotypic values obtained with PS or GS followed by GM. This combination also retained the largest values for heterozygosity and favorable alleles across more cycles. However, the differences between responses to GS and WGS followed by GM were not significant when applied to the populations organized into family islands with migration among islands.

Between the extreme response curves, it was also possible to find many response curves with intermediate trade-offs between the objectives. For example, applying WGS to lines that were not organized into islands followed by HN provided greater response rates than other combinations of factors involving WGS. Selection among lines organized into family islands resulted in responses that were larger or comparable to responses from centralized populations for only a limited number of combinations of mating design (GM) and migration policies (RB and FC). This may be due to the small numbers of related lines on each island (20 × smaller than the centralized population). With such a small number, selection can deplete all the genetic variance within the first 10–15 cycles as demonstrated in isolated selection. When there is no migration, which is the major source of new genetic variability, the populations realized only 10%–15% of maximum potential in the founder populations even while optimizing for sustainable gain using the GM method. A relaxed selection intensity, where the top 20% of the lines in each island are selected can sustain responses for longer cycles as demonstrated in centralized and island selection with migration (Supplementary Table 3).

As expected, even with small numbers of lines per island, migration had a positive impact on the outcomes. It is known that intermediate levels of migration rate result in optimal trade-offs between gain and diversity (Skolicki, 2007; Skolicki and Jong, 2007: Obolski et al., 2017). However, the range of intermediate parameter values depend on the specific context. In our study, responses in family islands were larger than selection responses in centralized populations only when migration events happened every cycle or once in two cycles. When migration happened once in three cycles of selection, the rates of responses in the early cycles were very low resulting in fewer cycles of response to selection and lower genotypic values as the limits to selection were approached. Migration size and direction did not have any significant impact on response within the small range of parameter values we tested for migration size and direction.

Also, we retained the best line, in terms of the selection metric, within island during migration events and replaced the second best line in the ranked list of selected lines with the immigrant for the BI and RB policies, whereas, for the FC policy, lines that are ranked from 2–6 are replaced. This replacement policy allows crossing between lines that are best within islands and immigrant lines from islands with the highest selection metric resulting in high rates of response within islands. We hypothesize that other policies that replace lines with low selection metric value with high selection metric values from immigrant islands will reduce genetic diversity within islands and result in different outcomes compared to the policy we have implemented.

Nonetheless, we found a very good trade-off among the competing objectives. If GS was applied to lines on FC islands and the selected lines were mated according to the pareto-optimal crosses identified using GM, then the combination preserved genetic variance for long-term gain with little penalty relative to the realized rates of improvement in early cycles by GS and the HN mating design. Yabe et al. (2016) have reported outcomes from recurrent GS in rice populations using the following three migration policies: centralized populations also referred to as bulked GS, discrete GS that corresponds to the fully isolated selection, and island GS which corresponds to the island model selection. In their study, where they used the CR for crossing, GS on centralized populations showed larger responses in the seven to eight cycles compared to isolated and island selection, whereas island GS demonstrated larger responses than the centralized and isolated GS after 12 cycles of selection. Similarly, in this study, GS on centralized populations with CR mating design resulted in larger long-term responses compared to most of the island GS except when an FC migration policy was used, where the responses were roughly similar in the first 10 cycles. Moreover, the responses were larger in the late cycles with Island GS and FC policy than in the centralized policy with CR mating design similar to the outcomes in the Yabe et al. (2016) study.

In another study, Technow et al. (2021) have investigated the impact of breeding program structure in maize hybrid development. They observed that a centralized policy provided the best responses, when the genetic architecture is completely additive. This roughly corresponds to the results we observed, where GS in centralized populations showed the largest short-term responses with the HN mating design, which is similar to the disproportional contributions in Technow et al. (2021). They also noted that, as the genetic complexity increased, the distributed and isolated policies provided larger responses. In summary, motivated by Akdemir and Sánchez (2016) and Yabe et al. (2016), we demonstrate that it is possible to design breeding strategies to produce near maximal rates of genetic improvement while retaining maximal genetic potential for long-term genetic improvement.

### Future Research

By framing breeding strategies as orthogonal combinations of population structure, selection methods, mating designs, and migration policies, we illustrated the potential of the approach for a small arbitrary soybean genetic improvement project. We did not consider the relative emphasis of objectives and constraints for any specific genetic improvement project. Consider first the structure of breeding populations. We compared a centralized structure of lines with family islands created by individual crosses among the founders and then we selected within and among islands according to the same criteria. This might make sense within a single soybean genetic improvement project for lines adapted to MGs II and III. Alternatively, individual breeding projects might be considered breeding islands.

There are six public soybean genetic improvement projects adapted to MGs II and III. There are likewise about the same number of commercial soybean genetic improvement projects in the same MGs. All of these projects began at different times and were initiated with unique, albeit overlapping, germplasm resources (Mikel et al., 2010). While all of the projects select lines with greater genotypic values for yield, the yield values are obtained from different, overlapping, environments.

From the perspective of soybean genetic improvement across regions within MGs II and III, each genetic improvement project can be represented as an island where genotypes are exchanged among project islands based on annual evaluations in uniform regional trials and according to legal licensing rules. In practice, breeding projects exchange projects only the best performing lines adapted to similar environmental conditions. Nonetheless, soybean breeders will maintain useful genetic variability by exchanging lines among island projects. An advantage is that diversity among islands increases with selection, even when within-island diversity decreases. Eventually, beyond 40 cycles of recurrent selection, genetic variability among islands will decrease as genetic variability among islands is lost to selection.

Future investigations of breeding strategies to optimize trade-offs between rates of genetic gains and retention of useful genetic variance in soybean adapted to MGs II and III should consider population structures within island projects that more accurately reflect those that currently exist. Also, future investigations should simulate genetic architectures with genotype × environment effects. It is well known that a line adapted to one environment may not perform well in other environments, and it is possible to define fitness values so that they include environmental effects. Third, future investigations should consider a broader set of migration rules and policies. The FC migration policy is considered the upper bound of island models as all islands are connected to every other island with maximum migration rates among islands. While our results indicate that this policy provided the best long-term genotypic values, it remains to be tested whether it will provide the best results for genetic architectures with genotype by environment interaction effects.

Fourth, we need to recognize islands in time because every cycle of selection discards useful genetic variability. A soybean germplasm resource project was set up (Mikel et al., 2010) to recover useful genetic variability lost during domestication of soybean (Nelson, 2011). Rather than trying to build long bridges to islands located in the distant past, our results suggest that there should be a large amount of useful genetic variability that was discarded in the first few cycles of modern soybean breeding. For that matter, until response to selection reaches the half-life for the population, large amounts of useful genetic variability can probably be recovered from islands represented by recent cycles of discarded lines. These conjectures should be preceded by simulations to determine the potential benefit and costs associated with sampling lines in recently discarded islands.

Fifth, it should be clear that a predefined mating design does not take advantage of opportunities created by each cycle of progeny to optimize outcomes according to most project objectives. Thus, there continues to be a need for algorithms that efficiently and effectively identify crosses from among genotypes produced by each cycle of selection. It is tempting to adopt and investigate all evolutionary algorithm strategies. However, only a subset is relevant to the practice of plant breeding (Hagan et al., 2012). For example, mutation and recombination rates can be controlled in computational evolutionary algorithms, whereas plant breeders cannot regulate these with current practices. Nonetheless there are many opportunities for cross-disciplinary research between evolutionary computing and plant breeding. There is a large body of literature concerning the properties of evolutionary algorithms and factors and strategies that affect convergence rates and quality of solutions (Goldberg, 1989; Goldberg and Deb, 1992; Whitley et al., 1999; Skolicki, 2007; Skolicki and Jong, 2007; Črepinšek et al., 2013; Obolski et al., 2017), and working with computational scientists should reveal novel methods to maximize the genetic potential of a breeding population in a minimum number of cycles.

Akdemir and Sánchez (2016) proposed only one of many possible GAs to identify pareto-optimal solution pairs. An approach introduced by Gaur and Deb (2016) and Mittal et al. (2020) would use statistical methods such as clustering and machine learning to unravel relationship among pareto-optimal solutions. The statistical knowledge can be used to improve the search for optimal solutions and establish several cycles of optimization. Conceptually, unveiling any relationship among pareto-optimal pairs in a genotypic space is likely to provide new knowledge regarding the characteristics of such complementary pairs. In addition, modeling responses with a first-order recurrence equation or a non-linear mixed effects model to predict the half-life and asymptotic limits of selection have potential to improve the efficiency of GAs by providing repair operators to alter the trajectory of population evolution towards the desired optimal trade-offs.

Lastly, consider the challenge of stating explicit relative emphasis on objectives and definition of constraints for any specific genetic improvement project. As noted previously, this challenge exists because it requires assigning economic and agronomic value of short-term genetic gains vs. the forecasted value of useful genetic variants that may be discarded each cycle of selection. As a thought experiment, note that the trade-off objectives can be reduced to a single “grand” objective of creating a genotype (line) with the genotypic value equal to the full genetic potential of the founders in a single cycle. For a genetic architecture consisting of two alleles at a single locus, achieving the single grand objective is trivial. Also, it is possible to imagine that the grand objective can be achieved for a complex genetic architecture with infinite resources. Clearly, given genetic architectures of complex traits and resource constraints, there are no feasible solutions to the grand objective, but it is a useful reference to serve as the goal.

In summary, we have evaluated and suggested several novel combinations of existing genomic selection methods, mating designs, and migration rules that resulted in improved responses. The study has demonstrated the potential of these new approaches, which integrate the strengths of whole-genome level information, prediction modeling, and optimization methods to contribute to the development of decision support systems for real plant breeding programs.

## Data Availability Statement

Simulated data and software codes are available as part of the R package “SoyNAMSelectionMethods” (Supplementary File 3). Documentation for downloading and using the package are available at http://gfspopgen.agron.iastate.edu/SoyNAMSelectionMethods_v2_2020.html. The SoyNAM founder genotypic and phenotypic data are available in SoyBase (Grant et al., 2010). The original contributions presented in the study are included in the article/Supplementary Material, further inquiries can be directed to the corresponding author/s.

## Author Contributions

VR and WDB contributed to the conception and design of the study. VR wrote the software code for simulations and performed statistical analysis. VR and WDB are responsible for the interpretation of the analysis. VR wrote the first draft of the manuscript. VR and WDB contributed to revisions, preparation of the final draft and approved the submitted version.

## Conflict of Interest

The authors declare that the research was conducted in the absence of any commercial or financial relationships that could be construed as a potential conflict of interest.

## Publisher’s Note

All claims expressed in this article are solely those of the authors and do not necessarily represent those of their affiliated organizations, or those of the publisher, the editors and the reviewers. Any product that may be evaluated in this article, or claim that may be made by its manufacturer, is not guaranteed or endorsed by the publisher.

## Abbreviations

SM: Selection Method
MD: Mating Design
MP: Migration policy
PS: Phenotypic selection
HN: Hub Network
BI: Best Island
GS: Genomic selection
CR: Chain Rule
RB: Random Best
WGS: Weighted Genomic Selection
RM: Random Mating
FC: Fully Connected
IM: Island Model
GM: Genomic Mating
GA: Genetic Algorithm.
