# Supplementary material for: Strategies to Assure Optimal Trade-Offs Among Competing Objectives for the Genetic Improvement of Soybean
Source: Front Genet. 2021 Sep 24;12:675500. doi: 10.3389/fgene.2021.675500 (PMC8497982; doi:10.3389/fgene.2021.675500)
Supplement: Supplementary file 1 [file Data_Sheet_1.PDF]

SUPPLEMENTARY FILE 2: Documentation for Fitting NLME Models and Analyses of Variance

Table of Contents

NLME Models Part I - Multi-level Random Intercept Model 1

NLME Models Part II - Single-level Random Intercept Model.....................14

NLME Models Part III - Correlation Structure..24

Non-linear Models

Vishnu

January 7, 2021

Non-linear response curve modelling in recurrent selection with non-linear least squares fit and non-linear mixed effects models

The purpose of this analysis is to identify factors that have significant impact on rates and limits of response. Genotypic values are modelled as a recurrence equation given below with two parameters alpha and beta. Non-linear models can be fit as fixed effects model with 'nlsList' and mixed effects model with 'nlme'. ANOVA of 'nlme' models allows us to test hypothesis regarding models. Eventhough the analysis described here doesn't provide conclusions regarding significance of specific levels of factors, it can provide evidence for hypothesis regarding factors with significant impact on dynamics of response.'nlshelper' and 'nlstools' packages provide additional support functions.

Package Installation

- Download 'SoyNAMSelectionMethods_0.1.0.tar.gz' from here (http://gfspopgen.agron.iastate.edu/SoyNAM_SelectionMethods.html) to current working directory.
- Install dependencies from CRAN:
- Install 'SoyNAMSelectionMethods' package from local repository by setting 'repos=TRUE' in 'install.packages'
- Install 'nlme', 'nlshelper', 'nlstools'

```
if( length(which(rownames(installed.packages()) %in% "nlme")) ==0){

    install.packages("nlme")
 }
if( length(which(rownames(installed.packages()) %in% "nlshelper")) ==0){

    install.packages("nlshelper")
}
if(length(which(rownames(installed.packages()) %in% "nlstools")) ==0){
    install.packages("nlstools")
}

  library(nlme)
  library(nlshelper)
  library(nlstools)
```

Data for non-linear fits and ANOVA of non-linear models

- Data - Average genotypic value of 2000 F5 RILs for 40 cycles for each unique combination of factors or condition (40 (data points/condition) with 5 replicates per condition - 200)
- 60 unique conditions 2 (PT) x 3 (Selection Method) x 4 (Mating Design) x 5 (Migration Policies)
- Total number of data points - 60*5*40 = 12,000

```
## Load simresults data
data(package="SoyNAMSelectionMethods")

dim(Data)
```

```
## [1] 12000    10
```

```
colnames(Data)
```

```
##  [1] "Population"      "SelectionMethod" "BreedDesign"     "MigPolicy"
##  [5] "nQTL"            "heritability"    "Cond"            "Rep"
##  [9] "Cycle"           "G"
```

```
########################

 dim(Table)
```

```
## [1] 60  6
```

```
dim(Data)
```

```
## [1] 12000    10
```

```
dim(Data_CondFactors)
```

```
## [1] 12000    21
```

```
#### Checks
levels(factor(Table[,1]))
```

```
## [1] "IM" "NI"
```

```
length(levels(factor(Data_CondFactors[,21])))
```

```
## [1] 60
```

Recurrence equation

$$y_c = \alpha^c\,(y_0 - y') + y'$$

$$y' = \frac{\beta}{1-\alpha}$$

$$y_c - \;\; y \;\; at \;\; cycle \;\; 'c'$$

$$y_0 \;\; - \;\; initial \;\; value \;\; of \;\; y$$

Set formula for non-linear recurrence equation in R with 'as.formula'

```
formulaExp <- as.formula(as.numeric(as.character(G)) ~ (((alpha^as.numeric(as.character(Cycl
e)))*((-beta)/(1-alpha)) + (beta)/(1-alpha))))
```

Create grouped data objects

Group data based on single or unique combinations of factors using groupedData function

```
formulaExp <- as.formula(as.numeric(as.character(G)) ~ (((alpha^as.numeric(as.character
(Cycle)))*((-beta)/(1-alpha)) + (beta)/(1-alpha))))


grouped_data <- groupedData(as.numeric(as.character(G)) ~ as.numeric(as.character(Cycl
e)) |as.factor(as.character(Cond)), data = Data)



grouped_data_PT <- groupedData(as.numeric(as.character(G)) ~ as.numeric(as.character(Cy
cle)) | as.factor(Population),data = Data)

grouped_data_BD <- groupedData(as.numeric(as.character(G)) ~ as.numeric(as.character(Cy
cle)) | as.factor(as.character(BreedDesign)),data = Data)

grouped_data_SM <- groupedData(as.numeric(as.character(G)) ~ as.numeric(as.character(Cycl
e)) | as.factor(SelectionMethod),data = Data)


grouped_data_MP <- groupedData(as.numeric(as.character(G)) ~ as.numeric(as.character(Cycl
e)) | as.factor(as.character(MigPolicy)),data = Data)




grouped_data_PT_BD <- groupedData(as.numeric(as.character(G)) ~ as.numeric(as.character
(Cycle)) | as.factor(Population)/as.factor(as.character(BreedDesign)),data = Data)

grouped_data_PT_SM <- groupedData(as.numeric(as.character(G)) ~ as.numeric(as.character
(Cycle)) | as.factor(Population)/as.factor(as.character(SelectionMethod)),data = Data)

grouped_data_PT_MP <- groupedData(as.numeric(as.character(G)) ~ as.numeric(as.character(Cy
cle)) | as.factor(Population)/as.factor(as.character(MigPolicy)),data = Data)


grouped_data_BD_SM <- groupedData(as.numeric(as.character(G)) ~ as.numeric(as.character(C
ycle)) | as.factor(as.character(BreedDesign))/as.factor(as.character(SelectionMethod)),data = D
ata)

grouped_data_BD_MP <- groupedData(as.numeric(as.character(G)) ~ as.numeric(as.character(Cyc
le)) | as.factor(as.character(BreedDesign))/as.factor(as.character(MigPolicy)),data = Data)

grouped_data_SM_MP <- groupedData(as.numeric(as.character(G)) ~ as.numeric(as.character
(Cycle)) | as.factor(as.character(SelectionMethod))/as.factor(as.character(MigPolicy)),data = D
ata)




##

grouped_data_PT_BD_SM <- groupedData(as.numeric(as.character(G)) ~ as.numeric(as.character(C
```

```
ycle)) | as.factor(Population)/as.factor(as.character(BreedDesign))/as.factor(as.character(SelectionMethod)),data = Data)

grouped_data_PT_BD_MP <- groupedData(as.numeric(as.character(G)) ~ as.numeric(as.character(Cycle)) | as.factor(Population)/as.factor(as.character(BreedDesign))/as.factor(as.character(MigPolicy)),data = Data)

grouped_data_PT_SM_MP <- groupedData(as.numeric(as.character(G)) ~ as.numeric(as.character(Cycle)) | as.factor(Population)/as.factor(as.character(SelectionMethod))/as.factor(as.character(MigPolicy)),data = Data)

grouped_data_BD_SM_MP <- groupedData(as.numeric(as.character(G)) ~ as.numeric(as.character(Cycle)) | as.factor(as.character(BreedDesign))/as.factor(as.character(SelectionMethod))/as.factor(as.character(MigPolicy)),data = Data)

### 1 quartet

grouped_data_PT_BD_SM_MP <- groupedData(as.numeric(as.character(G)) ~ as.numeric(as.character(Cycle)) | as.factor(as.character(Population))/as.factor(as.character(BreedDesign))/as.factor(as.character(SelectionMethod))/as.factor(as.character(MigPolicy)),data = Data)

############
```

Plot grouped data

Grouped data plots allow us to visualize the dynamics of genotypic values that are grouped in to levels of factors or levels of nested combination of factors. In the plots below, observe any difference in dynamics of average genotypic values of a population in 40 cycles of recurrent genomic and phenotypic selection among levels of a factor in data.

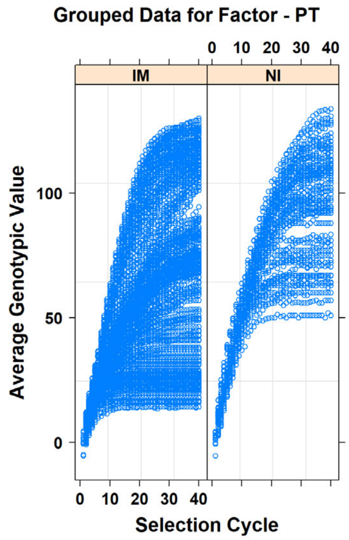
Grouped Data for Factor - PT
IM
NI
Average Genotypic Value
Selection Cycle
0
50
100
0 10 20 30 40

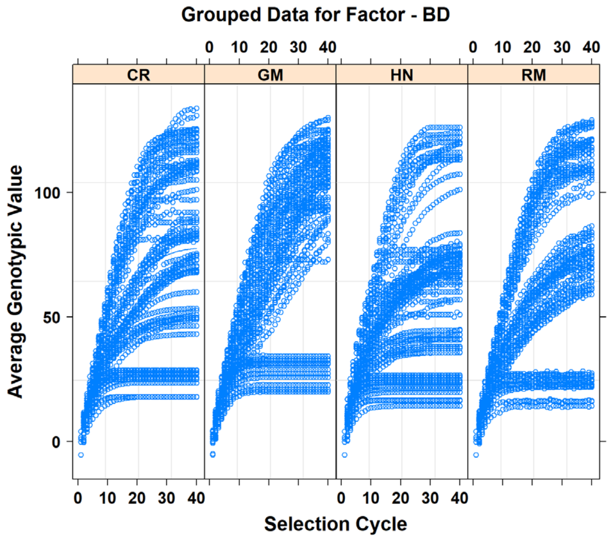
Grouped Data for Factor - BD
CR
GM
HN
RM
Average Genotypic Value
Selection Cycle
0
50
100
0 10 20 30 40

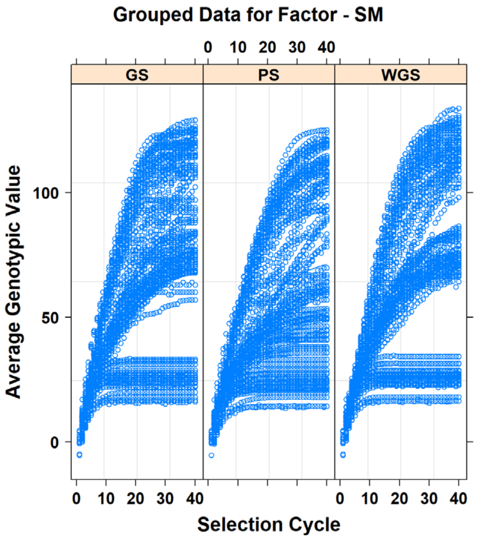
Grouped Data for Factor - SM
GS
PS
WGS
Average Genotypic Value
Selection Cycle
0
50
100
0 10 20 30 40

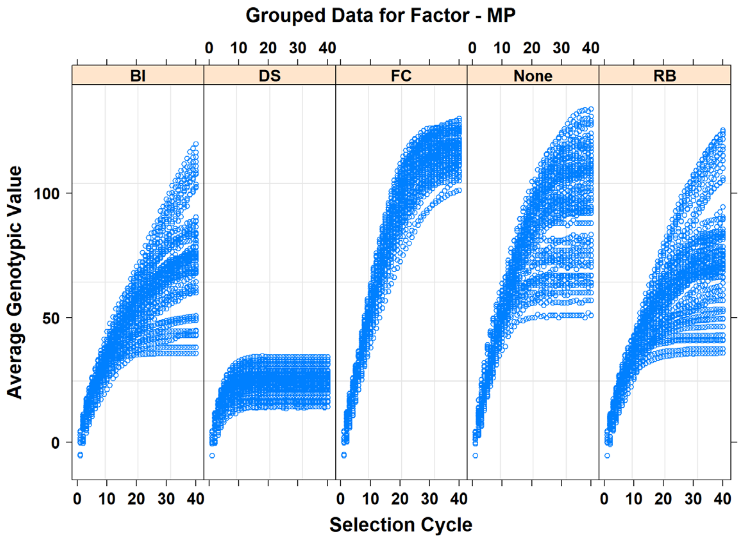

Non-linear least squares fit (nlsList) for non-linear response curve in recurrent selection

nlslist model fit with grouped data objects

nlsList fits fixed effects only nls model with grouped data for each of the levels in the group. Estimates of alpha and beta for each level in single or nested groups can be retrieved and plotted for analysis.

Plot residuals of estimates in nlsList object

Standardized residuals vs fitted values plots for five factors and combinations of all 360 factors. Look for equally spaced spread across fitted values for models that better account for errors.

```
  par(font=2,cex=1.5)
  b <- plot(fit_Data_List_all,cex=1.0,main="Residual_Plot_nlsList_All_Conditions")
  print(b);
  flush.console()
Sys.sleep(5)
```

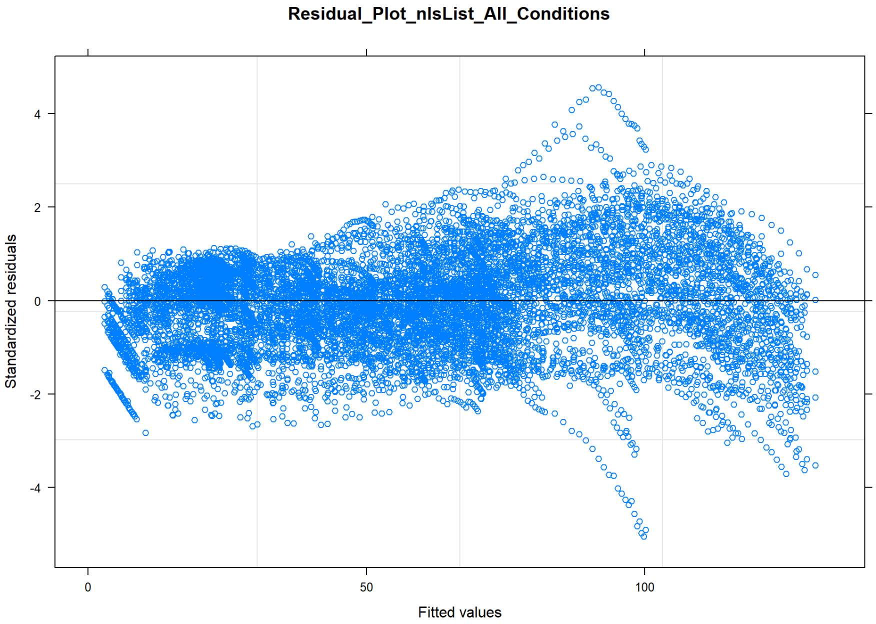

Plot interval plots of estimates in nlsList objects

Interval plots for estimates of alpha and beta for levels of single factors and levels of nested factors. Look for non-overlapping intervals of estimates among groups in these plots. When non-overlapping intervals are present among levels for any parameter, deviations of those parameters can be modelled as random effects in mixed effects model with nlme. nlsList function needs start values of parameters for models. In some cases, control parameters defining max number of iterations need to be increased. Check nlsList function documentation for more info.

```
            par(font=2,cex=1.5)
            b <- plot(intervals(fit_Data_List_all),cex=1.0,ylab="",main="Interval_Plot_nlsList_
All_Conditions")
            print(b);
            flush.console()
          Sys.sleep(5)
```

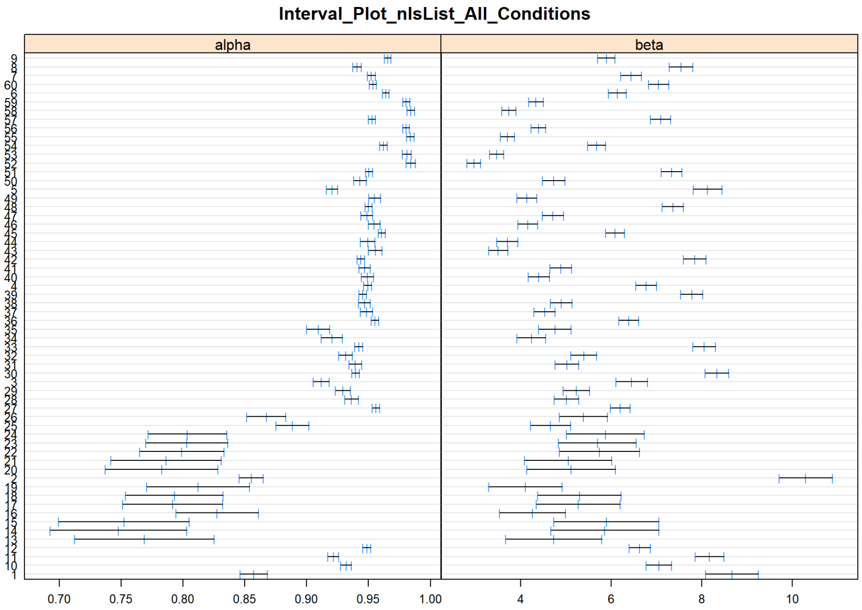

Anova for nlsList

anova_nlslist works only on one full nlslist and a reduced nls model grouped on a variable. So only pairwise comparisons are possible. Summary of anova_nlsList for pairwise comparisons of i) nlsList fit with two groups based on heritability and nls model 'fit_Data_noCond' fit on complete data with no grouping factor . ii) nlsList fit with all 60 unique combinations of factors and nls model 'fit_Data_noCond' fit on complete data with no grouping factor. For pairwise comparison of other nlsList models, use the same syntax for anova_nlsList with nlsList models from previous section.

```
## 60 curves vs 1 curve

anova_nlslist(fit_Data_List_all,fit_Data_noCond)
```

```
## Analysis of Variance Table
##
## Model 1: as.numeric(as.character(G)) ~ (((alpha^as.numeric(as.character(Cycle))) * ((-beta)/(1 - alpha)) + (beta)/(1 - al
pha)))
## Model 2: as.numeric(as.character(G)) ~ (((alpha^as.numeric(as.character(Cycle))) * ((-beta)/(1 - alpha)) + (beta)/(1 - al
pha)))
##   Res.Df Res.Sum Sq  Df  Sum Sq F value    Pr(>F)
## 1  11998    7938406
## 2  11880     361080 118 7577326  2112.7 < 2.2e-16 ***
## ---
## Signif. codes:  0 '***' 0.001 '**' 0.01 '*' 0.05 '.' 0.1 ' ' 1
```

ANOVA nls_List Output has 6 columns Anova_nlsList in 'nlshelper' R pkg (https://rdrr.io/cran/nlshelper/man/anova_nlslist.html)

i. Residual Degrees of Freedom (Df): total number of observations - number of parameters (in this case, 14400-2 = 143998 for Model 1, 14400-4=143996for Model 2), etc:

ii. Residual Sums of Squares iii)Df - change in the residual Df from model1 to model2
iii. Sums of Squares (Change in the Residual Sums of Squares from model1 to model2)
iv. F-Statistic
v. P-value

Non-linear mixed Effects Models (nlme) for non-linear response curve in recurrent selection

nlme fits mixed models for non-linear functions. Population means of parameters are treated as fixed effects. Deviations from mean within groups are treated as random effects in a random intercept model. For nlme, any grouping factor is defined in the random part of the model and complete data is used instead of grouped data. It is better to fit nlme with fixed effects estimated from nlsList objects as starting values. 'nlme' fit uses the same formula expression as nlsList. If there are errors or when convergence fails, try changing control parameters or set opt="nlm"

Fit mixed-effects non-linear models with 'nlme' function on data

Optimal random effects structure in random intercept models for 'nlme' (non-linear models) with 'nlme' function on data

ANOVA for nlme. Multi-level anova can be performed with nlme fit objects

Anova for nlme output has 8 columns : (José C. Pinheiro and Douglas M. Bates.,2000: Pg-83)

i) Model id ii) df - degrees of freedom - number of parameters in the model iii) AIC - -2(loglikelihood) + 2*number of paramemeters iv) BIC - -2(loglikelihood) + log(N)* number of paramemeters v) logLikelihood vi) Test - vii) L.Ratio viii) p-value

NLME ANOVA Table

	df	AIC	BIC	logLik	Test	L.Ratio	p-value
fit_Data_nlme_Cond	6	75831.43	75875.79	-37909.72		NA	NA
fit_Data_nlme_PT	6	110715.11	110759.47	-55351.56		NA	NA
fit_Data_nlme_BD	6	111546.89	111591.25	-55767.45		NA	NA
fit_Data_nlme_SM	6	111671.50	111715.86	-55829.75		NA	NA
fit_Data_nlme_MP	6	92144.61	92188.97	-46066.30		NA	NA

	df	AIC	BIC	logLik	Test	L.Ratio	p-value
fit_Data_nlme_PT_BD	9	109770.38	109836.92	-54876.19	5 vs 6	17619.77358	0.0000000
fit_Data_nlme_PT_SM	9	110303.76	110370.30	-55142.88		NA	NA
fit_Data_nlme_PT_MP	9	92150.61	92217.14	-46066.30		NA	NA
fit_Data_nlme_BD_SM	9	111214.34	111280.88	-55598.17		NA	NA
fit_Data_nlme_BD_MP	9	84098.22	84164.75	-42040.11		NA	NA
fit_Data_nlme_PT_BD_SM_MP	15	75834.13	75945.02	-37902.06	15 vs 16	13.99843	0.0029073

ANOVA-nlme Interpretation

'fit_Data_nlme_Cond' (the last row in anova table) has the least AIC and BIC scores. So the model that fits separate curves for each of 60 combinations of treatment factors is the best fit. Significance tests also show that models fit with single factors are significantly different from models fit with two nested group levels one for each factor. Models fit with two nested group levels are significantly different from models fit with three nested groups and the models with three nested group levels are significantly different from four level models. All the single and multi-level models are significantly different from models fit on all group factors. From this we conclude that all factors have significant effect on the responses.

NLME parameter estimates (alpha and beta) for 60 combinations of treatment factors. We can plot predicted genotypic values with these parameter estimates using the recurrence equation and compare it with observed values.

```
#
## Co-efficients alpha and beta for all 60 combinations of factors


coef_NLME_All<- coef(fit_Data_nlme_Cond)

Treatment_Group <- c(1:60)
Coef_NLME_Indices <- as.numeric(row.names(coef_NLME_All))
Coef_NLME_Id <- as.numeric(row.names(coef_NLME_All))
Coef_NLME_All_Table <- cbind(coef_NLME_All,Coef_NLME_Id)

Treatment_Group_Table <- cbind(Table,Treatment_Group)
dim(Coef_NLME_All_Table)
```

```
## [1] 60  3
```

```r
Coef_NLME_All_Table[1:5,]
```

```
##        alpha     beta Coef_NLME_Id
## 1  0.8593718 8.550496            1
## 10 0.9319007 7.039723           10
## 11 0.9219063 8.142463           11
## 12 0.9485629 6.620362           12
## 13 0.7827423 4.477185           13
```

```r
dim(Treatment_Group_Table)
```

```
## [1] 60  7
```

```r
Treatment_Group_Table[1:5,]
```

```
##           Population SelectionMethod BreedDesign MigPolicy nQTL  heritability
## Table_Row "NI"       "PS"            "HN"        "None"    "400" "0.7"
## Table_Row "NI"       "GS"            "HN"        "None"    "400" "0.7"
## Table_Row "NI"       "WGS"           "HN"        "None"    "400" "0.7"
## Table_Row "NI"       "PS"            "CR"        "None"    "400" "0.7"
## Table_Row "NI"       "GS"            "CR"        "None"    "400" "0.7"
##           Treatment_Group
## Table_Row "1"
## Table_Row "2"
## Table_Row "3"
## Table_Row "4"
## Table_Row "5"
```

```r
colnames(Treatment_Group_Table)[7] <- "Id"
colnames(Coef_NLME_All_Table)[3] <- "Id"

Coef_NLME_Output2 <- merge(Coef_NLME_All_Table,Treatment_Group_Table,by="Id")


knitr::kable(
  Coef_NLME_Output2[1:10,],booktabs=TRUE,
  caption= 'Parameter Estimates for  Conditions'
)
```

Parameter Estimates for Conditions

Id	alpha	beta	Population	SelectionMethod	BreedDesign	MigPolicy	nQTL	heritability
1	0.8593718	8.550496	NI	PS	HN	None	400	0.7
2	0.8578209	10.132547	NI	GS	HN	None	400	0.7
3	0.9118787	6.439054	NI	WGS	HN	None	400	0.7
4	0.9493282	6.762210	NI	PS	CR	None	400	0.7
5	0.9208445	8.102402	NI	GS	CR	None	400	0.7
6	0.9637965	6.133748	NI	WGS	CR	None	400	0.7
7	0.9523430	6.431542	NI	PS	RM	None	400	0.7
8	0.9409441	7.527410	NI	GS	RM	None	400	0.7
9	0.9654202	5.892474	NI	WGS	RM	None	400	0.7

Id	alpha	beta	Population	SelectionMethod	BreedDesign	MigPolicy	nQTL	heritability
10	0.9319007	7.039723	NI	PS	GM	None	400	0.7

In part 2 of NLME modeling, random intercept models are fit with single level grouping based on unique combinations of factors.

NLME_Part_II (NLME_Models_Part_II.html)

In part 3 of NLME modeling, the model with the best random intercept structure is assessed. NLME models are then fit with variance and correlation structure. These models are compared using AIC and aNOVA.

NLME_Part_III (NLME_Models_Part_III.html)

Other Information

Navigate to Evaluation Metrics Page (Evaluation_Metrics_SelectionMethods_V2.html)

For questions and concerns, please email ivanvishnu@gmail.com (mailto:ivanvishnu@gmail.com)

NLME Models Part - II

Vishnu

January 7, 2021

```
library(nlme)
library(nlshelper)
library(nlstools)
```

Data for non-linear fits and Analyses of Variance (ANOVA) of non-linear models

- Data - Average genotypic value of 2000 F5 RILs for 40 cycles for each of the unique combination of factors or conditions (40 (data points/condition) with 5 replicates per condition - 200)
- 60 unique conditions 2 (PT) x 3 (Selection Method) x 4 (Mating Design) x 5 (Migration Policies)
- Total number of data points - 60*5*40 = 12,000

```
## Load simresults data
data(package="SoyNAMSelectionMethods")
dim(Data)
```

```
## [1] 12000    10
```

```
colnames(Data)
```

```
##  [1] "Population"      "SelectionMethod" "BreedDesign"     "MigPolicy"
##  [5] "nQTL"            "heritability"    "Cond"            "Rep"
##  [9] "Cycle"           "G"
```

```
########################
dim(Table)
```

```
## [1] 60  6
```

Recurrence equation

$$y_c = \alpha^c (y_0 - y') + y'$$

$$y' = \frac{\beta}{1-\alpha}$$

$$y_c - \; y \; at \; cycle \; 'c'$$

$$y_0 \; - \; initial \; value \; of \; y$$

Set formula for non-linear recurrence equation in R with 'as.formula'

```
formulaExp <- as.formula(as.numeric(as.character(G)) ~ (((alpha^as.numeric(a
s.character(Cycle)))*((-beta)/(1-alpha)) + (beta)/(1-alpha))))
```

Create grouped data objects

Group data based on single or unique combinations of factors using groupedData function

```
formulaExp <- as.formula(as.numeric(as.character(G)) ~ (((alpha^as.numeric(as.character(Cycle)))*((-beta)/(1-alpha)) + (beta)/(1-alpha))))

grouped_data <- groupedData(as.numeric(as.character(G)) ~ as.numeric(as.character(Cycle)) |as.factor(as.character(Cond)), data = Data)

grouped_data_PT <- groupedData(as.numeric(as.character(G)) ~ as.numeric(as.character(Cycle)) | as.factor(Population),data = Data)

grouped_data_BD <- groupedData(as.numeric(as.character(G)) ~ as.numeric(as.character(Cycle)) | as.factor(as.character(BreedDesign)),data = Data)

grouped_data_SM <- groupedData(as.numeric(as.character(G)) ~ as.numeric(as.character(Cycle)) | as.factor(SelectionMethod),data = Data)

grouped_data_MP <- groupedData(as.numeric(as.character(G)) ~ as.numeric(as.character(Cycle)) | as.factor(as.character(MigPolicy)),data = Data)

grouped_data_PT_BD_Cond <- groupedData(as.numeric(as.character(G)) ~ as.numeric(as.character(Cycle)) | as.factor(as.character(Cond_PT_BD)),data = Data_CondFactors)

grouped_data_PT_SM_Cond <- groupedData(as.numeric(as.character(G)) ~ as.numeric(as.character(Cycle)) | as.factor(as.character(Cond_PT_SM)),data= Data_CondFactors)

grouped_data_PT_MP_Cond <- groupedData(as.numeric(as.character(G)) ~ as.numeric(as.character(Cycle)) | as.factor(as.character(Cond_PT_MP)),data = Data_CondFactors)

grouped_data_BD_SM_Cond <- groupedData(as.numeric(as.character(G)) ~ as.numeric(as.character(Cycle)) | as.factor(as.character(Cond_BD_SM)),data = Data_CondFactors)

grouped_data_BD_MP_Cond <- groupedData(as.numeric(as.character(G)) ~ as.numeric(as.character(Cycle)) | as.factor(as.character(Cond_BD_MP)),data = Data_CondFactors)

grouped_data_SM_MP_Cond <- groupedData(as.numeric(as.character(G)) ~ as.numeric(as.character(Cycle)) | as.factor(as.character(Cond_SM_MP)),data = Data_CondFactors)
```

```
###

#c("PT-BD-SM","PT-BD-MP","PT-SM-MP","BD-SM-MP")

grouped_data_PT_BD_SM_Cond <- groupedData(as.numeric(as.character(G)) ~ as.numeric(as.character(Cycle)) | as.factor(as.character(Cond_PT_BD_SM)) ,data = Data_CondFactors)

grouped_data_PT_BD_MP_Cond <- groupedData(as.numeric(as.character(G)) ~ as.numeric(as.character(Cycle)) | as.factor(as.character(Cond_PT_BD_MP)),data = Data_CondFactors)

grouped_data_PT_SM_MP_Cond <- groupedData(as.numeric(as.character(G)) ~ as.numeric(as.character(Cycle)) | as.factor(as.character(Cond_PT_SM_MP)),data = Data_CondFactors)

grouped_data_BD_SM_MP_Cond <- groupedData(as.numeric(as.character(G)) ~ as.numeric(as.character(Cycle)) | as.factor(as.character(Cond_BD_SM_MP)),data = Data_CondFactors)

grouped_data_PT_BD_SM_MP_Cond <- groupedData(as.numeric(as.character(G)) ~ as.numeric(as.character(Cycle)) | as.factor(as.character(Cond_PT_BD_SM_MP)),data=Data_CondFactors)

####################
```

Non-linear least squares fit (nlsList) for non-linear response curve in recurrent selection

nlslist model fit with grouped data objects

nlsList fits fixed effects only nls model with grouped data for each of the levels in the group. Estimates of alpha and beta for each level in single or nested groups can be retrieved and plotted for analysis.

```
        fit_Data_List_Cond_all <- nlsList(formulaExp,data=grouped_data,start=
list(alpha=0.1,beta=30),control=list(maxiter=200,msMaxIter=300))


###
          fit_Data_PT_List_Cond <- nlsList(formulaExp,data=grouped_data_PT,st
art=list(alpha=0.1,beta=30),control=list(maxiter=200,msMaxIter=300))
        fit_Data_BD_List_Cond <- nlsList(formulaExp,data=grouped_data_BD,star
t=list(alpha=0.1,beta=30),control=list(maxiter=200,msMaxIter=300))
        fit_Data_SM_List_Cond <- nlsList(formulaExp,data=grouped_data_SM,star
t=list(alpha=0.1,beta=30),control=list(maxiter=200,msMaxIter=300))
        fit_Data_MP_List_Cond <- nlsList(formulaExp,data=grouped_data_MP,star
t=list(alpha=0.1,beta=30),control=list(maxiter=200,msMaxIter=300))

        fit_Data_PT_BD_List_Cond <- nlsList(formulaExp,data=grouped_data_PT_B
D_Cond,start=list(alpha=0.1,beta=30),control=list(maxiter=200,msMaxIter=300))
        fit_Data_PT_SM_List_Cond <- nlsList(formulaExp,data=grouped_data_PT_S
M_Cond,start=list(alpha=0.1,beta=30),control=list(maxiter=200,msMaxIter=300))
        fit_Data_PT_MP_List_Cond <- nlsList(formulaExp,data=grouped_data_PT_M
P_Cond,start=list(alpha=0.1,beta=30),control=list(maxiter=200,msMaxIter=300))

        fit_Data_BD_SM_List_Cond <- nlsList(formulaExp,data=grouped_data_BD_S
M_Cond,start=list(alpha=0.1,beta=30),control=list(maxiter=200,msMaxIter=300))
        fit_Data_BD_MP_List_Cond <- nlsList(formulaExp,data=grouped_data_BD_M
P_Cond,start=list(alpha=0.1,beta=30),control=list(maxiter=200,msMaxIter=300))
        fit_Data_SM_MP_List_Cond <- nlsList(formulaExp,data=grouped_data_SM_M
P_Cond,start=list(alpha=0.1,beta=30),control=list(maxiter=200,msMaxIter=300))



        fit_Data_noCond <- nls(formulaExp,data=Data_CondFactors,start=list(al
pha=0.1,beta=30))


        fit_Data_PT_BD_SM_List_Cond <- nlsList(formulaExp,data=grouped_data_P
T_BD_SM_Cond,start=c(alpha=0.1,beta=30),control=list(maxiter=200,msMaxIter=30
0))

          fit_Data_PT_BD_MP_List_Cond <- nlsList(formulaExp,data=grouped_data
_PT_BD_MP_Cond,start=c(alpha=0.1,beta=30),control=list(maxiter=200,msMaxIter=
300))
```

```
fit_Data_PT_SM_MP_List_Cond <- nlsList(formulaExp,data=grouped_data_PT_SM_MP_Cond,start=c(alpha=0.1,beta=30),control=list(maxiter=200,msMaxIter=300))

fit_Data_BD_SM_MP_List_Cond <- nlsList(formulaExp,data=grouped_data_BD_SM_MP_Cond,start=c(alpha=0.1,beta=30),control=list(maxiter=200,msMaxIter=300))

fit_Data_PT_BD_SM_MP_List_Cond <- nlsList(formulaExp,data=grouped_data_PT_BD_SM_MP_Cond,start=c(alpha=0.1,beta=30),control=list(maxiter=200,msMaxIter=300))
```

Fit mixed-effects non-linear models with 'nlme' function on data

Optimal random effects structure in random intercept models for 'nlme' (non-linear models) with 'nlme' function on data

```
######

formulaExp <- as.formula(as.numeric(as.character(G)) ~ (((alpha^as.numeric(a
s.character(Cycle)))*((-beta)/(1-alpha)) + (beta)/(1-alpha))))

fit_Data_nlme_Cond <- nlme(formulaExp,data=Data_CondFactors,fixed= alpha+beta
~1,random= alpha+beta ~ 1|Cond,start= fixef(fit_Data_List_Cond_all),control=l
ist(maxiter=200,msMaxIter=300,opt="nlminb"))



fit_Data_nlme_Cond_PT <- nlme(formulaExp,data=Data_CondFactors,fixed= alpha+b
eta ~1,random= alpha+beta ~ 1|Population,start=fixef(fit_Data_PT_List_Cond),c
ontrol=list(maxiter=200,msMaxIter=300,opt="nlminb"))

fit_Data_nlme_Cond_BD <- nlme(formulaExp,data=Data_CondFactors,fixed= alpha+b
eta ~1,random= alpha+beta ~ 1|BreedDesign,start=fixef(fit_Data_BD_List_Cond),
control=list(maxiter=200,msMaxIter=300,opt="nlminb"))

fit_Data_nlme_Cond_SM <- nlme(formulaExp,data=Data_CondFactors,fixed= alpha+b
eta ~ 1,random= alpha+beta ~ 1|SelectionMethod,start=fixef(fit_Data_SM_List_C
ond),control=list(maxiter=200,msMaxIter=300,opt="nlminb"))

fit_Data_nlme_Cond_MP <- nlme(formulaExp,data=Data_CondFactors,fixed= alpha+b
eta ~ 1,random= alpha+beta ~ 1|MigPolicy,start= fixef(fit_Data_MP_List_Cond),
control=list(maxiter=200,msMaxIter=300,opt="nlminb"))



####

fit_Data_nlme_Cond_PT_BD <- nlme(formulaExp,data=Data_CondFactors,fixed= alph
a+beta ~1,random= alpha+beta ~ 1| Cond_PT_BD,start= fixef(fit_Data_PT_BD_List
_Cond),control=list(maxiter=200,msMaxIter=400,opt="nlminb"))

fit_Data_nlme_Cond_PT_SM <- nlme(formulaExp,data=Data_CondFactors,fixed= alph
a+beta ~ 1,random= alpha+beta ~ 1|Cond_PT_SM,start= fixef(fit_Data_PT_SM_List
_Cond),control=list(maxiter=200,msMaxIter=400,opt="nlminb"))

fit_Data_nlme_Cond_PT_MP <- nlme(formulaExp,data=Data_CondFactors,fixed= alph
a+beta ~ 1,random= alpha+beta ~ 1|Cond_PT_MP,start= fixef(fit_Data_PT_MP_List
```

```
_Cond),control=list(maxiter=200,msMaxIter=400,opt="nlminb"))


fit_Data_nlme_Cond_BD_SM <- nlme(formulaExp,data=Data_CondFactors,fixed= alph
a+beta ~1,random= alpha+beta ~ 1| Cond_BD_SM,start= fixef(fit_Data_BD_SM_List
_Cond),control=list(maxiter=200,msMaxIter=300,opt="nlminb"))

fit_Data_nlme_Cond_BD_MP <- nlme(formulaExp,data=Data_CondFactors,fixed= alph
a+beta ~1,random= alpha+beta ~ 1| Cond_BD_MP,start= fixef(fit_Data_BD_MP_List
_Cond),control=list(maxiter=200,msMaxIter=300,opt="nlminb"))


fit_Data_nlme_Cond_SM_MP <- nlme(formulaExp,data=Data_CondFactors,fixed= alph
a+beta ~1,random= alpha+beta ~ 1| Cond_SM_MP,start= fixef(fit_Data_SM_MP_List
_Cond),control=list(maxiter=200,msMaxIter=300,opt="nlminb"))


###

fit_Data_nlme_Cond_PT_BD_SM <- nlme(formulaExp,data=Data_CondFactors,fixed= a
lpha+beta ~ 1,random= alpha+beta ~ 1|Cond_PT_BD_SM,start=fixef(fit_Data_PT_BD
_SM_List_Cond),control=list(maxiter=200,msMaxIter=300,opt="nlminb"))

fit_Data_nlme_Cond_PT_BD_MP <- nlme(formulaExp,data=Data_CondFactors,fixed= a
lpha+beta ~ 1,random= alpha+beta ~ 1|Cond_PT_BD_MP,start=fixef(fit_Data_PT_BD
_MP_List_Cond),control=list(maxiter=200,msMaxIter=300,opt="nlminb"))

fit_Data_nlme_Cond_PT_SM_MP <- nlme(formulaExp,data=Data_CondFactors,fixed= a
lpha+beta ~ 1,random= alpha+beta ~ 1|Cond_PT_SM_MP,start=fixef(fit_Data_PT_SM
_MP_List_Cond),control=list(maxiter=200,msMaxIter=300,opt="nlminb"))

fit_Data_nlme_Cond_BD_SM_MP <- nlme(formulaExp,data=Data_CondFactors,fixed= a
lpha+beta ~ 1,random= alpha+beta ~ 1|Cond_BD_SM_MP,start=fixef(fit_Data_BD_SM
_MP_List_Cond),control=list(maxiter=200,msMaxIter=300,opt="nlminb"))

###


fit_Data_nlme_Cond_PT_BD_SM_MP <- nlme(formulaExp,data=Data_CondFactors,fixed
= alpha+beta ~ 1,random= alpha+beta ~ 1|Cond_PT_BD_SM_MP,start=fixef(fit_Data
_PT_BD_SM_MP_List_Cond),control=list(maxiter=200,msMaxIter=300,opt="nlminb"))
```

```
fit_Data_nlme_Cond_PT_BD_SM_Corr <-update(fit_Data_nlme_Cond_PT_BD_SM,correla
tion=corAR1(form= ~1|Cond_PT_BD_SM),control=list(maxiter=200,msMaxIter=300,op
t="nlminb"))


fit_Data_nlme_Cond_PT_BD_MP_Corr <-update(fit_Data_nlme_Cond_PT_BD_MP,correla
tion=corAR1(form= ~1|Cond_PT_BD_MP),control=list(maxiter=200,msMaxIter=300,op
t="nlminb"))


fit_Data_nlme_Cond_PT_SM_MP_Corr <-update(fit_Data_nlme_Cond_PT_SM_MP,correla
tion=corAR1(form= ~1|Cond_PT_SM_MP),control=list(maxiter=200,msMaxIter=300,op
t="nlminb"))


fit_Data_nlme_Cond_BD_SM_MP_Corr <-update(fit_Data_nlme_Cond_BD_SM_MP,correla
tion=corAR1(form= ~1|Cond_BD_SM_MP),control=list(maxiter=200,msMaxIter=300,op
t="nlminb"))


fit_Data_nlme_Cond_PT_BD_SM_MP_Corr <-update(fit_Data_nlme_Cond_PT_BD_SM_MP,c
orrelation=corAR1(form= ~1|Cond_PT_BD_SM_MP),control=list(maxiter=200,msMaxIt
er=300,opt="nlminb"))
```

ANOVA for NLME fits for single level unique combinations of factors using data

```
ANOVA_PM_IM_SingleLevel <-
anova(fit_Data_nlme_Cond_PT,fit_Data_nlme_Cond_BD,fit_Data_nlme_Cond_SM,fit_D
ata_nlme_Cond_MP,fit_Data_nlme_Cond_PT_BD,fit_Data_nlme_Cond_PT_SM,fit_Data_n
lme_Cond_PT_MP,fit_Data_nlme_Cond_BD_SM,fit_Data_nlme_Cond_BD_MP,fit_Data_nlm
e_Cond_SM_MP,fit_Data_nlme_Cond_PT_BD_SM,fit_Data_nlme_Cond_PT_BD_MP,
fit_Data_nlme_Cond_PT_SM_MP,fit_Data_nlme_Cond_BD_SM_MP,fit_Data_nlme_Cond_PT
_BD_SM_MP,fit_Data_nlme_Cond,fit_Data_nlme_Cond_PT_BD_SM_Corr,fit_Data_nlme_C
ond_PT_BD_MP_Corr,fit_Data_nlme_Cond_PT_SM_MP_Corr,fit_Data_nlme_Cond_BD_SM_M
P_Corr,fit_Data_nlme_Cond_PT_BD_SM_MP_Corr,fit_Data_nlme_Cond_Corr,fit_Data_n
lme_Cond_Corr_VarIdent)

####
```

ANOVA Table

```
knitr::kable(
   ANOVA_PM_IM_SingleLevel[1:15,c(-1,-2)],booktabs=TRUE,
   caption= 'NLME ANOVA Table'

)
```

NLME ANOVA Table

	df	AIC	BIC	logLik	Test	L.Ratio	p-value
fit_Data_nlme_Cond_PT	6	110715.11	110759.46	-55351.55		NA	NA
fit_Data_nlme_Cond_BD	6	111546.89	111591.25	-55767.45		NA	NA
fit_Data_nlme_Cond_SM	6	111671.50	111715.86	-55829.75		NA	NA
fit_Data_nlme_Cond_MP	6	92144.61	92188.97	-46066.30		NA	NA
fit_Data_nlme_Cond_PT_BD	6	109785.19	109829.55	-54886.60		NA	NA
fit_Data_nlme_Cond_PT_SM	6	110302.00	110346.36	-55145.00		NA	NA
fit_Data_nlme_Cond_PT_MP	6	92144.61	92188.97	-46066.30		NA	NA
fit_Data_nlme_Cond_BD_SM	6	111219.15	111263.51	-55603.57		NA	NA
fit_Data_nlme_Cond_BD_MP	6	84092.22	84136.58	-42040.11		NA	NA
fit_Data_nlme_Cond_SM_MP	6	89151.52	89195.88	-44569.76		NA	NA
fit_Data_nlme_Cond_PT_BD_SM	6	109353.62	109397.97	-54670.81		NA	NA
fit_Data_nlme_Cond_PT_BD_MP	6	84092.22	84136.58	-42040.11		NA	NA
fit_Data_nlme_Cond_PT_SM_MP	6	89151.52	89195.88	-44569.76		NA	NA
fit_Data_nlme_Cond_BD_SM_MP	6	75831.43	75875.79	-37909.72		NA	NA
fit_Data_nlme_Cond_PT_BD_SM_MP	6	75831.43	75875.79	-37909.72		NA	NA

EOF

NLME Models Part - III

Vishnu

January 7, 2021

Load libraries

```
library(nlme)
library(nlshelper)
library(nlstools)
```

Recurrence equation

$$y_c = \alpha^c \left(y_0 - y'\right) + y'$$

$$y' = \frac{\beta}{1-\alpha}$$

$$y_c - \ \ y \ \ at \ \ cycle \ \ 'c'$$

$$y_0 \ \ - \ \ initial \ \ value \ \ of \ \ y$$

Set formula for non-linear recurrence equation in R with 'as.formula'

```
formulaExp <- as.formula(as.numeric(as.character(G)) ~ (((alpha^a
s.numeric(as.character(Cycle)))*((-beta)/(1-alpha)) + (beta)/(1-al
pha))))
```

Data for non-linear fits and ANOVA of non-linear models

- Data - Average genotypic value of 2000 F5 RILs for 40 cycles for each unique combination of factors or condition (40 (data points/condition) with 5 replicates per condition - 200)
- 60 unique conditions 2 (PT) x 3 (Selection Method) x 4 (Mating Design) x 5 (Migration Policies)
- Total number of data points - 60*5*40 = 12,000

```
## Load simresults data
data(package="SoyNAMSelectionMethods")

dim(Data)
```

```
## [1] 12000    10
```

```
colnames(Data)
```

```
##  [1] "Population"      "SelectionMethod" "BreedDesign"     "MigPolicy"
##  [5] "nQTL"            "heritability"    "Cond"            "Rep"
##  [9] "Cycle"           "G"
```

```
########################
```

Assess the best model from part 1 for normality and i.i.d (identical and independent distribution of residuals.

```
### Empty model with per cycle average genotypic value grouped across all the factor levels.

fit_Data_noCond <- nls(formulaExp,data=Data,start=list(alpha=0.1,beta=30))

### All 60 combinations of factors are given a unique ID in 'Cond' variable in the data table.
### Fixed effects: alpha and beta are fit only for the intercept (population average)
### Random effects: within group deviations are fit for each of the unique conditions with no nesting of groups.
### Starting values are derived from the fixed effects of nlsList fit.

##

qqnorm(resid(fit_Data_noCond),main="Q-Q Plot for Empty Model")
qqline(resid(fit_Data_noCond))
```

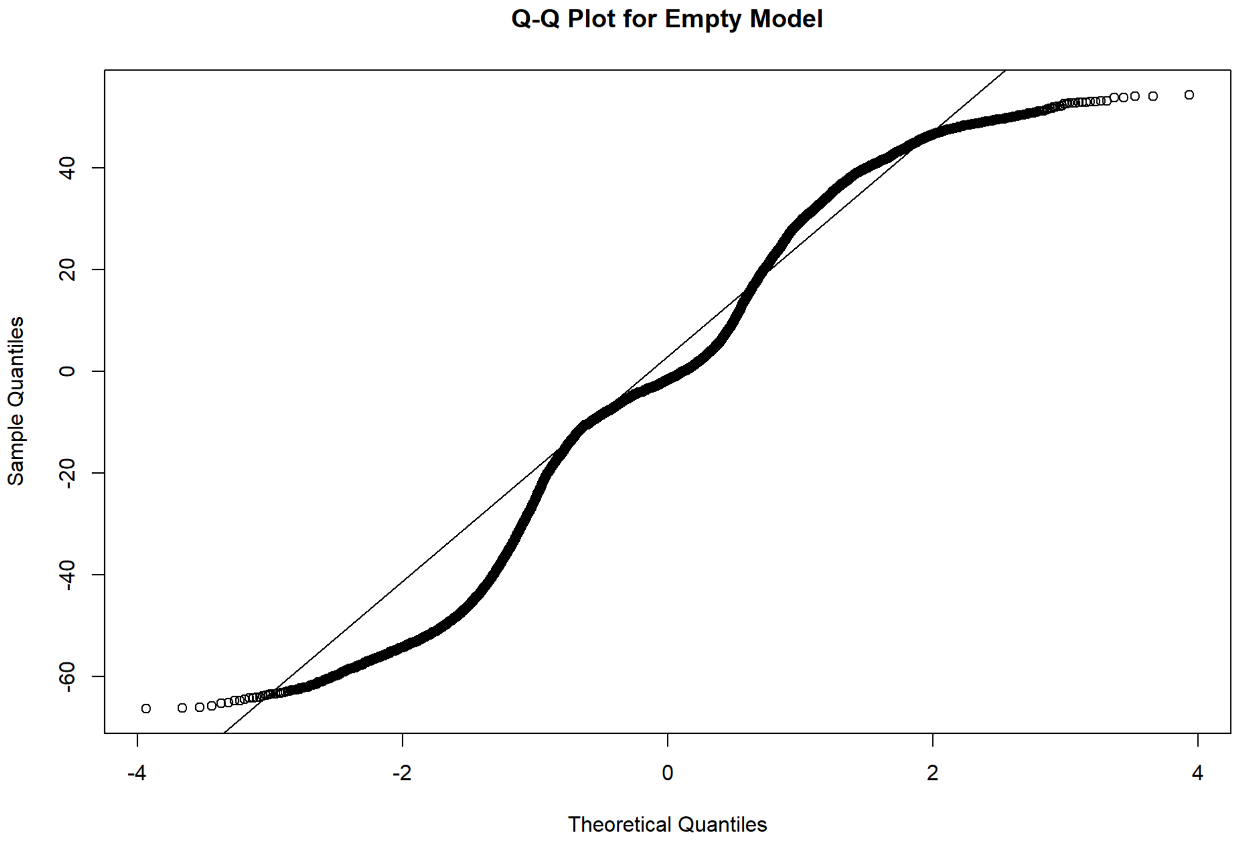

## Comment: qqnorm plot for ordinary residuals for M1 show

```
qqnorm(resid(fit_Data_nlme_Cond),main="Q-Q Plot for NLME_Conditi
ons")
qqline(resid(fit_Data_nlme_Cond))
```

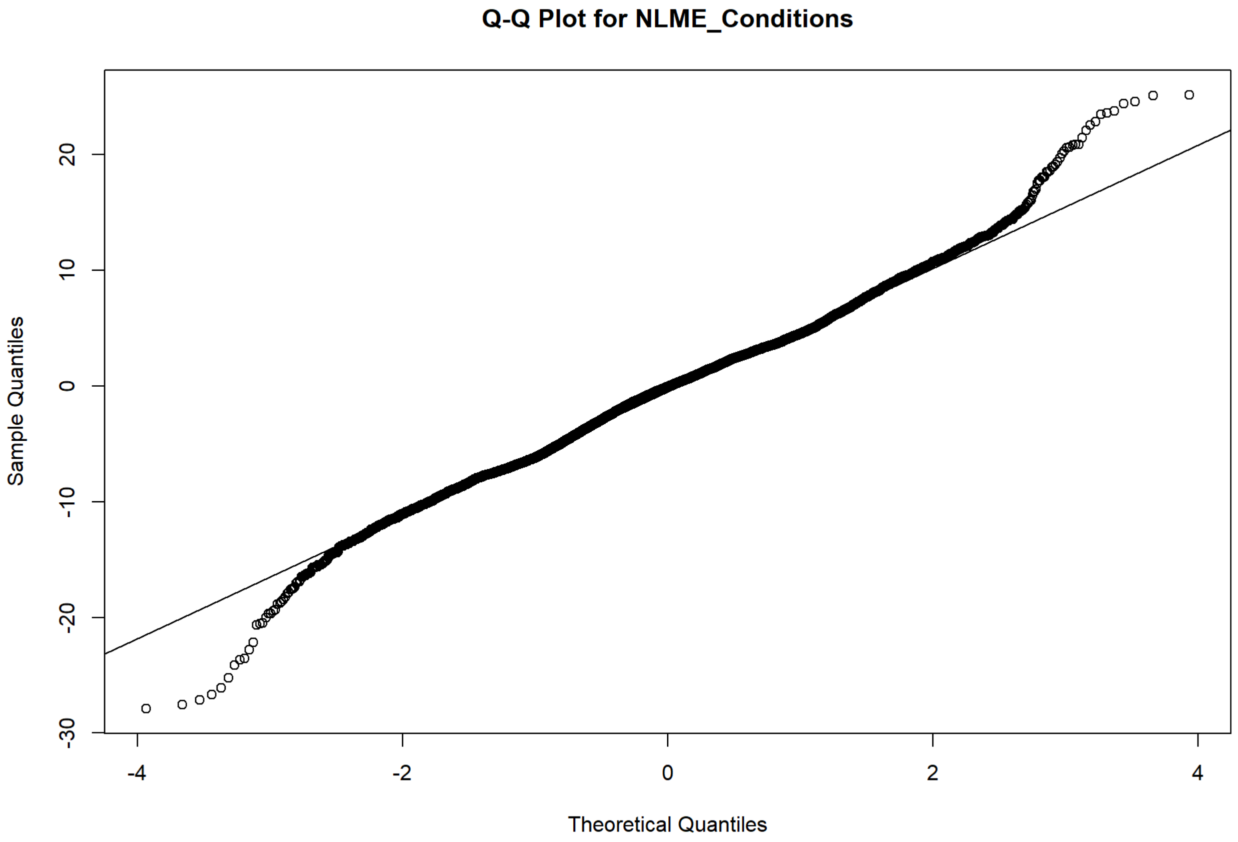

## Comment: qqnorm plot for ordinary residuals show

```
qqnorm(resid(fit_Data_nlme_Cond,type = "normalized"),main="Q-Q P
lot for Normalized NLME_Conditions ")
qqline(resid(fit_Data_nlme_Cond,type="normalized"))
```

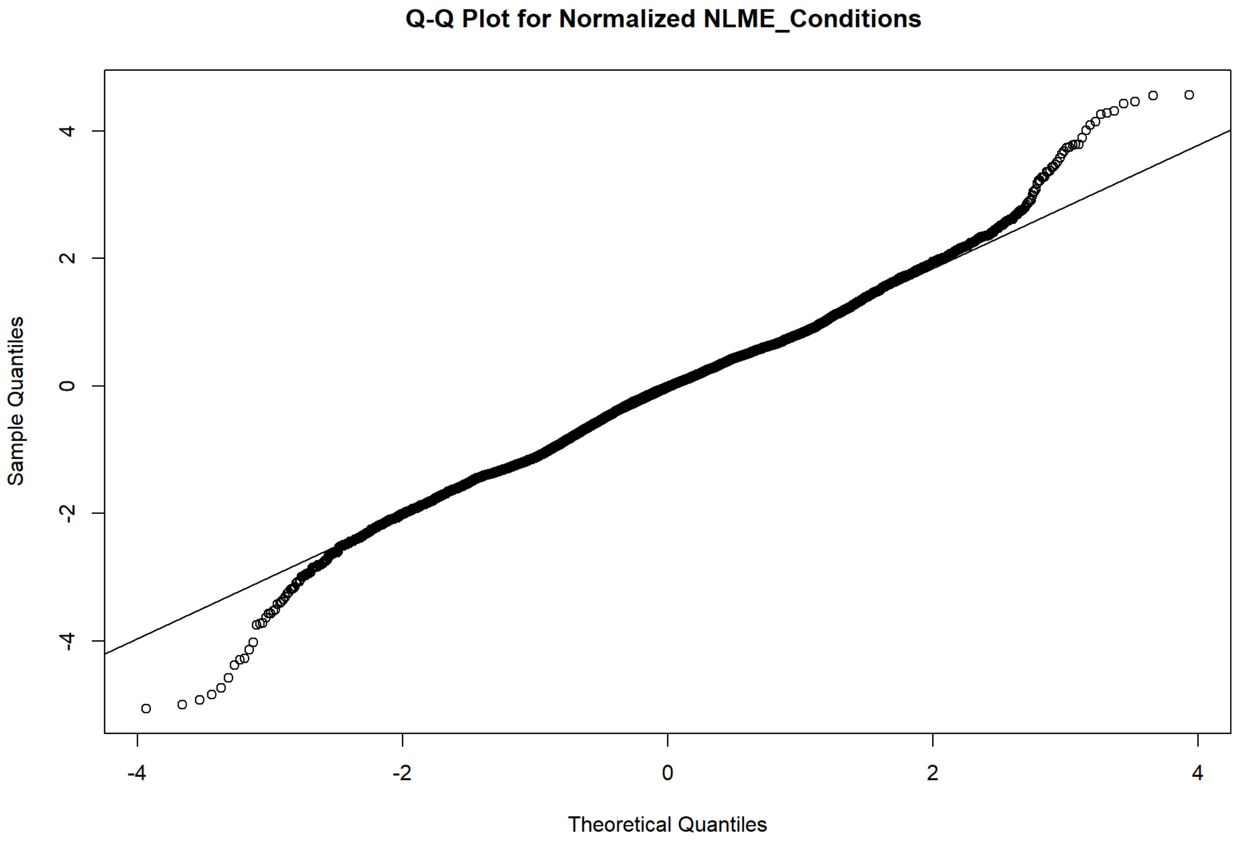

## Comment: qqnorm plot for normalized residuals show

```
plot(resid(fit_Data_nlme_Cond,type="normalized"),main="Normalize
d Residuals")
```

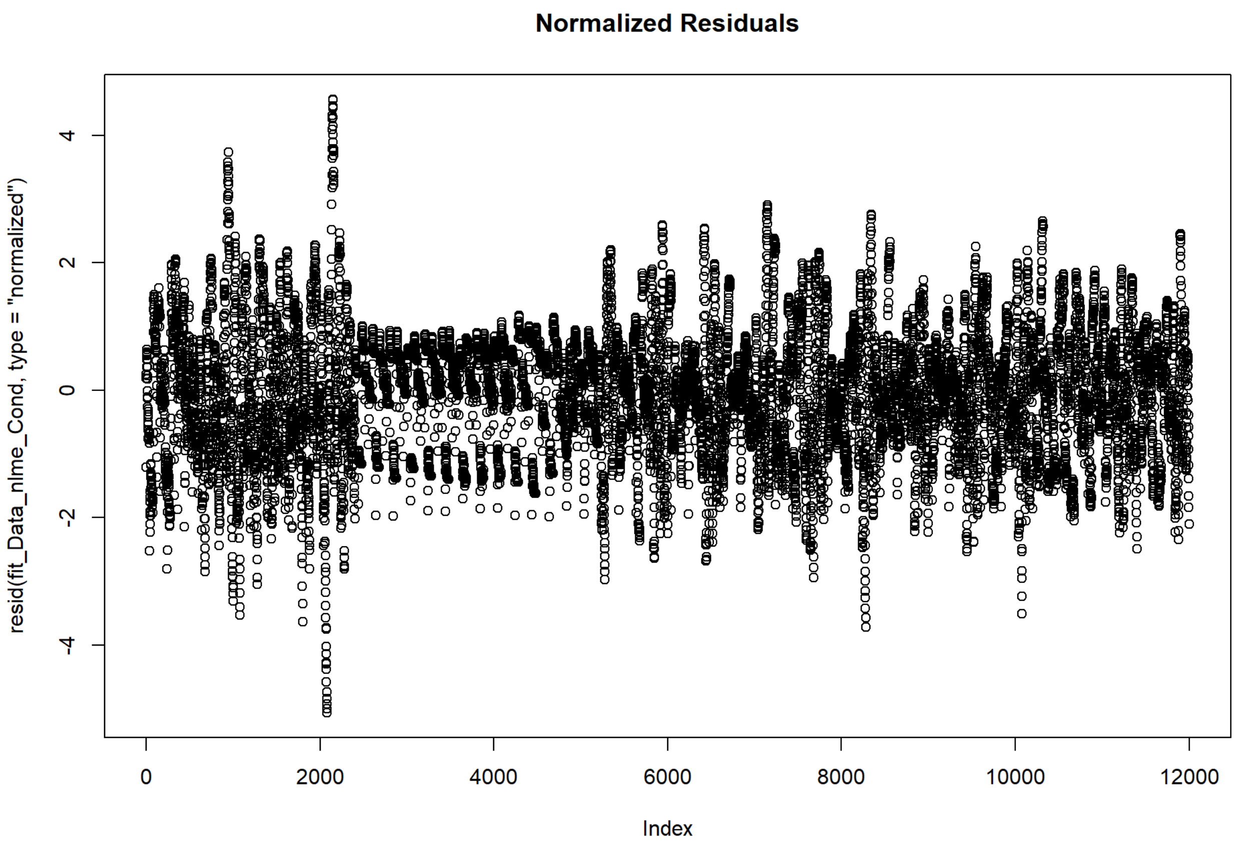

## Comment: Normalized residuals show even spread

```
plot(ranef(fit_Data_nlme_Cond),main="Random Effects")
```

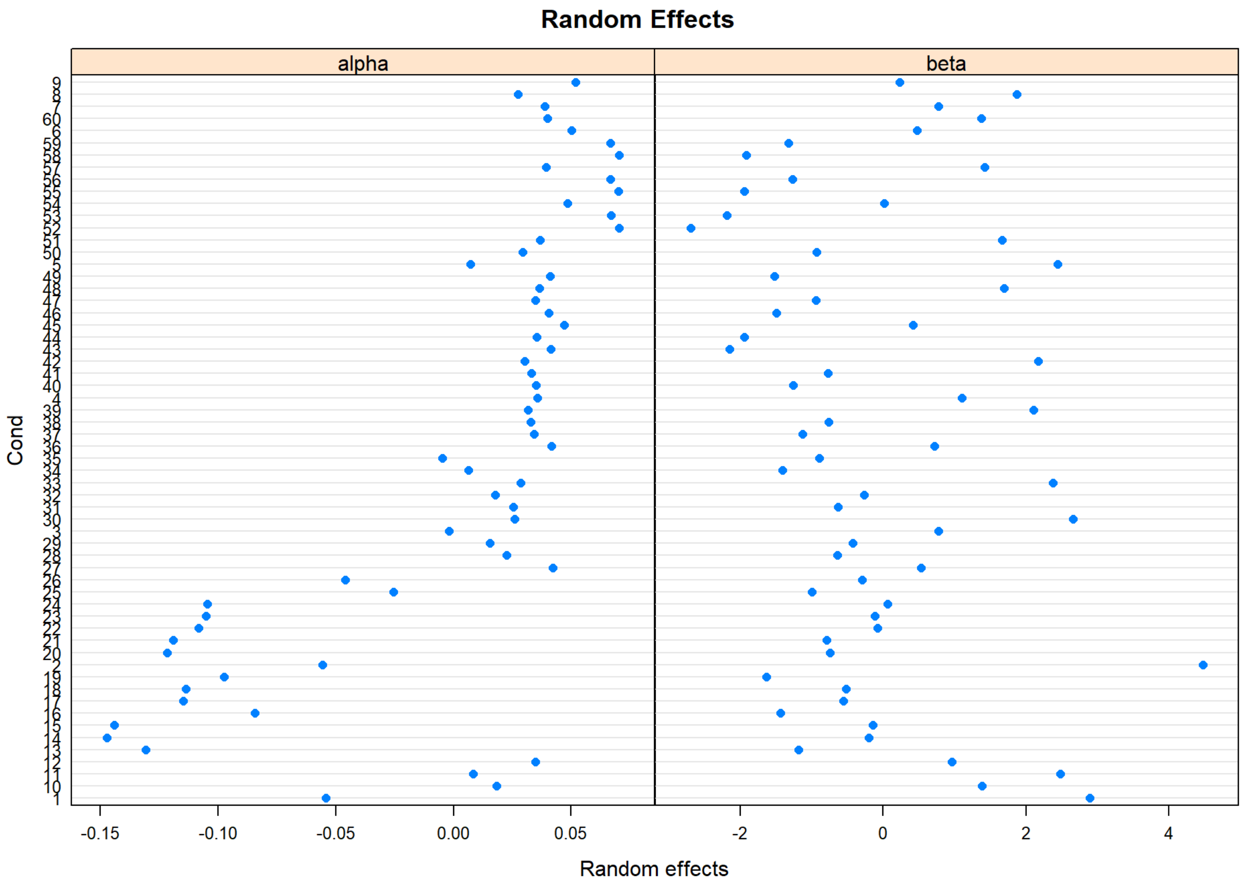


```
plot(ACF(fit_Data_nlme_Cond),main="Auto-correlation of Residuals")
```

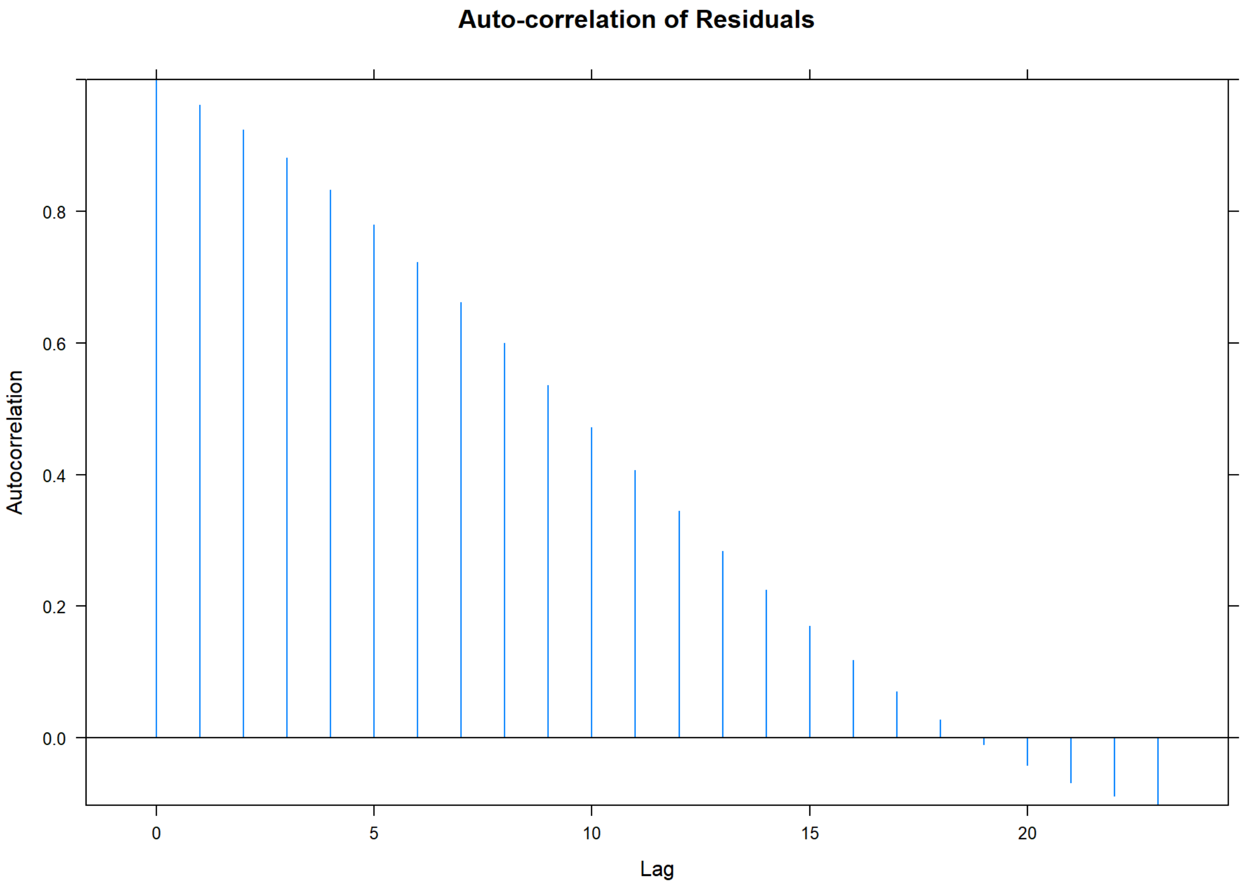

Temporal correlation structure modeling. Given recurrence relation of response variable across cycles of selection, there is strong auto-correlation among residuals violating the independence assumption. Modeling correlation structure using AR1 correlation structure doesn't reduce autocorrelation among time steps when grouped only on conditions, eventhough it results in significantly lower AIC score compared to model without any correlation structure.

NLME Fits with correlation structure: Update models M15 from Part-II to include correlation structure

```
####

#fit_Data_nlme_Cond_Corr <-update(fit_Data_nlme_Cond,correlation=corAR1(form= ~1|Cond),control=list(maxiter=200,msMaxIter=300,opt="nlminb"))

#fit_Data_nlme_Cond_Corr_VarIdent <- update(fit_Data_nlme_Cond_Corr,weights=varIdent(form=~1|Cond),control=list(maxiter=300,msMaxIter=400,opt="nlminb"))
```

ACF plots for M15 with correlation

```
###
plot(ACF(fit_Data_nlme_Cond_Corr))
```

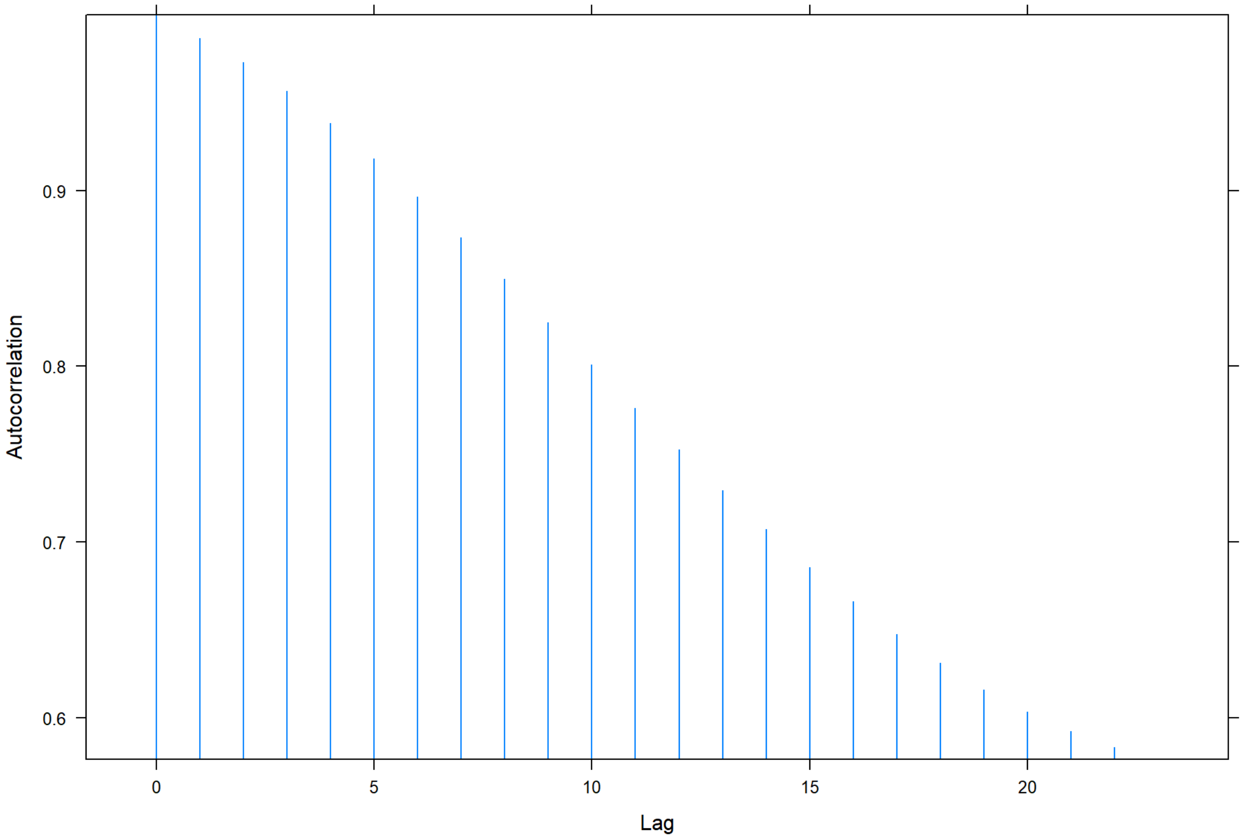

### Note that even though including cycle within conditions as a g rouping level in RI model removes most of the auto correlation in residuals, AIC value is larger than M31-Corr with only conditions as RI grouping factor

###

Variance component modeling: Given the use of 'cond' Id variable for grouping, M15 modeled with varIdent structure for unique variance for each of the conditions has the least AIC score. Modeling both variance and correlation structure is a time consuming process.So we plan to investigate further refinements to M15 with varCorr structure by including optimal fixed effects structure in the future for the purpose of fitting the optimal model and estimating interaction effects of factors.

###

```
plot(ACF(fit_Data_nlme_Cond_Corr_VarIdent))
```

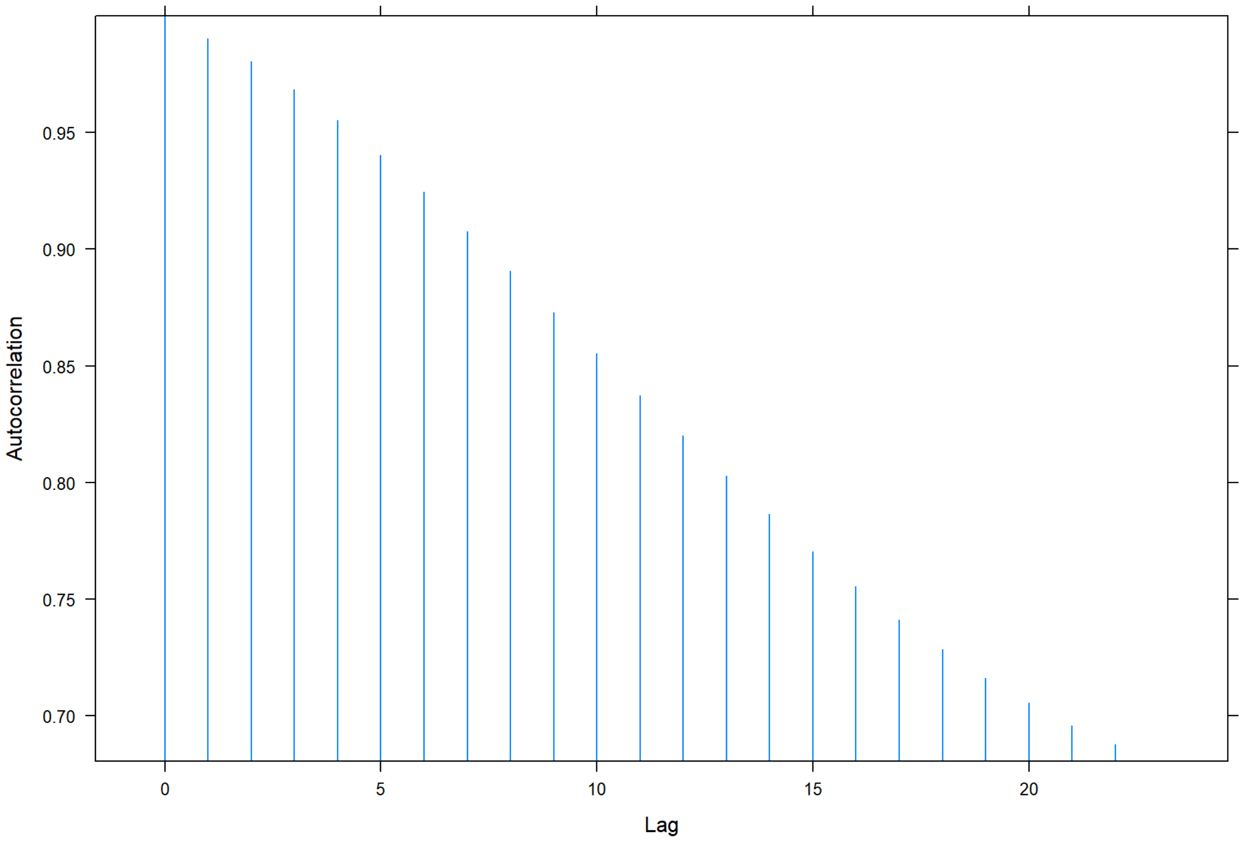


```
anova(fit_Data_nlme_Cond,fit_Data_nlme_Cond_Corr,fit_Data_nlme_Cond_Corr_VarIdent)
```

```
##                                     Model df      AIC      BIC    logLik   Test
## fit_Data_nlme_Cond                      1  6 75831.43 75875.79 -37909.72
## fit_Data_nlme_Cond_Corr                 2  7 42166.17 42217.92 -21076.08 1 vs 2
## fit_Data_nlme_Cond_Corr_VarIdent        3 66 40276.64 40764.56 -20072.32 2 vs 3
##                                    L.Ratio p-value
## fit_Data_nlme_Cond
## fit_Data_nlme_Cond_Corr          33667.26  <.0001
## fit_Data_nlme_Cond_Corr_VarIdent  2007.52  <.0001
```

ANOVA Table for NLME models M11-M15 with and without correlation structure for 40 cycles

```
knitr::kable(
ANOVA_PM_IM_SingleLevel[,3:6],booktabs=TRUE,
caption= 'NLME ANOVA Table'

)
```

NLME ANOVA Table

	df	AIC	BIC	logLik
fit_Data_nlme_Cond_PT	6	110715.11	110759.47	-55351.55
fit_Data_nlme_Cond_BD	6	111546.89	111591.25	-55767.45
fit_Data_nlme_Cond_SM	6	111671.50	111715.86	-55829.75
fit_Data_nlme_Cond_MP	6	92144.61	92188.97	-46066.30
fit_Data_nlme_Cond_PT_BD	6	109785.19	109829.55	-54886.60
fit_Data_nlme_Cond_PT_SM	6	110302.00	110346.36	-55145.00
fit_Data_nlme_Cond_PT_MP	6	92144.61	92188.97	-46066.30
fit_Data_nlme_Cond_BD_SM	6	111219.15	111263.51	-55603.57
fit_Data_nlme_Cond_BD_MP	6	84092.22	84136.58	-42040.11
fit_Data_nlme_Cond_SM_MP	6	89151.52	89195.88	-44569.76
fit_Data_nlme_Cond_PT_BD_SM	6	109353.62	109397.97	-54670.81
fit_Data_nlme_Cond_PT_BD_MP	6	84092.22	84136.58	-42040.11

	df	AIC	BIC	logLik
fit_Data_nlme_Cond_PT_SM_MP	6	89151.52	89195.88	-44569.76
fit_Data_nlme_Cond_BD_SM_MP	6	75831.43	75875.79	-37909.72
fit_Data_nlme_Cond_PT_BD_SM_MP	6	75831.43	75875.79	-37909.72
fit_Data_nlme_Cond	6	75831.43	75875.79	-37909.72
fit_Data_nlme_Cond_PT_BD_SM_Corr	7	72792.40	72844.15	-36389.20
fit_Data_nlme_Cond_PT_BD_MP_Corr	7	50022.47	50074.21	-25004.23
fit_Data_nlme_Cond_PT_SM_MP_Corr	7	57736.59	57788.34	-28861.30
fit_Data_nlme_Cond_BD_SM_MP_Corr	7	42166.17	42217.92	-21076.08
fit_Data_nlme_Cond_PT_BD_SM_MP_Corr	7	42166.17	42217.92	-21076.08
fit_Data_nlme_Cond_Corr	7	42166.17	42217.92	-21076.08
fit_Data_nlme_Cond_Corr_VarIdent	66	40276.64	40764.56	-20072.32

EOF
