# Supplementary material for: Strategies to Assure Optimal Trade-Offs Among Competing Objectives for the Genetic Improvement of Soybean
Source: Front Genet. 2021 Sep 24;12:675500. doi: 10.3389/fgene.2021.675500 (PMC8497982; doi:10.3389/fgene.2021.675500)
Supplement: Supplementary file 3 [file Data_Sheet_3.docx]

**Supplementary File 1**

In the genomic mating (GM) method (Akdemir and Sanchez 2016), the objective is to minimize the expected rate of inbreeding w.r.t. a mating plan that is defined in the parentage matrix P_32_, subject to a constraint that the risk of a mating plan meets a given threshold of rho (Eqn 1).

In this case, the mating plan is a set of mate pairs and their contributions. For example, if there are 10 lines (N_p_) in a selection population and 100 lines (N_c_) in a progeny population, the mating plan is a set of 10 pairs of lines. If the size of the progeny population is 100, then the set of 10 mate pairs will be used in various proportions to obtain a population of 100 progeny.

The risk of a mate pair is related to the usefulness of a cross that captures the expected benefit and variance of progeny from a mate pair (Eqn 2). The Risk of a mating plan is the sum of risk measures of all the mate pairs in a plan.

minimize Inbreeding (P_32_) = 1Nc’ (P_32_GP_32_’ +D_3_) 1Nc - Eqn 1

w.r.t. P_32_

subject to Risk (λ1,P_32_) = ρ

- P_32_- Parentage matrix / Mating Plan
- G – Genomic Relationship matrix
- D_3_ – A matrix of Mendelian sampling term and is related to the inbreeding coefficient of the mate pair.
- λ1 – parameter that controls for marker allele heterozygosity weighted by marker effects
- Risk of a mate pair = sum of EBV of progeny + λ1 SD (EBVs of progenies from a mate pair)
- Risk of P_32_ (mating plan) is the sum of risk of mate pairs in that set

Risk of a mate pair = sum of EBV of progeny + λ1 *Standard Deviation (EBVs of progenies)

- Eqn 2

, where EBV corresponds to the Expected Breeding Value of progeny from a mate pair.

The objective function is minimized w.r.t. to a mating plan. A genetic algorithm is used to search for pareto-optimal solutions in the form of mate pairs and their contributions to the progeny generation (parameters of the genetic algorithm are provided in the main section).
