# Supplementary material for: Strategies to Assure Optimal Trade-Offs Among Competing Objectives for the Genetic Improvement of Soybean
Source: Front Genet. 2021 Sep 24;12:675500. doi: 10.3389/fgene.2021.675500 (PMC8497982; doi:10.3389/fgene.2021.675500)
Supplement: Supplementary file 6 [file Data_Sheet_6.docx]

**Supplementary Figures**


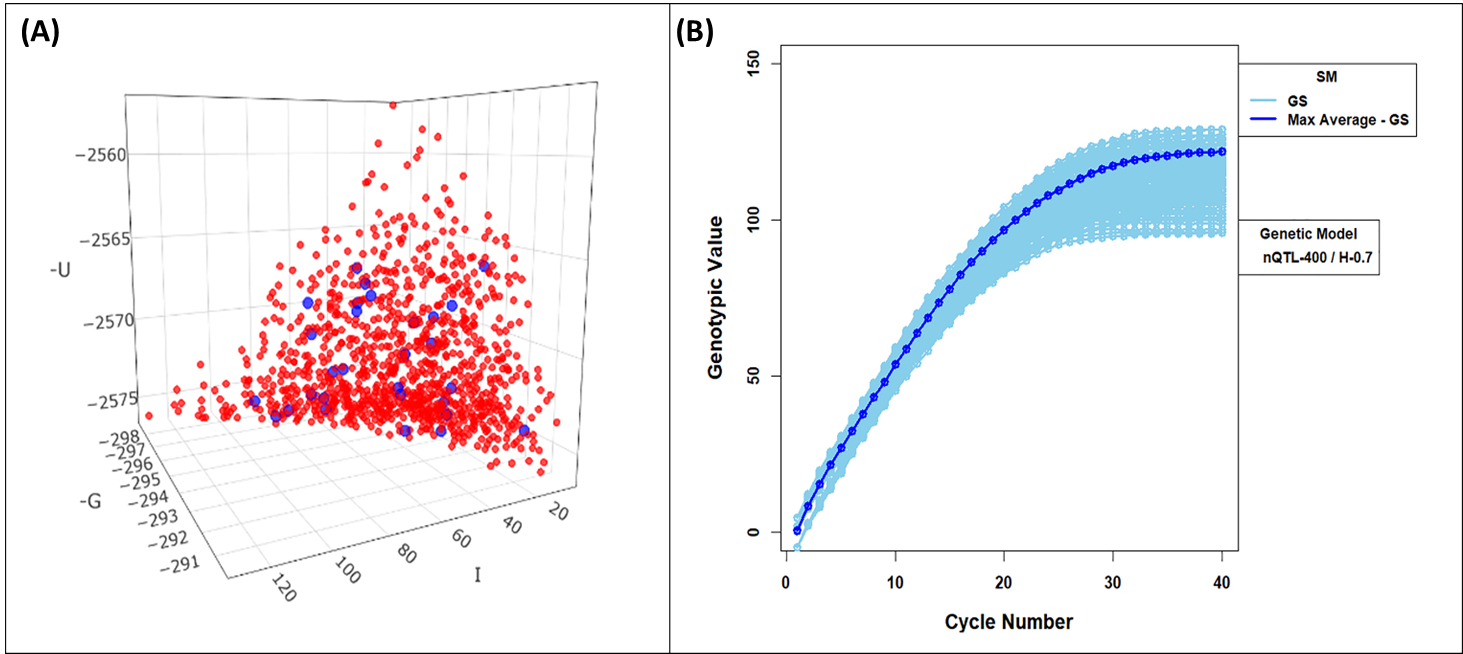


**Supplementary Figure 1** The left panel (A) is a plot of pareto-optimal solutions creating a frontier surface for values of genetic gain (-G), Inbreeding (I) and Usefulness (-U). For this particular plot, the Genomic Mating (GM) method is applied to F5 derived lines selected in one of the family islands using Genomic Selection (GS). Each point is an optimal solution corresponding to a set of 10 pairs of F5 derived lines selected as parental lines within one island. A similar set of pareto-optimal solutions are selected for all 20 family islands. Out of all the pareto-optimal solutions (red circles) on the frontier surface, 30 (blue circles) consisting of 10 optimal solutions with high G, 10 optimal solutions with low G and 10 optimal solutions with median G are selected. The solution pairs in each of the 30 selected sets are crossed and the recurrent selection cycle is iterated for 40 cycles for each of the 30 sets.

The right panel (B) is a plot of genotypic values, when the selected set of 30 solutions are crossed and the process of selection and crossing using the GM method is iterated for 40 cycles of recurrent selection for all 20 family islands. The thick blue curves represent parameters with maximum limits of response for GS. The genotypic values represent responses in recurrent GS with 400 QTL responsible for 70% of phenotypic variability in the founding set of F5 derived lines and top 10% selected fraction with bi-directional migration of two migrants every other cycle with “Fully Connected” (FC) migration policy.


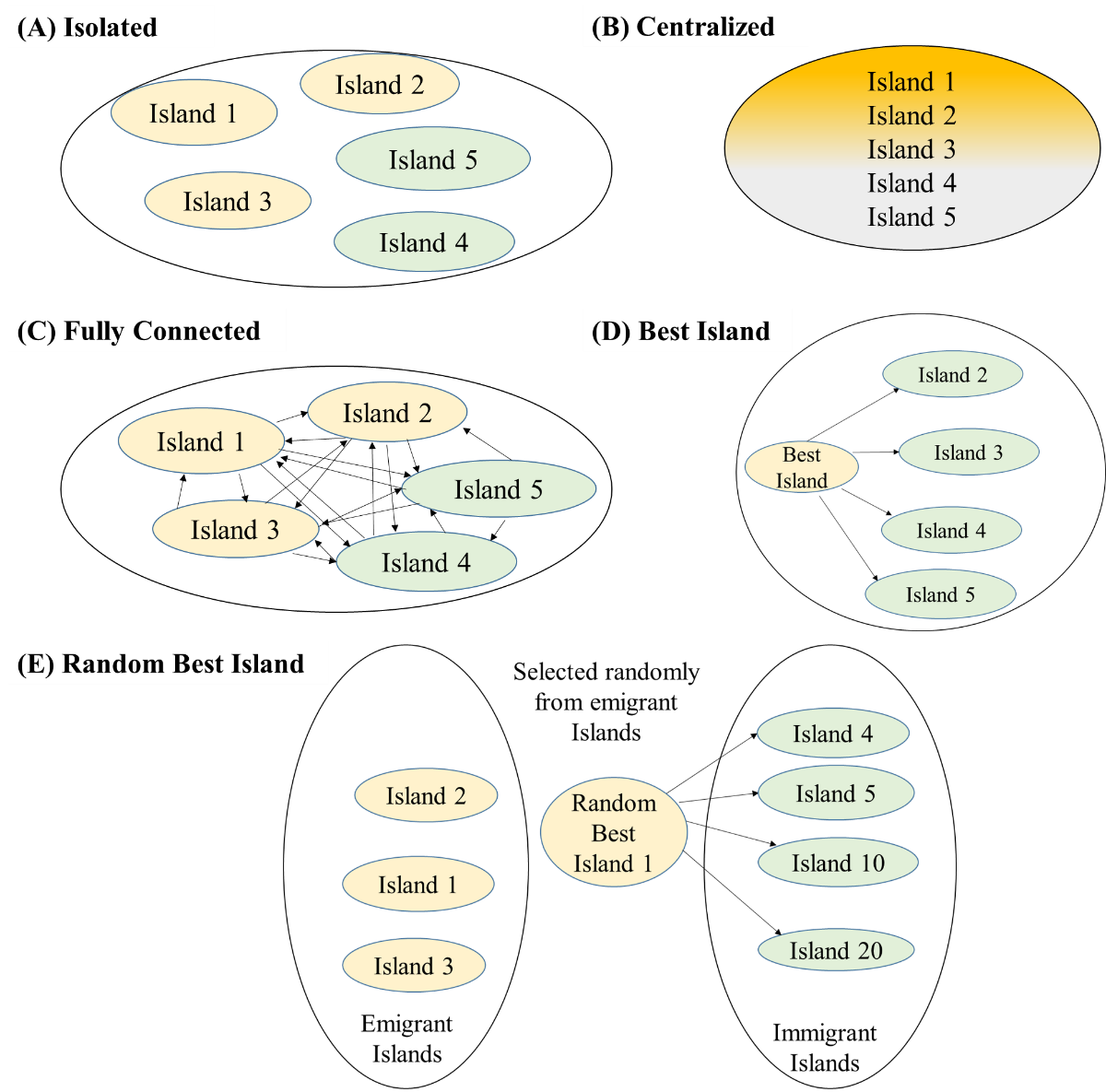


**`**

**Supplementary Figure 2 Schematic Representation of Island Topology / Migration** **Policies:** (A) Isolated islands, where the islands are not connected and there is no exchange of lines**,** (B) Centralized island population, where there is no separation among islands and the lines in the islands are pooled into one population, (C) “Fully Connected” island topology**,** where every island is connected to every other island and lines migrate from emigrant islands with high values to randomly selected immigrant islands, (D) **“**Best Island” topology, where emigrant lines are selected from the island with the largest criterion value used for selection and emigrants migrate to no more than 10 islands. If the migration event is bi-directional, the emigrant island also receives immigrants, (E) “Random Best” policy, where, an emigrant island is selected randomly from a set of 10 islands with large genotypic values and emigrants migrate to no more than 10 family islands.


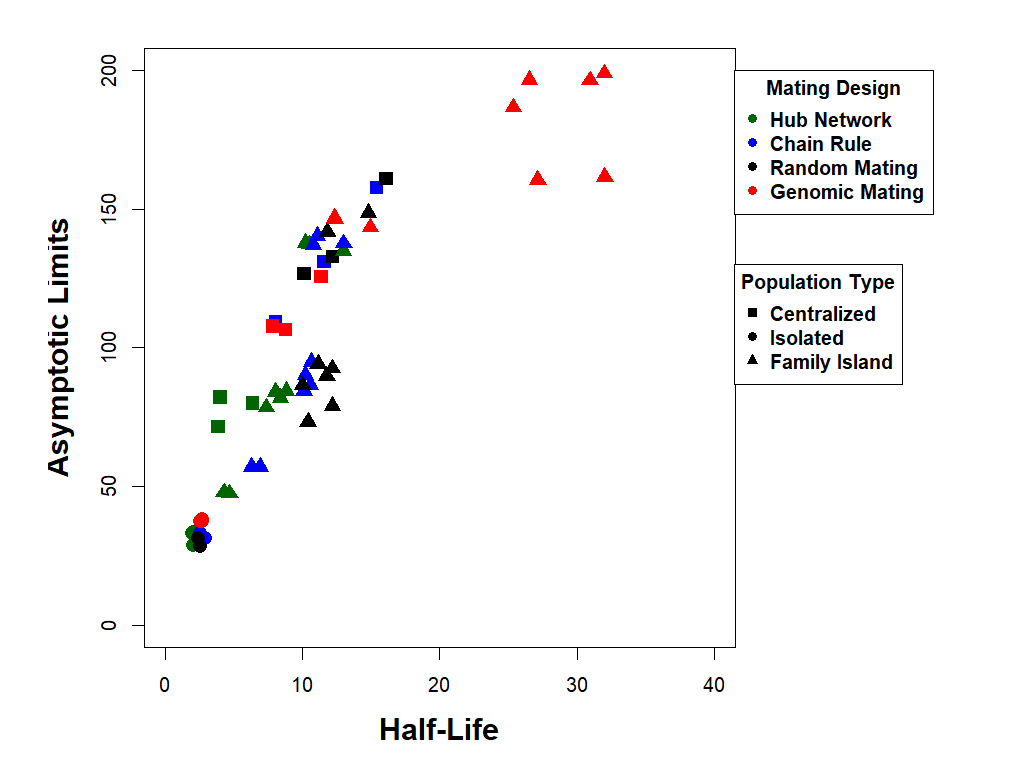


**Supplementary Figure 3 Asymptotic limits and Half-life of Recurrent Selection for the 60 methods**. Half-life is plotted on the x-axis and asymptotic limits on the y-axis. The cluster of circles correspond to isolated (IS) methods. The square and triangular points correspond to centralized (CE) and family island selection (IM) methods respectively. The cluster of red triangular points in the top-right corner with half-life above 25 and asymptotic limits above 160 correspond to island selection with GM method combined with Best Island and Random Best migration policies (IM-GM-BI & IM-GM-RB with PS, GS and WGS). These points are likely to be over-estimates, as the genotypic values for 40 cycles of selection fall on the linear region of the response curve. Simulations of additional cycles of selection are likely to provide more accurate estimates for these methods with larger half-life and asymptotic limits compared to all other methods, but lesser than the current estimates. All simulations use 400 simulated QTL responsible for 70% of phenotypic variability. Top 10% of the lines are selected from centralized and island populations as parental lines to be crossed using Hub Network (HN- green), Chain Rule (CR-blue), Random Mating (RM -black) and Genomic Mating (GM-red) designs. Selection Methods included PS-Phenotypic Selection (PS), Genomic Selection (GS), and Weighted Genomic Selection (WGS) with Jannink weighting function. Migration policies included “Isolated” (IS), “Centralized” (CE), “Family Island” (FI), “Best Island” (BI), “Random Best” (RB), and “Fully Connected” (FC) policies with bi-directional migration of two migrants every other cycle of selection.


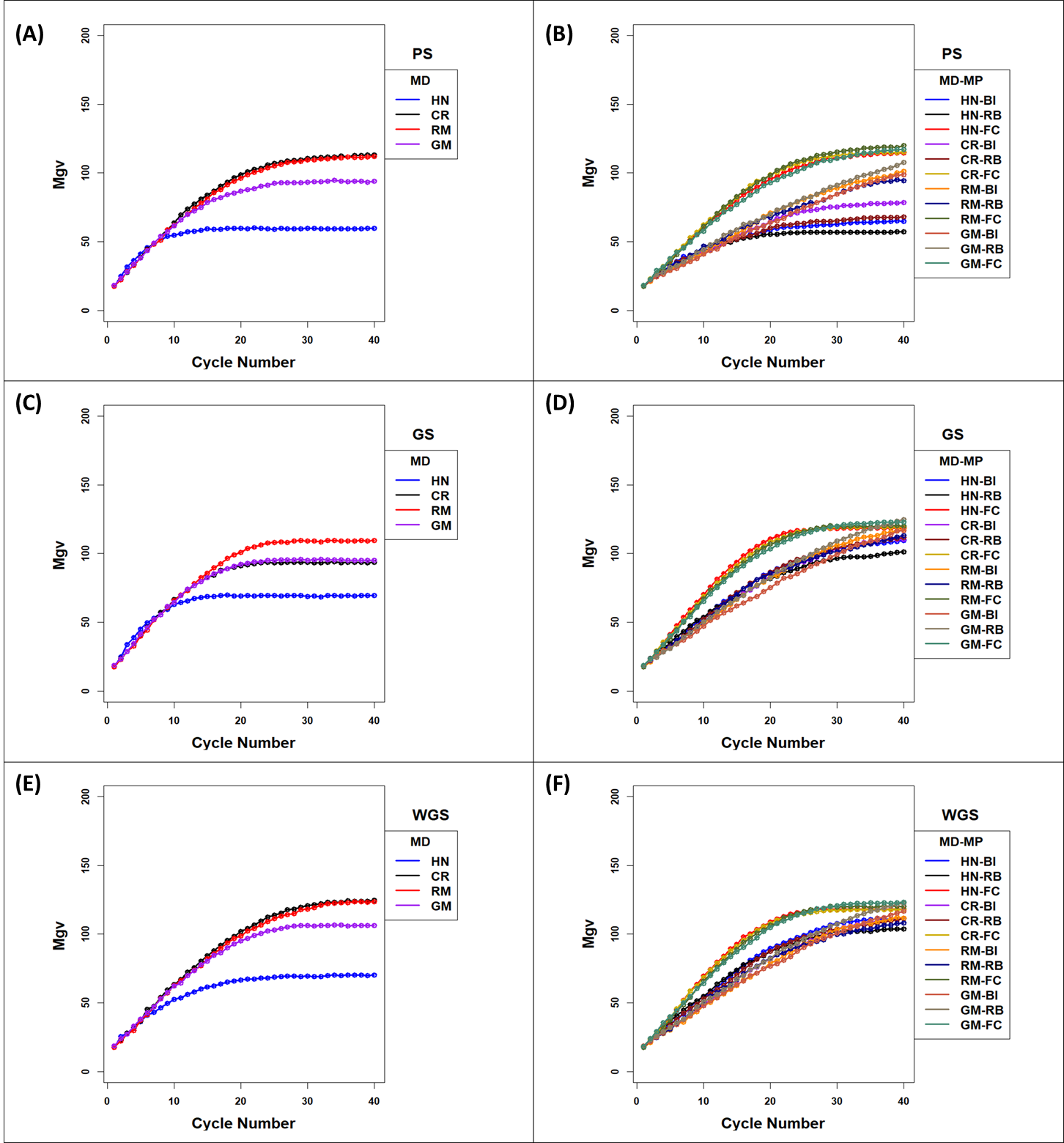


**Supplementary Figure 4** Maximal Genotypic Values (Mgvs) across 40 cycles of recurrent selection on centralized (A, C, E) and family island (B, D, F) populations, using Phenotypic Selection (PS- A, B), Genomic Selection (GS- C, D) and Weighted Genomic Selection (WGS- E,F) and four mating designs: Hub Network (HN), Chain Rule (CR), Random Mating (RM), and Genomic Mating (GM). Standardized genotypic responses are represented from a simulated genetic architecture consisting of 400 additive QTL uniformly distributed throughout the genome and responsible for 70% of phenotypic variability. Ten percent of lines are selected to be used in crosses. Migration policies included the Best Island (BI), Random Best (RB), and Fully Connected (FC) island topologies with bi-directional migration of two migrants every other cycle.


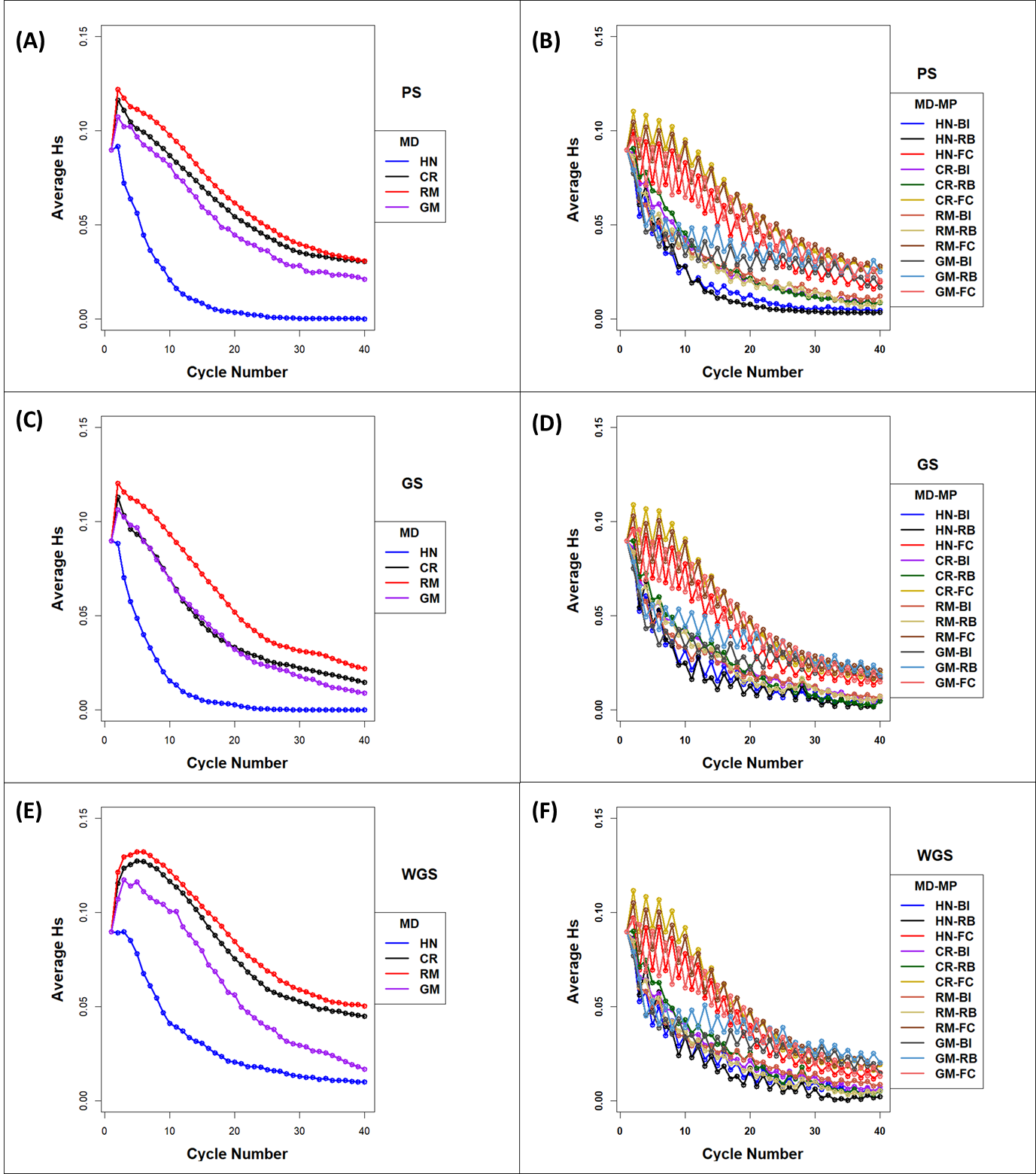


**Supplementary Figure 5** Average Expected Heterozygosity (Hs) in centralized (A, C, E) and family island populations (B, D, F). Hs in centralized selection with PS (A, B), GS (C, D) and WGS (E, F) for the four mating designs including Hub Network (HN), Chain Rule (CR), Random Mating (RM), and Genomic Mating (GM) with a selection intensity of top 10% selected fraction. GP models are updated every cycle in GS and WGS. Selection methods included Phenotypic Selection (PS), Genomic Selection (GS), and Weighted Genomic Selection (WGS) with Jannink weighting function. Migration policies included the Best Island (BI), Random Best (RB), and Fully Connected (FC) island topologies with bi-directional migration of two migrants every other cycle of selection.


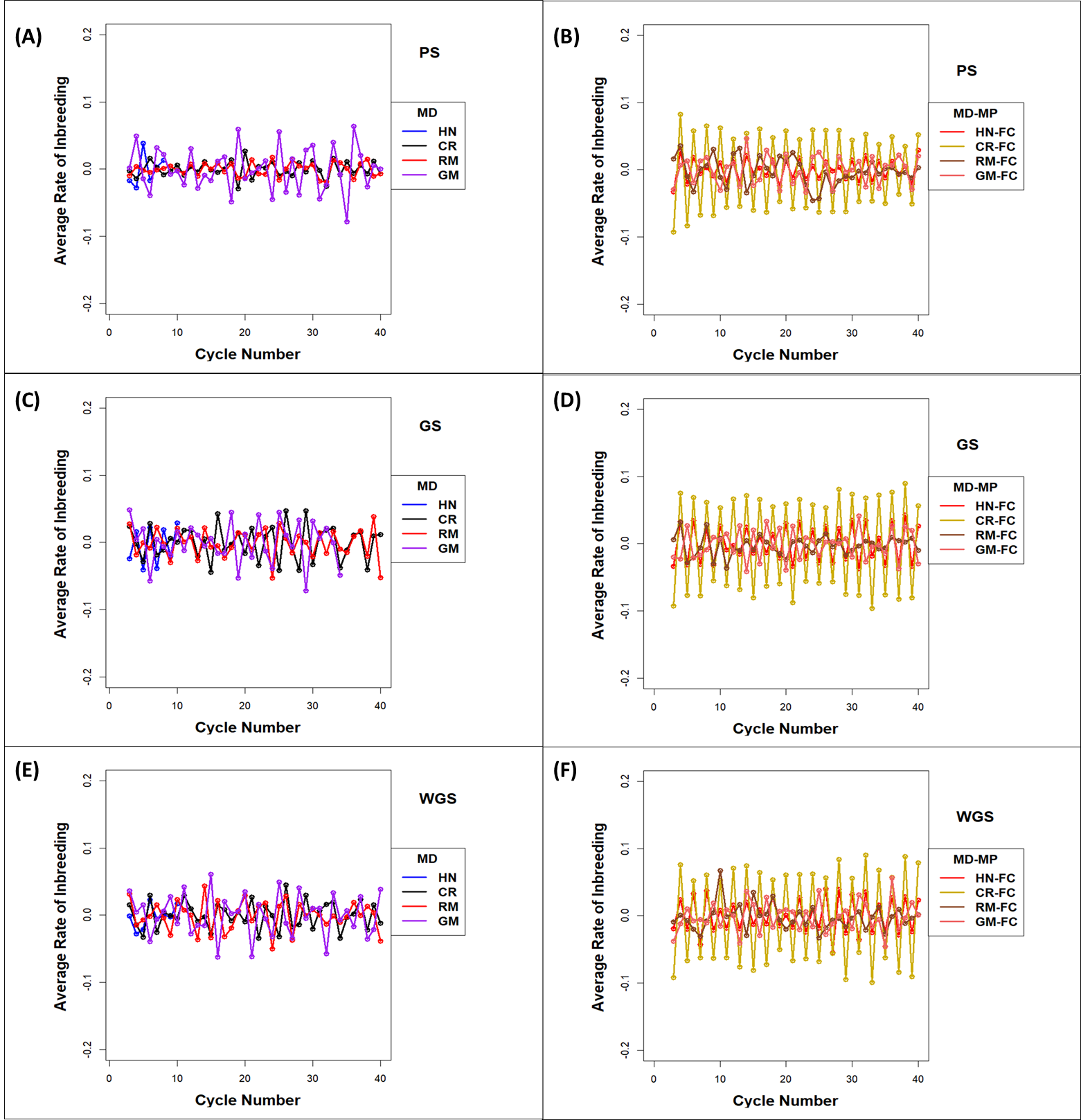


**Supplementary Figure 6** Average Rate of Inbreeding for centralized (A, C, E) and family island populations (FI) (B, D, F) with Fully Connected (FC) migration policy using Phenotypic Selection (PS - A, B), Genomic Selection (GS - C, D) and Weighted Genomic Selection (WGS -E, F) for the four mating designs including Hub Network (HN), Chain Rule (CR), Random Mating (RM), and Genomic Mating (GM) with a selection intensity of top 10% selected fraction. GP models are updated every cycle in GS and WGS. Migration rules included bi-directional migration of two migrants every other cycle of selection.


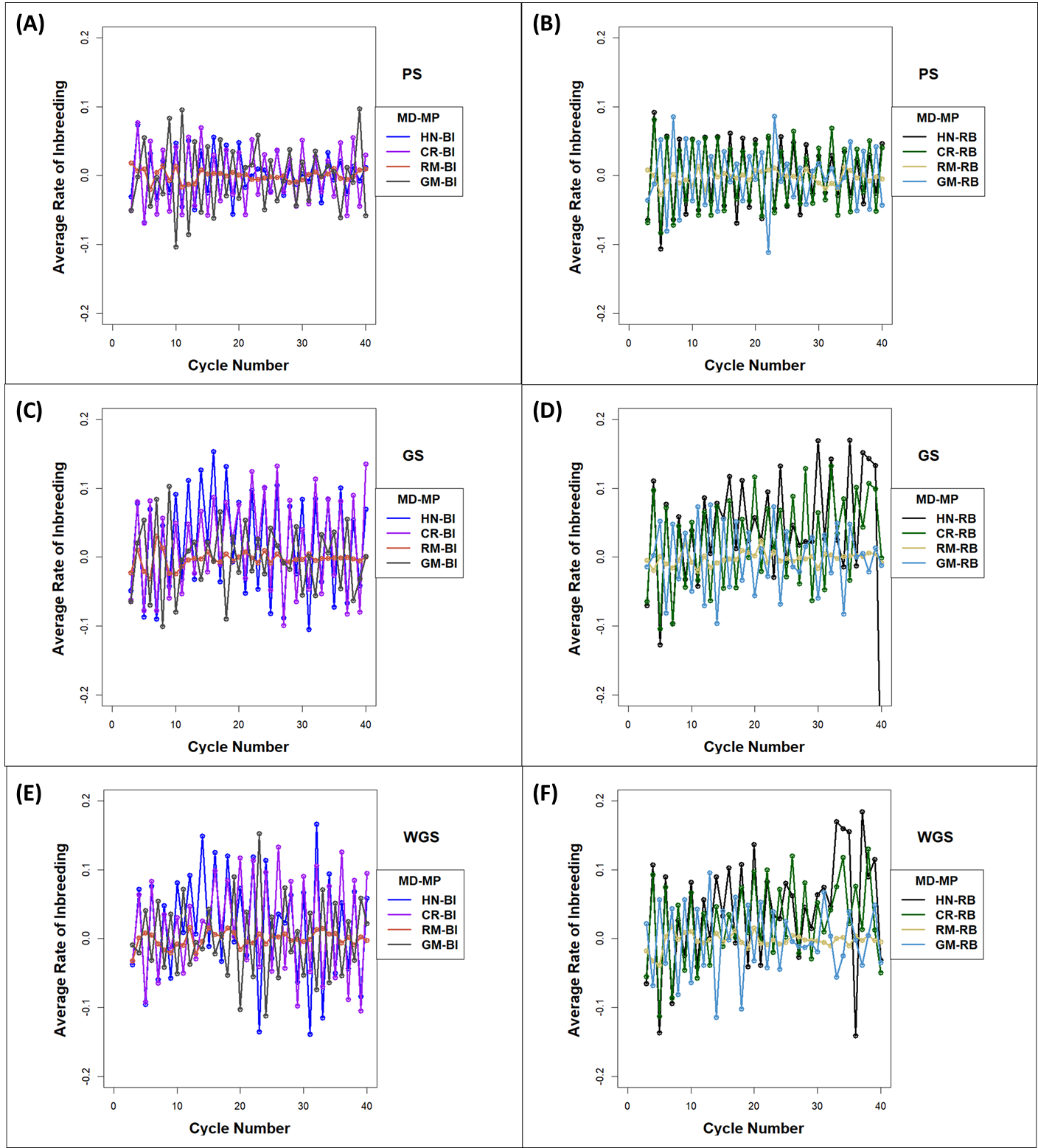


**Supplementary Figure 7** Average Rate of Inbreeding for island selection with BI and RB migration policies using Phenotypic Selection (PS) (A, B), Genomic Selection (GS) (C, D) and Weighted Genomic Selection (WGS) (E, F) for the four mating designs including the Hub Network (HN), Chain Rule (CR), Random Mating (RM), and Genomic Mating (GM) with top 10% selected fraction. GP models are updated every cycle in GS and WGS. Selection methods included Phenotypic Selection (PS), Genomic Selection (GS), and Weighted Genomic Selection (WGS) with Jannink weighting function. Migration policies included the Best Island (BI), Random Best (RB), and Fully Connected (FC) island topologies with bi-directional migration of two migrants every other cycle of selection.

.


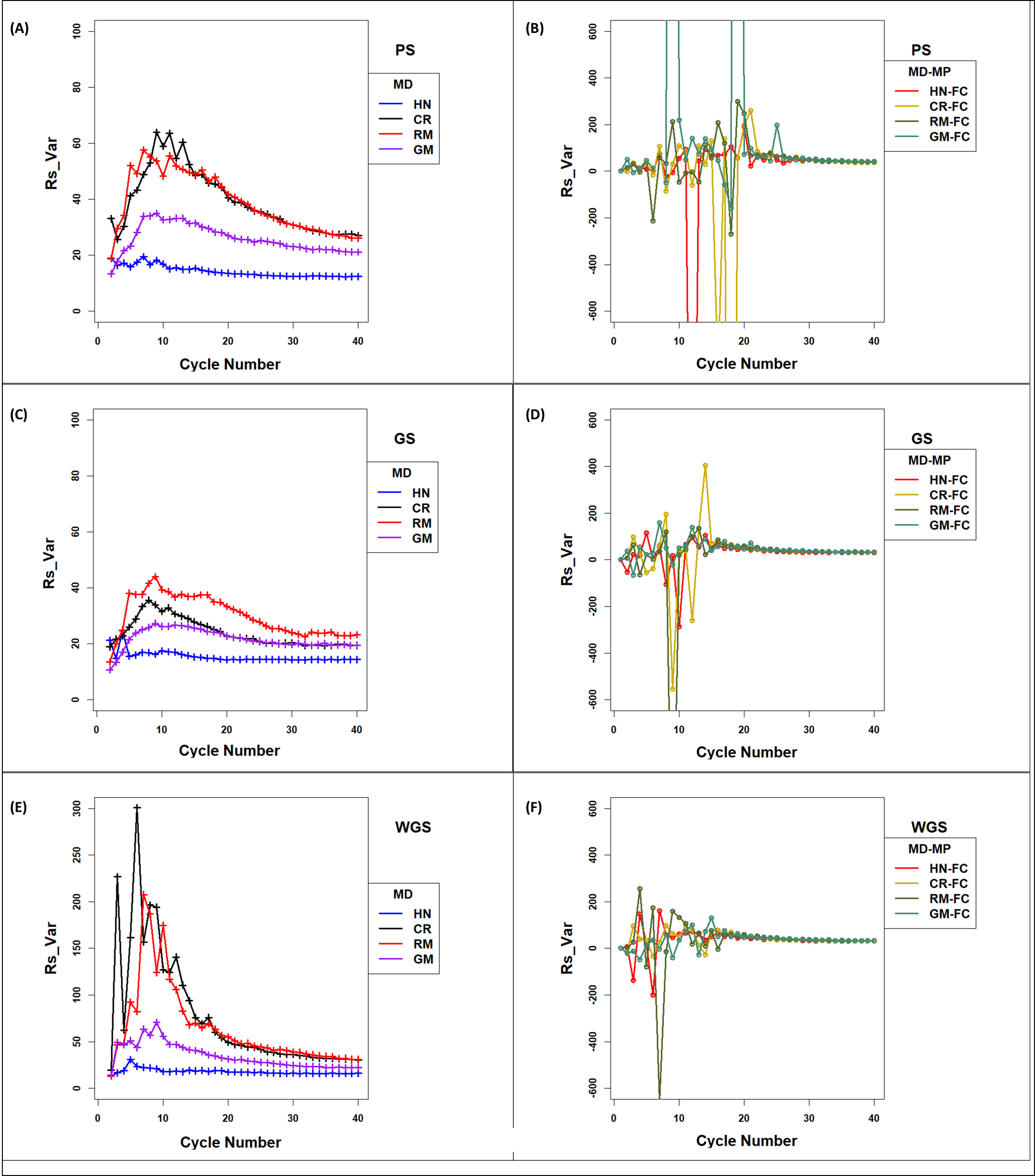


**Supplementary Figure 8 Response Standardized to Change in Genotypic Variance** (**Rs_Var – Eqn 9)** across 40 cycles of recurrent selection on centralized (A, C, E) and island (B, D, F) populations with a Fully Connected (FC) migration policy, using Phenotypic Selection (PS - A, B), Genomic Selection – (GS - C, D) and Weighted Genomic Selection (WGS -E, F) and four mating designs: Hub Network (HN), Chain Rule (CR), Random Mating (RM), and Genomic Mating (GM). Ten percent of lines in the population are selected for mating. The genetic architecture in the initial simulated founder lines consisted of 400 additive QTL uniformly distributed throughout the genome and expressed broad sense heritability of 0.7 on an entry mean basis. Selection methods included Phenotypic Selection (PS), Genomic Selection (GS), and Weighted Genomic Selection (WGS) with Jannink weighting function. Migration policies included the Best Island (BI), Random Best (RB), and Fully Connected (FC) island topologies with bi-directional migration of two migrants every other cycle of selection.


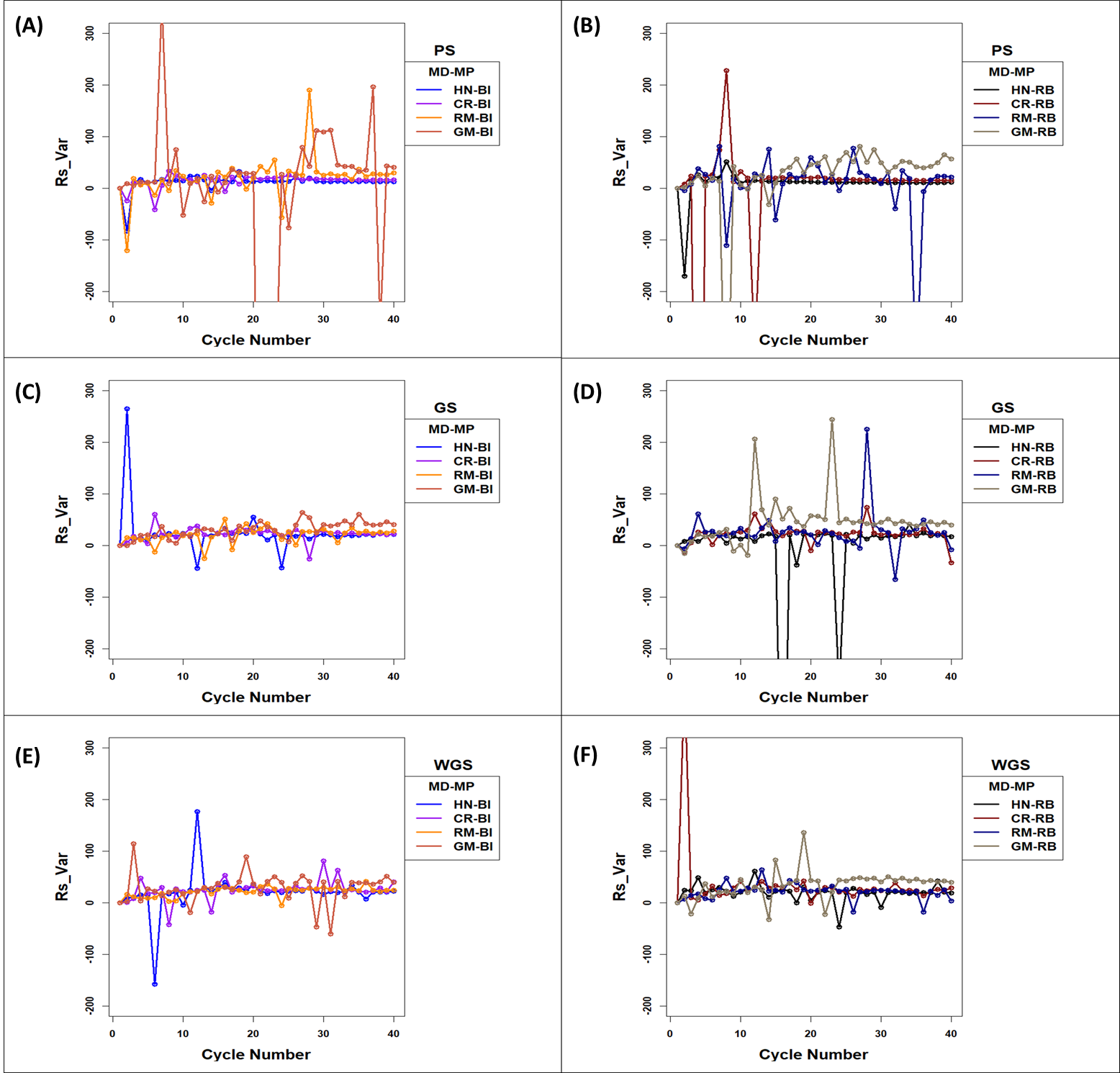


**Supplementary Figure 9 Response Standardized to Change in Genotypic Variance** (**Rs_Var – Eqn 9)** across 40 cycles of recurrent selection on island populations with Best Island (BI) (A, C, E) and Random Best (RB) migration policies (B, D, F), using Phenotypic Selection (PS - A, B), Genomic Selection – (GS - C, D) and Weighted Genomic Selection (WGS - E, F) and four mating designs: Hub Network (HN), Chain Rule (CR), Random Mating (RM), and Genomic Mating (GM). Ten percent of lines in the population are selected for mating. The genetic architecture in the initial simulated founder lines consisted of 400 additive QTL uniformly distributed throughout the genome and expressed a broad sense heritability of 0.7 on an entry mean basis. Migration rules included bi-directional migration of two migrants every other cycle of selection.


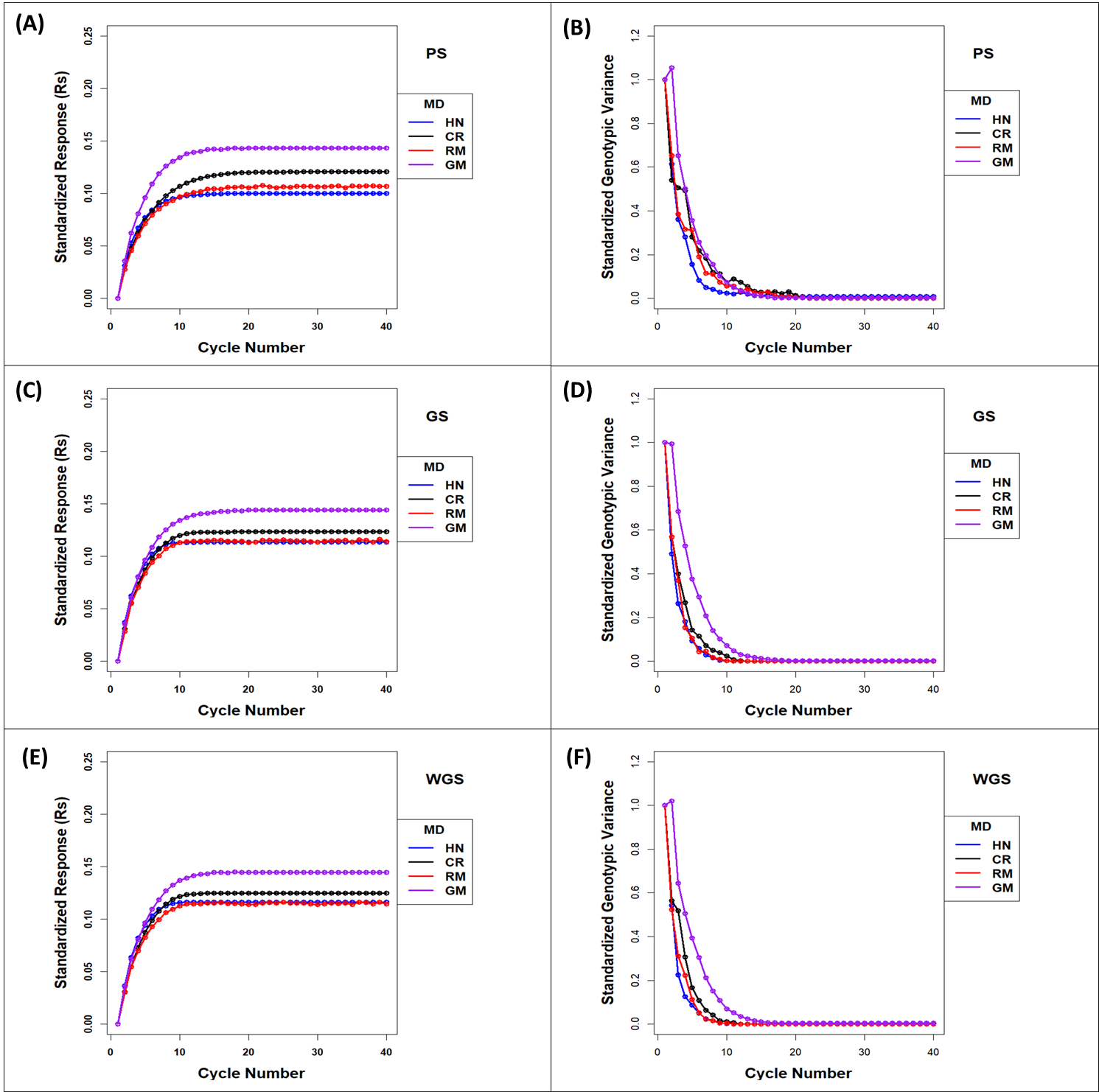


**Supplementary Figure 10** Standardized Response (Rs) (A, C, E) and Standardized Genotypic Variance (Sgv) (B, D, F) in isolated island populations for Phenotypic Selection (PS) (A, B), Genomic Selection (GS) (C, D) and Weighted Genomic Selection (WGS) (E, F) for the four mating designs including Hub Network (HN), Chain Rule (CR), Random Mating (RM), and Genomic Mating (GM). Standardized genetic response are provided for simulations with 400 simulated QTL responsible for 70% of phenotypic variability. Ten percent of lines are selected from isolated island populations as parental lines for mating. Genetic variance is standardized to the average genetic variance in founder populations in cycle ‘0’. Average island genetic variance refers to genetic variance within families averaged across 20 families. GP models are updated every cycle with training data from all prior cycles of selection. Migration rules included bi-directional migration of two migrants every other cycle of selection.
