# Supplementary material for: Strategies to Assure Optimal Trade-Offs Among Competing Objectives for the Genetic Improvement of Soybean
Source: Front Genet. 2021 Sep 24;12:675500. doi: 10.3389/fgene.2021.675500 (PMC8497982; doi:10.3389/fgene.2021.675500)
Supplement: Supplementary file 7 [file Data_Sheet_7.docx]

**Supplementary Table**

| **Supplementary Table 1 Weighting Functions for Weighted Genomic Selection** | | | |
| --- | --- | --- | --- |
| **Weighting Method** | **Function** | **Variables** | **Reference** |
| Jannink Method of weighting low frequency alleles with favorable effects. |  | c_i_ – criterion value for ith individual  x_ij_ – genotype matrix for n- individuals X p-markers  β_j_- effect of marker j  p_j_ – frequency of favorable allele at locus j | Jannink 2010 |

**Supplementary Table 2.** Heat map table of relative genetic gain represented as a percentage of genetic response (Rs) relative to responses in phenotypic selection on centralized population with hub-network design (CE-PS-HN) in simulations for 400 simulated QTL responsible for 70% of phenotypic variability. Top 10% of the lines are selected from centralized and island populations as parental lines to be crossed using Hub Network (HN), Chain Rule (CR), Random Mating (RM) and Genomic Mating (GM) designs. Selection methods included Phenotypic Selection (PS), Genomic Selection (GS), and Weighted Genomic Selection (WGS) with Jannink weighting function. Migration policies included “Isolated” (DS), “Centralized” (CE), “Best Island” (BI), “Random Best” (RB), and “Fully Connected” (FC) policies with bi-directional migrations of two migrants every other cycle.

| **Mating Design** | | **Migration Policy** | **Selection Method** | **5** | **10** | | **20** | | **30** | | **40** | |
| --- | --- | --- | --- | --- | --- | --- | --- | --- | --- | --- | --- | --- |
| **Hub Network** | **Centralized** | | **PS** | **0 (0.16)** | | **0 (0.24)** | | **0 (0.29)** | | **0 (0.3)** | | **0 (0.3)** |
|  |  |  | **GS** | **12.5 (0.18)** | | **20.83 (0.29)** | | **20.69 (0.35)** | | **13.33 (0.34)** | | **16.67 (0.35)** |
|  |  |  | **WGS** | **-25 (0.12)** | | **-8.33 (0.22)** | | **10.34 (0.32)** | | **13.33 (0.34)** | | **16.67 (0.35)** |
|  | **Isolated** | | **PS** | **-50 (0.08)** | | **-58.33 (0.1)** | | **-65.52 (0.1)** | | **-66.67 (0.1)** | | **-66.67 (0.1)** |
|  |  |  | **GS** | **-43.75 (0.09)** | | **-54.17 (0.11)** | | **-62.07 (0.11)** | | **-63.33 (0.11)** | | **-63.33 (0.11)** |
|  |  |  | **WGS** | **-43.75 (0.09)** | | **-50 (0.12)** | | **-58.62 (0.12)** | | **-60 (0.12)** | | **-60 (0.12)** |
|  | **Best Island** | | **PS** | **-43.75 (0.09)** | | **-37.5 (0.15)** | | **-34.48 (0.19)** | | **-33.33 (0.2)** | | **-30 (0.21)** |
|  |  |  | **GS** | **-31.25 (0.11)** | | **-20.83 (0.19)** | | **0 (0.29)** | | **13.33 (0.34)** | | **20 (0.36)** |
|  |  |  | **WGS** | **-31.25 (0.11)** | | **-20.83 (0.19)** | | **3.45 (0.3)** | | **16.67 (0.35)** | | **23.33 (0.37)** |
|  | **Random Best** | | **PS** | **-37.5 (0.1)** | | **-33.33 (0.16)** | | **-34.48 (0.19)** | | **-33.33 (0.2)** | | **-33.33 (0.2)** |
|  |  |  | **GS** | **-25 (0.12)** | | **-16.67 (0.2)** | | **-3.45 (0.28)** | | **10 (0.33)** | | **16.67 (0.35)** |
|  |  |  | **WGS** | **-25 (0.12)** | | **-16.67 (0.2)** | | **0 (0.29)** | | **16.67 (0.35)** | | **20 (0.36)** |
|  | **Fully Connected** | | **PS** | **-31.25 (0.11)** | | **0 (0.24)** | | **48.28 (0.43)** | | **76.67 (0.53)** | | **86.67 (0.56)** |
|  |  |  | **GS** | **-12.5 (0.14)** | | **20.83 (0.29)** | | **82.76 (0.53)** | | **96.67 (0.59)** | | **96.67 (0.59)** |
|  |  |  | **WGS** | **-18.75 (0.13)** | | **20.83 (0.29)** | | **75.86 (0.51)** | | **96.67 (0.59)** | | **96.67 (0.59)** |
| **Chain Rule** | **Centralized** | | **PS** | **-25 (0.12)** | | **4.17 (0.25)** | | **55.17 (0.45)** | | **76.67 (0.53)** | | **86.67 (0.56)** |
|  |  |  | **GS** | **-12.5 (0.14)** | | **16.67 (0.28)** | | **51.72 (0.44)** | | **56.67 (0.47)** | | **56.67 (0.47)** |
|  |  |  | **WGS** | **-31.25 (0.11)** | | **0 (0.24)** | | **58.62 (0.46)** | | **93.33 (0.58)** | | **106.67 (0.62)** |
|  | **Isolated** | | **PS** | **-56.25 (0.07)** | | **-54.17 (0.11)** | | **-58.62 (0.12)** | | **-60 (0.12)** | | **-60 (0.12)** |
|  |  |  | **GS** | **-43.75 (0.09)** | | **-50 (0.12)** | | **-58.62 (0.12)** | | **-60 (0.12)** | | **-60 (0.12)** |
|  |  |  | **WGS** | **-43.75 (0.09)** | | **-50 (0.12)** | | **-58.62 (0.12)** | | **-60 (0.12)** | | **-60 (0.12)** |
|  | **Best Island** | | **PS** | **-43.75 (0.09)** | | **-37.5 (0.15)** | | **-24.14 (0.22)** | | **-20 (0.24)** | | **-16.67 (0.25)** |
|  |  |  | **GS** | **-37.5 (0.1)** | | **-25 (0.18)** | | **-3.45 (0.28)** | | **16.67 (0.35)** | | **26.67 (0.38)** |
|  |  |  | **WGS** | **-37.5 (0.1)** | | **-25 (0.18)** | | **-3.45 (0.28)** | | **13.33 (0.34)** | | **23.33 (0.37)** |
|  | **Random Best** | | **PS** | **-43.75 (0.09)** | | **-33.33 (0.16)** | | **-20.69 (0.23)** | | **-20 (0.24)** | | **-16.67 (0.25)** |
|  |  |  | **GS** | **-31.25 (0.11)** | | **-20.83 (0.19)** | | **6.9 (0.31)** | | **26.67 (0.38)** | | **33.33 (0.4)** |
|  |  |  | **WGS** | **-37.5 (0.1)** | | **-20.83 (0.19)** | | **3.45 (0.3)** | | **26.67 (0.38)** | | **33.33 (0.4)** |
|  | **Fully Connected** | | **PS** | **-31.25 (0.11)** | | **4.17 (0.25)** | | **55.17 (0.45)** | | **80 (0.54)** | | **90 (0.57)** |
|  |  |  | **GS** | **-18.75 (0.13)** | | **16.67 (0.28)** | | **75.86 (0.51)** | | **96.67 (0.59)** | | **100 (0.6)** |
|  |  |  | **WGS** | **-18.75 (0.13)** | | **16.67 (0.28)** | | **75.86 (0.51)** | | **93.33 (0.58)** | | **96.67 (0.59)** |
| **Random Mating** | **Centralized** | | **PS** | **-25 (0.12)** | | **0 (0.24)** | | **51.72 (0.44)** | | **76.67 (0.53)** | | **83.33 (0.55)** |
|  |  |  | **GS** | **-18.75 (0.13)** | | **12.5 (0.27)** | | **62.07 (0.47)** | | **80 (0.54)** | | **83.33 (0.55)** |
|  |  |  | **WGS** | **-31.25 (0.11)** | | **0 (0.24)** | | **51.72 (0.44)** | | **86.67 (0.56)** | | **103.33 (0.61)** |
|  | **Isolated** | | **PS** | **-56.25 (0.07)** | | **-58.33 (0.1)** | | **-62.07 (0.11)** | | **-63.33 (0.11)** | | **-63.33 (0.11)** |
|  |  |  | **GS** | **-50 (0.08)** | | **-54.17 (0.11)** | | **-62.07 (0.11)** | | **-63.33 (0.11)** | | **-63.33 (0.11)** |
|  |  |  | **WGS** | **-50 (0.08)** | | **-54.17 (0.11)** | | **-62.07 (0.11)** | | **-63.33 (0.11)** | | **-63.33 (0.11)** |
|  | **Best Island** | | **PS** | **-50 (0.08)** | | **-41.67 (0.14)** | | **-20.69 (0.23)** | | **-3.33 (0.29)** | | **10 (0.33)** |
|  |  |  | **GS** | **-37.5 (0.1)** | | **-29.17 (0.17)** | | **-3.45 (0.28)** | | **16.67 (0.35)** | | **26.67 (0.38)** |
|  |  |  | **WGS** | **-43.75 (0.09)** | | **-29.17 (0.17)** | | **-6.9 (0.27)** | | **16.67 (0.35)** | | **26.67 (0.38)** |
|  | **Random Best** | | **PS** | **-43.75 (0.09)** | | **-37.5 (0.15)** | | **-20.69 (0.23)** | | **-3.33 (0.29)** | | **6.67 (0.32)** |
|  |  |  | **GS** | **-37.5 (0.1)** | | **-20.83 (0.19)** | | **3.45 (0.3)** | | **20 (0.36)** | | **33.33 (0.4)** |
|  |  |  | **WGS** | **-37.5 (0.1)** | | **-25 (0.18)** | | **0 (0.29)** | | **13.33 (0.34)** | | **23.33 (0.37)** |
|  | **Fully Connected** | | **PS** | **-31.25 (0.11)** | | **0 (0.24)** | | **51.72 (0.44)** | | **83.33 (0.55)** | | **96.67 (0.59)** |
|  |  |  | **GS** | **-25 (0.12)** | | **12.5 (0.27)** | | **72.41 (0.5)** | | **96.67 (0.59)** | | **100 (0.6)** |
|  |  |  | **WGS** | **-18.75 (0.13)** | | **12.5 (0.27)** | | **72.41 (0.5)** | | **96.67 (0.59)** | | **100 (0.6)** |
| **Genomic Mating** | **Centralized** | | **PS** | **-18.75 (0.13)** | | **4.17 (0.25)** | | **41.38 (0.41)** | | **53.33 (0.46)** | | **56.67 (0.47)** |
|  |  |  | **GS** | **-12.5 (0.14)** | | **16.67 (0.28)** | | **51.72 (0.44)** | | **56.67 (0.47)** | | **56.67 (0.47)** |
|  |  |  | **WGS** | **-25 (0.12)** | | **0 (0.24)** | | **51.72 (0.44)** | | **73.33 (0.52)** | | **76.67 (0.53)** |
|  | **Isolated** | | **PS** | **-37.5 (0.1)** | | **-45.83 (0.13)** | | **-51.72 (0.14)** | | **-53.33 (0.14)** | | **-53.33 (0.14)** |
|  |  |  | **GS** | **-37.5 (0.1)** | | **-45.83 (0.13)** | | **-51.72 (0.14)** | | **-53.33 (0.14)** | | **-53.33 (0.14)** |
|  |  |  | **WGS** | **-37.5 (0.1)** | | **-41.67 (0.14)** | | **-51.72 (0.14)** | | **-53.33 (0.14)** | | **-53.33 (0.14)** |
|  | **Best Island** | | **PS** | **-50 (0.08)** | | **-41.67 (0.14)** | | **-13.79 (0.25)** | | **16.67 (0.35)** | | **46.67 (0.44)** |
|  |  |  | **GS** | **-37.5 (0.1)** | | **-29.17 (0.17)** | | **6.9 (0.31)** | | **46.67 (0.44)** | | **83.33 (0.55)** |
|  |  |  | **WGS** | **-37.5 (0.1)** | | **-25 (0.18)** | | **10.34 (0.32)** | | **50 (0.45)** | | **83.33 (0.55)** |
|  | **Random Best** | | **PS** | **-43.75 (0.09)** | | **-33.33 (0.16)** | | **0 (0.29)** | | **33.33 (0.4)** | | **60 (0.48)** |
|  |  |  | **GS** | **-37.5 (0.1)** | | **-20.83 (0.19)** | | **24.14 (0.36)** | | **66.67 (0.5)** | | **100 (0.6)** |
|  |  |  | **WGS** | **-37.5 (0.1)** | | **-20.83 (0.19)** | | **24.14 (0.36)** | | **66.67 (0.5)** | | **96.67 (0.59)** |
|  | **Fully Connected** | | **PS** | **-31.25 (0.11)** | | **-4.17 (0.23)** | | **41.38 (0.41)** | | **73.33 (0.52)** | | **90 (0.57)** |
|  |  |  | **GS** | **-18.75 (0.13)** | | **12.5 (0.27)** | | **65.52 (0.48)** | | **96.67 (0.59)** | | **103.33 (0.61)** |
|  |  |  | **WGS** | **-18.75 (0.13)** | | **8.33 (0.26)** | | **68.97 (0.49)** | | **96.67 (0.59)** | | **103.33 (0.61)** |

**Supplementary Table 3.** **Heat map of relative genetic** **gain** represented as a percentage of genetic response relative to responses in phenotypic selection on centralized populations with hub network design (CE-PS-HN) in simulations for 400 simulated QTL responsible for 70% of phenotypic variability. Top 10% of the lines are selected for the reference design (CE-PS-HN) for comparisons with responses provided in Supplementary Table 2 and top 20% of the lines are selected as parental lines to be crossed for the GM design in non-island and island populations. Selection Methods included Phenotypic Selection (PS), Genomic Selection (GS), and Weighted Genomic Selection (WGS) with Jannink weighting function. Mating designs included HN (Hub Network), GM- Genomic Mating method. Migration policies included the Best Island (BI), Random Best (RB), and Fully Connected (FC) topologies with bi-directional migrations of two migrants every other cycle.

|  |  |  | **Cycle Number** | | | | |
| --- | --- | --- | --- | --- | --- | --- | --- |
| **Mating Design (Selected Fraction)** | **Migration Policy** | **Selection Method** | **5** | **10** | **20** | **30** | **40** |
| Hub Network - Top 10% | Centralized | PS | 0 | 0 | 0 | 0 | 0 |
| Genomic Mating -Top 20% | Centralized | PS | -36.32 | -14.1 | 37.18 | 75.07 | 94.64 |
|  |  | GS | -23.59 | -1.85 | 50.9 | 88.53 | 97.99 |
|  |  | WGS | -36.32 | -18.2 | 33.75 | 78.43 | 108.1 |
|  | Best Island | PS | -55.43 | -50.9 | -24.6 | 4.37 | 34.23 |
|  |  | GS | -49.06 | -38.7 | -10.8 | 31.3 | 64.43 |
|  |  | WGS | -42.69 | -38.7 | -7.41 | 31.3 | 64.43 |
|  | Random Best | PS | -49.06 | -42.8 | -14.3 | 17.83 | 51.01 |
|  |  | GS | -42.69 | -34.6 | 2.88 | 44.77 | 81.21 |
|  |  | WGS | -42.69 | -30.5 | 2.88 | 48.13 | 84.57 |
|  | Fully Connected | PS | -36.32 | -22.3 | 30.32 | 71.7 | 101.4 |
|  |  | GS | -29.95 | -10 | 44.04 | 91.9 | 114.8 |
|  |  | WGS | -29.95 | -10 | 40.61 | 85.17 | 111.4 |
